# Anxiety and its risk factors among non-Japanese residents living in Japan undergoing COVID-19 situation: A cross-sectional survey

**DOI:** 10.1371/journal.pone.0280144

**Published:** 2024-03-15

**Authors:** Mai Ngoc Luu, Atsuko Imoto, Yoshimi Matsuo, Nguyen Tien Huy, Ahmad Qarawi, Shamael Thabit Mohammed Alhady, Le Van Truong, Ryuji Yoshino, Nguyen Tran Minh Duc, Kozue Tabei, Yixiao Lu, Manmeet Kaur Singh, Mai Phuong Truong, Shyam Prakash Dumre, Ian Christopher Naungayan Rocha, I-Chun Hung, Akane Fudo, Miho Sato, Sajog Kansakar, Akiko Tsukamoto, Aiko Komatsu, Guoxi Cai, Kazuhiko Moji, Thanawat Khongyot, Saruveish Mogan, Souksavath Soukdavone, Endah Dwi Hartuti, Kounnavong Thidatheb, Shiho Honda, Hyunjae Woo, Nitu Lama, Vy Thi Nhat Huynh, Huynh Le Anh Khoa, Kirellos Said Abbas, Fatma A. Monib, Hoda Aly Mohamed Omran, Chiristine Samuel Rezq, Mostafa Shehata Qatora, Sze Jia Ng, Graca Jaqueline Vanessa Morena, Adriana Viola Miranda, Minh-Trang Ngo Huynh, Junko Ota, Kim Minjung, Jaemin An, Latdavanh Vorlasane, Kesshinee Gunasegaran, Fazureen Zulkefli, Belen de Jesús Lima Girón, Punita Gauchan Bhattachan, Renu Bhandari Dumre, Kishor Pandey, Sarina Yamashita, Aden Kay Celis Seposo, Jayson Zabala, Adrián Riva-Moscoso, Joyce Nicole Pineda Ordóñez, Suriyon Uitrakul, Fortunato S. Principe-Meneses, Kadek Agus Surya Dila

**Affiliations:** 1 Department of Internal Medicine, University of Medicine and Pharmacy at Ho Chi Minh City, Ho Chi Minh City, Vietnam; 2 School of Tropical Medicine and Global Health, Nagasaki University, Nagasaki, Japan; 3 NPO Treasures of The Planet, Japan; 4 Essen Healthcare, Bronx, NY, United States of America; 5 Faculty of Medicine, University of Gezira, Wad Medani, Sudan; 6 Traditional Medicine Hospital, Ministry of Public Security, Hanoi, Vietnam; 7 School of Global Humanities and Social Sciences, Nagasaki University, Nagasaki, Japan; 8 Faculty of Medicine, University of Medicine and Pharmacy at Ho Chi Minh City, Ho Chi Minh City, Vietnam; 9 Save the Children Japan, Japan; 10 Department of Public Health, Nagasaki University Graduate School of Biomedical Sciences, Nagasaki, Japan; 11 American University of the Caribbean Medical School, Cupe Coy, Sint Maarten; 12 Central Department of Microbiology, Tribhuvan University, Kathmandu, Nepal; 13 School of Medicine, Centro Escolar University, Manila, Philippines; 14 Online Research Club, Nagasaki, Japan; 15 Independent Researcher, Nagasaki Prefecture, Japan; 16 Department of Internal Medicine, Maimonides Medical Center, Brooklyn, New York, United States of America; 17 Nagasaki Prefectural Institute of Environment and Public Health, Nagasaki, Japan; 18 School of Pharmacy, Walailak University, Nakhon Si Thammarat, Thailand; 19 Faculty of Medicine and Health Science, Universiti Malaysia Sarawak, Kota Samarahan, Sarawak, Malaysia; 20 Graduate School of Biomedical Sciences, Program for Nurturing Global Leader, Nagasaki University, Nagasaki, Japan; 21 Nagasaki International Student Support Center, Nagasaki University, Nagasaki, Japan; 22 Department of Cardiopulmonary Rehabilitation Science, Nagasaki University Graduate School of Biomedical Sciences, Nagasaki, Japan; 23 Dr. M. V. Shetty College of Physiotherapy, Rajiv Gandhi University of Health Sciences, Mangalore, India; 24 University of Debrecen, Debrecen, Hungary; 25 Department of Biostatistics, Virginia Commonwealth University School of Medicine, Richmond, VA, United States of America; 26 Faculty of Medicine, Alexandria University, Alexandria, Egypt; 27 Faculty of Medicine, Assiut University, Assiut, Egypt; 28 Faculty of Medicine, Crozer-Chester Medical Center, Upland, Pennyslvania, United States of America; 29 Faculty of Medicine, Trisakti University, Jakarta, Indonesia; 30 America Evangelical University, Los Angeles, CA, United States of America; 31 Faculty of Medicine, University of Indonesia, Jakarta, Indonesia; 32 University of Medicine and Pharmacy at Ho Chi Minh City, Ho Chi Minh City, Vietnam; 33 Independent Researcher, Nagasaki, Japan; 34 Osaka Ohtani University, Osaka, Japan; 35 Savannakhet Provincial Hospital, Savannakhet, Lao PDR; 36 School of Medicine, University Putra Malaysia, Malaysia, Serdang, Selangor, Malaysia; 37 School of Medicine, Autonomous University of Baja California, Mexicali, México; 38 Graduate School of Biomedical Sciences, Center for Bioinformatics and Molecular Medicine, Nagasaki University, Nagasaki, Japan; 39 Central Department of Zoology, Tribhuvan University, Kathmandu, Nepal; 40 School of Medicine, Nagasaki University, Nagasaki, Japan; 41 Quezon City Science High School, Naruto University of Education, Naruto, Japan; 42 School of Medicine, Universidad Peruana de Ciencias Aplicadas (UPC), Lima, Peru; 43 Faculty of Medicine, Catholic University of Honduras, Tegucigalpa, Honduras; 44 Giri Emas Hospital, Singaraja City, Buleleng, Bali, Indonesia; Taipei Medical University, TAIWAN

## Abstract

**Introduction:**

In the context of collective efforts taken in Japan to control the spread of COVID-19, the state of emergency and social distancing have caused a negative impact on the mental health of all residents, including foreign communities in Japan. This study aimed to evaluate the level of anxiety and its associated factors among non-Japanese residents residing in Japan during the COVID-19 pandemic.

**Methods:**

A web-based survey in 13 languages was conducted among non-Japanese residents living in Japan during the COVID-19 situation. The State-Trait Anxiety Inventory assessed the level of anxiety–State (STAI-S) scores prorated from its six-item version. The multivariable logistic regression using the Akaike Information Criterion (AIC) method was performed to identify the associated factors of anxiety among participants.

**Results:**

From January to March 2021, we collected 392 responses. A total of 357 valid responses were analyzed. 54.6% of participants suffered from clinically significant anxiety (CSA). In multivariable logistic model analysis, the CSA status or the high level of anxiety was associated with three factors, including having troubles/difficulties in learning or working, decreased sleep duration, and decreased overall physical health (p<0.05).

**Conclusion:**

Our study suggests several possible risk factors of anxiety among non-Japanese residents living in Japan undergoing the COVID-19 pandemic, including the troubles or difficulties in learning or working, the decrease in sleep duration, and the decrease in overall physical health.

## Introduction

Coronavirus Disease 2019, also known as COVID-19, was first detected in Wuhan, China, in December 2019 [1]. Since discovering the first cases, COVID-19 has spread at unprecedented rates, causing a highly transmissible pandemic worldwide. The disease has caused negative impacts not only on mental and physical health but also on many other aspects of social life. In the context of collective efforts taken in Japan, from social distancing to non-essential business shut-down to prevent the spread of COVID-19 in the community, many people are facing job loss, social quarantine or isolation, and deteriorating domestic relationships. According to a survey conducted in April and May 2020 by researchers at Waseda University and Osaka School of International Public Policy, the Japanese population was "particularly vulnerable" to mental health crises during the COVID-19 pandemic [2].

The cessation of businesses resulted in economic decline and increased the risk of developing depression and anxiety in the community. Furthermore, individuals in younger age groups felt the burden of stress from their parents during the pandemic and consequently were at risk for mental health problems [2]. In March 2020, Japan instituted its travel restrictions to contain the transmission of the disease [3]. The Japanese Government implemented strict guidelines to prevent entry into and exit from Japan, thus posing uncertainty in the expiration of immigration status or whether to leave the country [3]. Questions arose regarding the mental health of foreigners being held indefinitely in Japan due to the travel restrictions. In recent years, there has been a substantial increase in the presence of non-Japanese residents. As of December 2019, there is a record of 2.93 million foreigners living in Japan, driven by the rise in technical trainees and international students [4]. Despite such a large population of non-Japanese residents, little research has been conducted on the impact of the pandemic on the mental health within this vulnerable population in Japan. Non-Japanese residents could be exposed to more significant risks of mental health problems by additional challenges such as limited access to information and government supports, economic hardship, and travel restrictions to their home countries. In this study, we aim to determine the influence that COVID-19 has played on the mental health of foreigners residing in Japan, a country they must now vaguely call home.

## Materials and methods

### Study design and participants

We conducted a cross-sectional and web-based survey from January to March 2021 among non-Japanese residents living in Japan during the COVID-19 pandemic, specifically those residing in Nagasaki City–one of the cosmopolitan cities in Japan. The inclusion criteria of the participants were non-Japanese residents aged 18 years and older living in Japan, regardless of their infectious state to COVID-19. Those who did not fully complete the survey questions assessing their anxiety level were excluded. The study was undertaken in accordance with the latest version of the Helsinki declaration and the CROSS checklist in the **S1 File** [5]. There were no limitations on the number of survey participants.

The questionnaire in 13 available languages was distributed to participants through the website https://universalaid.jp/covid2021/, a multilingual website where foreigners living in Japan can consult information and seek solutions to deal with their problems and worries under the COVID-19 situation. This website is developed and managed by the NPO Treasures of The Planet using funds from the Japan Network for Public Interest Activities (JANPIA) dormant deposits. The participants took the survey in their native language, if available, using the Surveymonkey online links found on the website. The survey consisted of 28 questions which took approximately 7–10 minutes to complete. The age of the participants was determined in the first question of the survey to screen participants who met the study criteria. The survey was delivered using convenience and snowball sampling approach. The NPO Treasures of The Planet and our research team at Nagasaki distributed study flyers through visits to locations, which included but were not limited to churches, restaurants, cafes, and companies in Nagasaki City. Participants were also recruited by the staff of Nagasaki University, who networked with international students residing in Japan, through emails and social media platforms (i.e., school/ university homepage, Facebook). A part of the data collected from this survey was published online on the above website to support non-Japanese residents of Nagasaki City under the COVID-19 pandemic [6].

### Ethics approval and consent to participate

The study was approved by the Ethics Committee of the School of Tropical Medicine and Global Health, Nagasaki University, Japan, in January 2021, Reference No. NU_TMGH_2021_155_1. All the survey respondents gave their consent to participate in this study by filling out an informed consent embedded on the first page of the questionnaire. The responses collected from this survey are confidential and were not revealed to any external entity. In addition, the survey was completely anonymous.

### Questionnaire design

The self-reported questionnaire consists of 28 questions on basic demographic information, immigration status, residency status of other family members, a list of difficulties that the participants might face under the COVID-19 pandemic, the changes in their personal life, their overall physical, mental, and emotional health since the outbreak. The short form of the state scale of Spielberger State-Trait Anxiety Inventory (STAI-6), including six items, was used to assess the state anxiety levels [7]. This abbreviated tool has been validated and proved to correlate well with the original 20-item inventory [7, 8]. The respondents self-reported each item in the STAI-6 using a 4-point Likert scale (1 to 4 points). This questionnaire was developed originally in English and piloted among 30 foreigners living in Japan, then revised accordingly to the feedback. We then translated the survey into 12 languages: Arabic, Chinese (simplified), Filipino, Indonesian, Japanese, Korean, Laos, Malaysian, Nepali, Spanish, Thai, and Vietnamese. Each translated language version was then proofread, back-translated, pretested, and amended if necessary, using the same methodology of our previous global study [9]. The English survey questionnaire and the 12 translated versions were detailed in the **S2 File**.

### Statistical analysis

The data was collected anonymously through the SurveyMonkey© platform and imported into an Excel spreadsheet. Only valid responses that completed all six items of the STAI-6 and more than 95% of survey items were included in the analysis. We used frequency tables for all the study variables and standard univariable statistics to describe the study population. We used the means and standard deviations for continuous variables, while the frequencies and percentages were used to define categorical variables. Before calculating the scores of STAI-6, we reverse scores for three items ("I feel calm", "I am relaxed", and "I feel content"), then sum up the scores of relevant items. The STAI-6 scores range from 6 to 24. To assess the level of state anxiety, the STAI-6 scores were multiplied by 20/6 to get the prorated scores comparable with the 20-item state scale of the original STAI tool (STAI-S) [10, 11]. A cut-off score of 44 was used to indicate clinically significant anxiety (CSA) or a high level of anxiety [12–14].

In descriptive statistical analysis, Pearson’s chi-square test with Yates’ continuity correction if pertinent was performed to compare the characteristics of the CSA and non-CSA groups. The multivariable logistic regression using the Akaike Information Criterion (AIC) method was performed to identify the factors associated with the CSA status. The optimal model was selected based on the AIC index after checking all possible models in both backward and forward stepwise. The significance of the study was assessed at the probability level of 0.05. All data analyses were performed using the CompareGroups and MASS packages on R statistical software (version 3.6.3 for Windows).

## Results

### Characteristics of study participants

Among 485 survey-accessed respondents, 392 responses were collected. After cleaning the data, a total of 357 valid responses were analyzed.

The baseline sociodemographic characteristics of participants were summarized in **Table 1**. The male/female ratio was 1/1.1. Most of the respondents (87.4%) were 18–44 years of age. Of the survey respondents, 82.9% of respondents reported that their other family members lived in their home country, while 9.0% had family members who resided in Japan.

**Table 1:** Baseline characteristics of participants.

Baseline characteristics	Non-CSA	CSA	Total	P-value^#^
	(N = 162)	(N = 195)	(N = 357)	
	N (%)	N (%)	N (%)	
**Gender** (n = 352)				0.160
Female	79 (48.8)	108 (56.8)	187 (53.1)	
Male	83 (51.2)	82 (43.2)	165 (46.9)	
**Living location** (n = 350)				**0.033** ^*^
Nagasaki City	89 (54.9)	77 (41.0)	166 (47.4)	
Other cities of Nagasaki Prefecture	24 (14.8)	37 (19.7)	61 (17.4)	
Other prefectures	49 (30.2)	74 (39.4)	123 (35.1)	
**Age group** (n = 356)				**0.024** ^*^
18–24	39 (24.1)	63 (32.5)	102 (28.7)	
25–34	61 (37.7)	76 (39.2)	137 (38.5)	
35–44	31 (19.1)	41 (21.1)	72 (20.2)	
45–54	21 (13.0)	10 (5.15)	31 (8.71)	
55–64	8 (4.94)	3 (1.55)	11 (3.09)	
> = 65	2 (1.23)	1 (0.52)	3 (0.84)	
**Marital status** (n = 353)				0.140
Single	96 (59.6)	130 (67.7)	226 (64.0)	
Divorced	2 (1.24)	5 (2.60)	7 (1.98)	
Married or domestic partnership	63 (39.1)	57 (29.7)	120 (34.0)	
**Spouse/partner’s nationality** (n = 338)				**0.028** ^*^
No spouse/partner	76 (49.4)	107 (58.2)	183 (54.1)	
Japanese	33 (21.4)	20 (10.9)	53 (15.7)	
Same nationality	43 (27.9)	50 (27.2)	93 (27.5)	
Other nationality	2 (1.30)	7 (3.80)	9 (2.66)	
**Occupation** (n = 350)				0.376
Full-time employee	49 (30.8)	54 (28.3)	103 (29.4)	
Part-time employee	11 (6.92)	9 (4.71)	20 (5.71)	
Self-employed	6 (3.77)	4 (2.09)	10 (2.86)	
Housewife/husband	5 (3.14)	5 (2.62)	10 (2.86)	
Unemployed	0 (0.00)	4 (2.09)	4 (1.14)	
Student	88 (55.3)	115 (60.2)	203 (58.0)	
**Education level** (n = 352)				**0.021** ^*^
Post-graduation or higher	77 (48.1)	62 (32.3)	139 (39.5)	
College/university degree	73 (45.6)	114 (59.4)	187 (53.1)	
Senior high school	8 (5.00)	13 (6.77)	21 (5.97)	
Junior high school	2 (1.25)	3 (1.56)	5 (1.42)	
**Immigration status** (n = 353)				**0.004** ^*^
Naturalized	2 (1.23)	0 (0.00)	2 (0.57)	
Permanent resident	26 (16.0)	20 (10.5)	46 (13.0)	
Work-permit	28 (17.3)	47 (24.6)	75 (21.2)	
Student visa	89 (54.9)	118 (61.8)	207 (58.6)	
Family dependent visa	15 (9.26)	4 (2.09)	19 (5.38)	
Temporary visa (tourists, business.)	2 (1.23)	2 (1.05)	4 (1.13)	
**Type of health insurance** (n = 350)				0.406
National Health Insurance	116 (72.0)	140 (74.1)	256 (73.1)	
Employees’ Health Insurance	42 (26.1)	40 (21.2)	82 (23.4)	
Private Health Insurance	2 (1.24)	6 (3.17)	8 (2.29)	
None	1 (0.62)	3 (1.59)	4 (1.14)	
**Period of time living in Japan** (n = 355)				0.064
less than one year	23 (14.3)	19 (9.79)	42 (11.8)	
1–2 years	44 (27.3)	52 (26.8)	96 (27.0)	
3–5 years	41 (25.5)	76 (39.2)	117 (33.0)	
5–10 years	23 (14.3)	21 (10.8)	44 (12.4)	
more than 10 years	30 (18.6)	26 (13.4)	56 (15.8)	
**Japanese level** (n = 356)				0.351
I can speak on the same level as Japanese people	21 (13.0)	21 (10.8)	42 (11.8)	
I can speak well enough for work or study	48 (29.6)	58 (29.9)	106 (29.8)	
I can speak well enough to have no trouble in everyday life	43 (26.5)	69 (35.6)	112 (31.5)	
I can’t speak Japanese very well	32 (19.8)	31 (16.0)	63 (17.7)	
I can’t speak Japanese at al.	18 (11.1)	15 (7.73)	33 (9.27)	
**Total number of people living with** (n = 357)				0.485
No	64 (39.5)	93 (47.7)	157 (44.0)	
1	47 (29.0)	50 (25.6)	97 (27.2)	
2	18 (11.1)	25 (12.8)	43 (12.0)	
3	21 (13.0)	17 (8.72)	38 (10.6)	
4	11 (6.79)	9 (4.62)	20 (5.60)	
≥ 5	1 (0.62)	1 (0.51)	2 (0.56)	
**Number of people > = 65 living with** (n = 357)				0.830
No	152 (93.8)	182 (93.3)	334 (93.6)	
1	5 (3.09)	7 (3.59)	12 (3.36)	
2	2 (1.23)	1 (0.51)	3 (0.84)	
3	1 (0.62)	3 (1.54)	4 (1.12)	
4	2 (1.23)	1 (0.51)	3 (0.84)	
≥ 5	0 (0.00)	1 (0.51)	1 (0.28)	

Numbers in the parentheses indicate percentage (%) unless indicated otherwise. CSA: clinically significant anxiety.

^#^P value for the chi-square test with Yates’ correction for continuity.

*P value of less than 0.05 was considered statistically significant.

### Personal problems

**Table 2** illustrates the recent or current problems reported by the survey participants. The three most common problems reported among the participants were decreased personal income, difficulties in learning or working, and the discriminated feeling of being a foreigner, with rates of 43.4%, 36.7%, and 32.1%, respectively. A total of 52.9% of participants reported moderate to high fear of contracting COVID-19.

**Table 2:** The recent or current problems reported by the survey participants during the COVID-19 pandemic.

Personal problems	Non-CSA	CSA	Total	P-value^#^
	(N = 162)	(N = 195)	(N = 357)	
	N (%)	N (%)	N (%)	
**Having family member with COVID-19** (n = 354)	8 (4.94)	6 (3.12)	14 (3.95)	0.550
**Having family member with suspected to have COVID-19 but not tested** (n = 351)	3 (1.86)	7 (3.68)	10 (2.85)	0.354
**Having troubles/difficulties with learning or working** (n = 343)	34 (21.4)	92 (50.0)	126 (36.7)	**<0.001** ^*^
**Feeling discriminated for being a non-Japanese** (n = 336)	38 (24.2)	70 (39.1)	108 (32.1)	**0.005** ^*^
**Playing game/spending time on smartphone/TV** (n = 342)	9 (5.59)	35 (19.3)	44 (12.9)	**<0.001** ^*^
**Physical activities/doing exercise** (n = 351)	1 (0.62)	0 (0.00)	1 (0.28)	0.456
**Losing job** (n = 347)	4 (2.53)	3 (1.59)	7 (2.02)	0.706
**Having domestic violence at home** (n = 342)	16 (10.1)	18 (9.84)	34 (9.94)	1.000
**I am afraid of getting COVID-19 infection** (n = 357)				**0.001** ^*^
Not at all	27 (16.7)	19 (9.74)	46 (12.9)	
Somewhat	65 (40.1)	57 (29.2)	124 (34.2)	
Moderately	45 (27.8)	58 (29.7)	103 (28.9)	
Very much	25 (15.4)	61 (31.3)	86 (24.1)	
**Decrease in personal income** (n = 297)	21 (21.7)	49 (46.2)	70 (23.6)	**<0.001** ^*^

Numbers in the parentheses indicate percentage (%) unless indicated otherwise. COVID-19: Coronavirus Disease 2019; CSA: clinically significant anxiety.

^#^P value for the chi-square test with Yates’ correction for continuity.

^*^P value of less than 0.05 was considered statistically significant.

### Changes in living habits, physical, mental, and emotional health

**Table 3** summarizes the changes in living habits and survey respondents’ overall physical, mental, and emotional health under the COVID-19 pandemic. Approximately one-third of the participants reported a decrease in their overall physical, mental or emotional health.

**Table 3:** The changes in living habits and the overall physical, mental, and emotional health.

Changes in living habits and health	Non-CSA	CSA	Total	P-value^#^
	(N = 162)	(N = 195)	(N = 359)	
	N (%)	N (%)	N (%)	
**Sleep duration** (n = 331)				**<0.001** ^*^
Less than before	26 (17.4)	74 (40.7)	100 (30.2)	
Same as before	109 (73.2)	77 (42.3)	186 (56.2)	
More than before	14 (9.40)	31 (17.0)	45 (13.6)	
**Body weight** (n = 332)				0.054
Less than before	21 (13.5)	37 (20.9)	58 (17.5)	
Same as before	95 (61.3)	86 (48.6)	181 (54.5)	
More than before	39 (25.2)	54 (30.5)	93 (28.0)	
**Alcohol consumption** (n = 201)				0.078
Less than before	24 (26.1)	41 (37.6)	65 (32.3)	
Same as before	56 (60.9)	49 (45.0)	105 (52.2)	
More than before	12 (13.0)	19 (17.4)	31 (15.4)	
Smoking (n = 83)				0.136
Less than before	6 (16.7)	9 (19.1)	15 (18.1)	
Same as before	26 (72.2)	25 (53.2)	51 (61.4)	
More than before	4 (11.1)	13 (27.7)	17 (20.5)	
**Playing game/spending time on smartphone/TV** (n = 312)				**<0.001** ^*^
Less than before	13 (9.85)	9 (5.00)	22 (7.05)	
Same as before	68 (51.5)	55 (30.6)	123 (39.4)	
More than before	51 (38.6)	116 (64.4)	167 (53.5)	
**Physical activities/doing exercise** (n = 337)				**<0.001** ^*^
Less than before	61 (40.4)	111 (59.7)	172 (51.0)	
Same as before	69 (45.7)	46 (24.7)	115 (34.1)	
More than before	21 (13.9)	29 (15.6)	50 (14.8)	
**Duration of parenting or childcare** (n = 97)				0.217
Less than before	5 (9.26)	9 (20.9)	14 (14.4)	
Same as before	35 (64.8)	22 (51.2)	57 (58.8)	
More than before	14 (25.9)	12 (27.9)	26 (26.8)	
**Conflict/quarrel in your home** (n = 122)				0.144
Less than before	9 (18.0)	15 (20.8)	24 (19.7)	
Same as before	35 (70.0)	39 (54.2)	74 (60.7)	
More than before	6 (12.0)	18 (25.0)	24 (19.7)	
**Communication with neighbors or friends** (n = 283)				**0.001** ^*^
Less than before	49 (38.0)	87 (56.5)	136 (48.1)	
Same as before	68 (52.7)	47 (30.5)	115 (40.6)	
More than before	12 (9.30)	20 (13.0)	32 (11.3)	
**Overall mental health** (n = 323)				**<0.001** ^*^
Less than before	15 (9.87)	85 (49.7)	100 (31.0)	
Same as before	128 (84.2)	68 (39.8)	196 (60.7)	
More than before	9 (5.92)	18 (10.5)	27 (8.36)	
**Overall physical health** (n = 330)				**<0.001** ^*^
Less than before	24 (15.4)	75 (43.1)	99 (30.0)	
Same as before	119 (76.3)	82 (47.1)	201 (60.9)	
More than before	13 (8.33)	17 (9.77)	30 (9.09)	
**Overall emotional health (happiness)** (n = 337)				**<0.001** ^*^
Less than before	22 (14.2)	111 (61.0)	133 (39.5)	
Same as before	114 (73.5)	55 (30.2)	169 (50.1)	
More than before	19 (12.3)	16 (8.79)	35 (10.4)	

Numbers in the parentheses indicate percentage (%) unless indicated otherwise. CSA: clinically significant anxiety.

^#^P value for the chi-square test with Yates’ correction for continuity.

^*^P value of less than 0.05 was considered statistically significant.

### CSA and associated risk factors

The mean STAI-S scores were 46.7±12.3. The prevalence of CSA among survey participants was 54.6% (195/357).

In multivariable logistic model analysis, the CSA status or the high level of state anxiety was associated with three factors: having troubles/difficulties in learning or working, the decrease in sleep duration, and the decrease in overall physical health (p<0.05) (**Table 4**).

**Table 4:** Multivariable logistic model analysis for the CSA status.

Predictors	Univariable			Multivariable		
	OR	95% CI	P value	OR	95% CI	P value
**(Intercept)**	-	-	-	0.17	0.05–0.49	0.002
**Having family member with suspected to have COVID-19 but not tested**						
No	*Reference*			*Reference*		
Yes	1.81	0.35–13.30	0.497	4.11	0.63–35.32	0.152
**Having troubles/difficulties with learning or working**						
No	*Reference*			*Reference*		
Yes	4.15	2.27–7.80	**<0.001** ^*^	3.66	1.89–7.28	**<0.001** ^*^
**Being afraid of contracting COVID-19**						
Not at all	*Reference*			*Reference*		
Somewhat	1.07	0.43–2.77	0.884	1.27	0.42–4.04	0.673
Moderately	2.35	0.91–6.26	0.08	3.1	1.02–10.05	0.051
Very much	2.7	1.04–7.30	**0.045** ^*^	2.62	0.84–8.51	0.101
**Sleep duration**						
Same as before	*Reference*			*Reference*		
Less than before	4.87	2.50–9.90	**<0.001** ^*^	3.39	1.56–7.62	**0.002** ^*^
More than before	2.03	0.89–4.75	0.096	2.02	0.80–5.21	0.138
**Overall physical health**						
Same as before	*Reference*			*Reference*		
Less than before	3.41	1.84–6.51	**<0.001**	2.24	1.08–4.73	**0.031** ^*^
More than before	1.52	0.57–4.08	0.399	1.39	0.44–4.36	0.569
**Observations**	-			212		
**R2 Tjur**	-			0.239		

CI: confidence interval; COVID-19: Coronavirus Disease 2019; STAI-S: Spielberger State-Trait Anxiety Inventory–State.

^*^P value of less than 0.05 was considered statistically significant.

## Discussion

This cross-sectional web-based survey provided evidence for the high prevalence of anxiety among non-Japanese residents in Japan under the COVID-19 pandemic. The associated risk factors of anxiety included having troubles/difficulties in learning or working, decreased sleep duration, and decreased physical health.

The study showed that 54.6% of participants suffered from CSA or a high level of anxiety, reflecting the enormous psychological impact of the COVID-19 outbreak on foreigners residing in Japan. This result accords with a recent survey of 497 Chinese residents in Japan, wherein 66.6% of respondents indicated having heightened anxiety [15]. This prevalence was much higher than what was found in local Japanese residents, with 10.9% suffering from pandemic-related anxiety, demonstrating that foreign residents were more vulnerable to anxiety disorders during the COVID-19 pandemic compared to native residents [16]. However, this comparison might be unreliable due to the different screening tools used in these studies. Recently, a large-scale cross-sectional study among the general adult population residing in Japan which also used the cut-off STAI-S score of 44 to define the high level of anxiety, reported that up to 86.1% of participants suffered from moderate to severe symptoms of anxiety [14]. This result suggests that the COVID-19 pandemic might have a higher psychological impact on native residents compared to foreigners in Japan.

The present study revealed that the level of anxiety among foreigners living in Japan during the COVID-19 crisis, which was measured by the STAI-S scores, was significantly associated with their troubles/difficulties in learning or working. Up to one-third of our survey participants reported facing difficulties in learning or working. A longitudinal study evaluating the effects of different COVID stressors among first-year students revealed that distanced learning difficulties contributed to increased anxiety symptoms [17]. Besides the cultural and language barriers, foreign residents in Japan have to overcome many obstacles in online studying or working from home, and some even lost their jobs during the COVID-19 lockdown. These challenges may have a negative impact on their mental health and anxiety levels. A recent report from the Japanese Government revealed that under the ongoing COVID situation, non-permanent workers and unemployed individuals were particularly vulnerable to mental health disorders [2]. Unemployment due to COVID-19 was reported to negatively affect the mental health of workers in Japan [18].

The relationship between the fear of COVID-19 and anxiety symptoms has been mentioned in previous studies [19–21]. The fear was not distributed equally across the area but highly manifested where a high density of COVID-19 cases was confirmed [22]. Higher levels of COVID-19 fear were found in foreign-born individuals [19]. Additionally, Asians, Hispanics, women, and families with children under 18 appeared to have a higher subjective fear than their counterparts [22]. In our study, athough univariable logistic regression analysis showed that non-Japanese residents with high levels of fear of getting COVID-19 had a significantly higher level of anxiety, this association did not reach statistical significance in multivariable analysis. However, the finding still somewhat warned of the possibility that the mental health of foreigners in Japan was being threatened under the COVID-19 crisis. The fear of contracting COVID-19 among non-residents may arise from the fear of physical health, the fear of infecting family members of beloved ones with the disease, the fear of not knowing essential information about COVID-19, and the fear of being passive, followed by the economic consequences [23].

In accordance with previous studies conducted on different populations, our study also indicated the association between decreased physical health as well as decreased sleep quality and anxiety among non-Japanese residents in Japan during the COVID-19 crisis. COVID-19 pandemic and lockdown policy were shown to have negative impacts on physical health, psychological health, physical activity, and overall well-being, even in the general community [24]. The impact of COVID-19 pandemic on physical health could be directly resulted from organ damage due to SARS-CoV-2 infection or indirectly due to physical inactivity and disruptions in daily routines related to isolation policies. Low physical activity levels were associated with the presence of anxiety and depression symptoms [25]. The decrease in sleep duration also had a negative impact on the level of anxiety. The common effects of physical health and sleep quality on mental health can explained these findings. Sleep duration and quality were reported to be independent predictors of physical health and, in particular, mental health in previous studies under the COVID-19 situation and also included as one of the seven criteria in a recent publication of the fear of COVID-19 Scale [26–29]. Until now, there has been no comparative data on the influence of decreased physical activity and decreased sleep quality on the risk of anxiety disorders between the native residents and foreigners in Japan. Thus, further research is needed to evaluate these associations.

Our study had several limitations. Firstly, the data were collected through a cross-sectional and web-based survey, and as a result, it could not provide a complete picture of anxiety levels among immigrants in Japan over different waves of the COVID-19 pandemic. Secondly, this study was limited to examining several anxiety symptoms using the short version of STAI-6 but did not cover all symptoms and other mental disorders among foreigners residing in Japan. Further studies are needed to evaluate the psychological impact of COVID-19 on these vulnerable groups. Despite these limitations, our study is the first to assess anxiety and its associated factors among multinational immigrants in Japan and can provide important information for social policies and interventions for mental health during the COVID pandemic.

## Conclusion

In conclusion, our study suggests several possible risk factors of anxiety among non-Japanese residents living in Japan during the COVID-19 crisis, including the troubles or difficulties in learning or working, the fear of contracting COVID-19, and the decrease in sleep duration, and the decrease in overall physical health.

## Supporting information

S1 File Checklist for Reporting Of Survey Studies (CROSS). (DOCX)

S2 File The English survey questionnaire and the 12 translated versions. (PDF)

S1 Data (XLSX)
