# Supplementary material for: Anxiety and its risk factors among non-Japanese residents living in Japan undergoing COVID-19 situation: A cross-sectional survey
Source: PLoS One. 2024 Mar 15;19(3):e0280144. doi: 10.1371/journal.pone.0280144 (PMC10942066; doi:10.1371/journal.pone.0280144)
Supplement: S2 File — (PDF) [file pone.0280144.s002.pdf]

Table of Contents

Arabic	2
Chinese	20
English	37
Filipino	55
Indonesian	71
Japanese	85
Korean	99
Laos	119
Malaysian	137
Nepali	156
Spanish	173
Thai	192
Vietnamese	210

مشكلات و قلق السكان الغير يابانيين المقيمين باليابان في ظل وضع جائحة فيروس كورونا المستجد (COVID-19): دراسة مستعرضة

نقوم نحن -يونيفيرسال ايد باليابان و جامعة نجازاكي- باجراء هذه الاستطلاع للتعرف على مشكلات و قلق السكان الغير يابانيين المقيمين باليابان (خاصة مدينة نجازاكي) الناتجين عن جائحة فيروس كورونا المستجد، ساعين لتقديم مقترحات و استشارات لعلاج هذا الاضطراب و القلق.

هذا الاستطلاع يحتوي على 28 سؤال و قد يستغرق بالتقريب 7-10 دقائق. لديك حرية الرفض او الانسحاب من المشاركة في اي وقت. الاجابات المجمعة من هذا الاستطلاع سرية و لن يتم كشفها تحت اي ظرف. بالاضافة الى ذلك، هذا الاستطلاع بلا اسم تماما و لا يوجد مخاطر مصاحبة.

للمشاركة في هذا الاستطلاع اضغط علي الرابط المصاحب:

https://universalaid.jp/survey2020/

للمزيد من الاستفسارات تستطيع التواصل مع فريقنا بارسال رسالة عبر البريد الالكتروني لاستاذة يوشيمي ماتسو، يونيفرسال ايد اليابان. (البريد الالكتروني : info@universalaid.jp;

الهاتف: 090-2900-3934)

او لدكتور نجوين تيان هوي، جامعة نجازاكي (البريد الالكتروني: tienhuy@nagasaki-u.ac.jp)

باكمال و ارسال هذا الاستطلاع انت تشير الى موافقتك على المشاركة في الاستطلاع.

1- هل عمرك يزيد على 18 عام و موافق على المشاركة في هذا المشروع البحثي؟

- ○ نعم
- ○ لا

قسم 1: معلومات عامة:

2- ما هي بلدك الاصلي؟

3- اين تسكن؟
- مدينة نجازاكي
- مدن اخرى في مقاطعة نجازاكي
- مقاطعة اخرى
- افضل عدم الاجابة

4- ما هي فئتك العمرية؟
- 18-24 عام
- 25-34 عام
- 35-44 عام
- 45-54 عام
- 55-64 عام
- 65 عاما او اكثر
- افضل عدم الاجابة

5- النوع:
- ذكر
- أنثى
- افضل عدم الاجابة
- أخر (من فضلك حدد)

6- الحالة اللاسرية:
- اعزب، لم اتزوج قط
- متزوج(ة) او تشارك منزلي
- ارمل(ة)
- مطلق(ة)
- منفصل(ة)

- افضل عدم الاجابة

7- ما هي الجنسية الاصلية للزوج(ة) \ الشريك(ة)؟

- ياباني(ة)
- نفس جنسيتي
- ليس لدي زوج(ة) او شريك(ة)
- افضل عدم الاجابة
- اخر

8- المهنة\ الوظيفة:

- موظف بدوام كامل
- موظف بنصف دوام\ عمل عرضي
- اعمل لحسابي الخاص
- رب(ة) منزل
- عاطل
- طالب
- متقاعد
- افضل عدم الاجابة
- اجابة اخرى (من فضلك حدد)

9- التعليم:

- متخرج\ دراسات عليا
- شهادة جامعية
- مدرسة ثانوية عليا
- مدرسة اعدادية
- مدرسة ابتدائية

- لا مدرسة
- افضل عدم الاجابة

10- حالة الهجرة:

- متجنس
- ساكن دائم
- تصريح عمل
- تأشيرة طالب
- تأشيرة معالة على الاسرة
- تأشيرة مؤقتة (سياحة، عمل)
- افضل عدم الاجابة
- اجابة اخرى (نرجو التحديد)

11- ما هو نوع تأمينك الصحي (اختر اجابة او اكثر)

- تأمين صحي وطني
- تأمين صحي للموظفين
- تأمين صحي خاص
- تأمين صحي للسفر
- لايوجد
- أفضل عدم الاجابة

12- كم من الوقت قضيت في اليابان؟

- أقل من عام
- عام-عامين
- 3-5 أعوام
- 6-10 أعوام
- أكثر من 10 أعوام
- أفضل عدم الاجابة

13- ما هو مستواك في اللغة اليابانية؟
- ○ استطيع التحدث باليابانية كالسكان اليابانيين
- ○ استطيع التحدث بما يكفي للعمل او الدراسة
- ○ استطيع التحدث بما يكفي الا اجد صعوبات في الحياة اليومية
- ○ لا استطيع التحدث باليابانية جيدا
- ○ لا استطيع التحدث باليابانية اطلاقا
- ○ أفضل عدم الاجابة

14- عدد السكان بمنزلك \ غرفتك (بمن فيهم انت):
- ○ 1
- ○ 2
- ○ 3
- ○ 4
- ○ 5 او اكثر

15- عدد السكان معك من الكبار بسن 65 او اكثر:
- ○ 0
- ○ 1
- ○ 2
- ○ 3
- ○ 4
- ○ 5 او اكثر

16- اين يسكن باقي افراد العائلة (الوالدين-الأخوات-الإخوة) حاليا؟ (إختر إجابة أو أكثر)
- ☐ ليس لدي أفراد عائلية آخرين
- ☐ معي في اليابان
- ☐ في منزل آخر في اليابان
- ☐ بوطننا الاصلي
- ☐ دول اخرى
- ☐ لا اعرف
- ☐ افضل عدم الاجابة

17- متى كانت اخر مرة رأيت فيها افراد عائلتك الاخرين (الوالدين- الأخوات- الاخوة)؟

- أقل من عام
- عام- عامين
- 3-5 اعوام
- 6-10 اعوام
- اكثر من 10 اعوام
- افضل عدم الاجابة

18- كم مرة تتصل بافراد عائلتك (والدين- اخوات –اخوة)؟

- يوميا
- اسبوعيا
- شهريا
- عدة مرات في السنة
- لا اتذكر
- لا اتصل ابدا
- افضل عدم الاجابة

القسم 2: شبكة المعلومات

19. هل لديك أي شخص لتشاركه مشاكلك؟

- نعم
- لا
- افضل عدم الاجابة

20. مع من يمكنك مشاركة مشاكلك؟ (حدد واحدًا أو أكثر)

- ☐ أمي
- ☐ أبي
- ☐ أختي
- ☐ أخي

☐ ابن عمي

☐ أقارب آخرون

☐ مدرس

☐ الزملاء

☐ صديق ياباني

☐ صديق من وطني

☐ زعيم ديني

☐ مستشار حكومي

☐ تفضل عدم الإجابة

☐ غير ذلك (يرجى التحديد)

21. من أين تحصل على المعلومات حول كوفيد-19؟ (حدد واحدًا أو أكثر)

☐ عائلة في بلدك

☐ عائلة في اليابان

☐ أصدقاء يابانيين

☐ أصدقاء من نفس البلد

☐ المنظمات أو المرافق أو المتاجر التي يتجمع فيها أشخاص من نفس البلد

☐ مكاتب البلدية والمحافظات

☐ مجلات المعلومات العامة والمواقع الإلكترونية الخاصة بالبلديات والمحافظات

☐ التبادل الدولي NPO

☐ التلفاز أو الجرائد أو المجلات أو الإنترنت

☐ مواقع التواصل الإجتماعى (Twitter و Facebook و Instagram وما إلى ذلك)

☐ لا أعرف كيف أحصل على المعلومات

☐ أفضل عدم الإجابة

▢ غير ذلك (يرجى التحديد)

22. ما نوع المعلومات التي تريدها بلغتك؟

▢ماذا أفعل في حالة الاشتباه في وجود أعراض لـ كوفيد-19
▢ماذا أفعل في حالة المرض أو الإصابات بخلاف كوفيد-19 في فترة كوفيد-19هذه
▢ماذا أفعل في حالة وقوع كارثة
▢ماذا أفعل عند الذهاب إلى المستشفيات / العيادات
▢ماذا أفعل في حالة وجود مشاكل مع الجيران أو العنف المنزلي أو التحرش الجنسي
▢الولادة والإنجاب
▢ماذا أفعل إذا كان طفلي يعاني من مشكلة
▢التعليم والمدارس للأطفال
▢حالة مكافحة العدوى لـ كوفيد-19
▢أين توجد النقاط الساخنة لـ كوفيد-19
▢إجراءات وقائية ضد مرض كوفيد-19
▢المساعدة المالية المتعلقة بـ كوفيد-19
▢كيفية العودة إلى بلد الوطن
▢كيفية تمديد التأشيرة
▢كيف تتصل بالعائلة من اليابان
▢المواصلات العامة
▢كيفية الحصول على رخصة القيادة
▢ نظام الضرائب والمعاشات
▢أحداث المجتمع

▢خبرات البحث عن منزل للعيش فيه
▢معلومات وظيفية وخبرات فى البحث عن وظيفة
▢كيفية شراء تأمين صحي وطني و/ أو خاص
▢تفضل عدم الإجابة
▢غير ذلك (يرجى التحديد)

القسم 3 : اسئلة قصيرة:

23. هل مؤخرا؟

كان لديك أحد أفراد العائلة مصاب مؤكد بكوفيد-19؟
○نعم
○لا
○أفضل عدم الإجابة

كان لديك أحد أفراد العائلة باشتباه عدوى كوفيد-19 ولكن لم يتمكن من إجراء اختبار؟
○نعم
○لا
○أفضل عدم الإجابة

لديك مشاكل / صعوبات في التعلم أو العمل؟
○نعم
○لا
○أفضل عدم الإجابة

شعرت بالتمييز لمجرد كونك غير ياباني؟
○نعم
○لا
○أفضل عدم الإجابة

فقدت وظيفتك

نعم
لا
أفضل عدم الإجابة

كان لديك عنف منزلي في بيتك؟

نعم
لا
أفضل عدم الإجابة

تم التحرش بك جنسياً ؟

نعم
لا
أفضل عدم الإجابة

شعرت بمزيد من النشاط الروحي / الديني؟

نعم
لا
أفضل عدم الإجابة

القسم 4: التغيرات في الحياة الشخصية

24. صِف مشاعرك/ حالاتك المزاجية الأخيرة (أو الحالية)؟

أشعر بالهدوء

لا إطلاقاً
إلى حد ما
بشكل متوسط
جداً

أنا متوتر

لا إطلاقاً
إلى حد ما
بشكل متوسط
جداً

أشعر بالضيق
- لا إطلاقاً
- إلى حد ما
- بشكل متوسط
- جداً

انا مسترخي
- لا إطلاقاً
- إلى حد ما
- بشكل متوسط
- جداً

أشعر بالرضا
- . لا إطلاقاً
- إلى حد ما
- بشكل متوسط
- جداً

أنا قلق
- . لا إطلاقاً
- إلى حد ما
- بشكل متوسط
- جداً

شعرت بالوحدة
- لا إطلاقاً
- إلى حد ما
- بشكل متوسط
- جداً

شعرت بالعزلة
- لا إطلاقاً
- إلى حد ما

- بشكل متوسط
- جداً

أخشى الإصابة بعدوى كوفيد-19

- لا إطلاقاً
- إلى حد ما
- بشكل متوسط
- جداً

مستوى المشاكل الخاصة بك

مدة النوم

- أقل من السابق
- كما كان من قبل
- أعلى / أكثر من قبل
- لا ينطبق
- أفضل عدم الإجابة

وزن الجسم

- أقل من السابق
- كما كان من قبل
- أعلى / أكثر من قبل
- لا ينطبق
- أفضل عدم الإجابة

استهلاك الكحول

- أقل من السابق
- كما كان من قبل
- أعلى / أكثر من قبل
- لا ينطبق
- أفضل عدم الإجابة

التدخين

- أقل من السابق
- كما كان من قبل
- أعلى / أكثر من قبل
- لا ينطبق
- أفضل عدم الإجابة

ممارسة الألعاب / قضاء الوقت على الهاتف الذكي / التلفزيون

- أقل من السابق
- كما كان من قبل
- أعلى / أكثر من قبل
- لا ينطبق
- أفضل عدم الإجابة

الأنشطة البدنية / ممارسة الرياضة

- أقل من السابق
- كما كان من قبل
- أعلى / أكثر من قبل
- لا ينطبق
- أفضل عدم الإجابة

مدة الأبوة أو رعاية الطفل

- أقل من السابق
- كما كان من قبل
- أعلى / أكثر من قبل
- لا ينطبق
- أفضل عدم الإجابة

الصراع / الشجار في منزلك

- أقل من السابق
- كما كان من قبل
- أعلى / أكثر من قبل

○ لا ينطبق
○ أفضل عدم الإجابة

التواصل مع الجيران أو الأصدقاء

○ أقل من السابق
○ كما كان من قبل
○ أعلى / أكثر من قبل
○ لا ينطبق
○ أفضل عدم الإجابة

صحتك العقلية العامة

○ أقل من السابق
○ كما كان من قبل
○ أعلى / أكثر من قبل
○ لا ينطبق
○ أفضل عدم الإجابة

صحتك الجسدية العامة

○ أقل من السابق
○ كما كان من قبل
○ أعلى / أكثر من قبل
○ لا ينطبق
○ أفضل عدم الإجابة

صحتك العاطفية العامة (السعادة)

○ أقل من السابق
○ كما كان من قبل
○ أعلى / أكثر من قبل
○ لا ينطبق
○ أفضل عدم الإجابة

رضاك الجنسي العام

○ أقل من السابق

○ كما كان من قبل
○ أعلى / أكثر من قبل
○ لا ينطبق
○ أفضل عدم الإجابة

الدخل الشخصي

○ أقل من السابق
○ كما كان من قبل
○ أعلى / أكثر من قبل
○ لا ينطبق
○ أفضل عدم الإجابة

الاستقرار المالي العام لأسرتك

○ أقل من السابق
○ كما كان من قبل
○ أعلى / أكثر من قبل
○ لا ينطبق
○ أفضل عدم الإجابة

26. عدد الأطفال دون سن 18 الذين يعيشون معك.

○ 0
○ 1
○ 2
○ 3
○ 4
○ أكثر من 4

27. مستوى مشاكل أطفالك

مدة نوم الأطفال

- أقل من السابق
- كما كان من قبل
- أعلى / أكثر من قبل
- لا ينطبق
- أفضل عدم الإجابة

لعب الأطفال / قضاء الوقت على الهاتف الذكي / التلفزيون

- أقل من السابق
- كما كان من قبل
- أعلى / أكثر من قبل
- لا ينطبق
- أفضل عدم الإجابة

الأنشطة البدنية للأطفال / ممارسة الرياضة

- أقل من السابق
- كما كان من قبل
- أعلى / أكثر من قبل
- لا ينطبق
- أفضل عدم الإجابة

انفعال الأطفال / غضبهم

- أقل من السابق
- كما كان من قبل
- أعلى / أكثر من قبل
- لا ينطبق
- أفضل عدم الإجابة

تواصل الأطفال مع الأصدقاء

- أقل من السابق
- كما كان من قبل
- أعلى / أكثر من قبل
- لا ينطبق
- أفضل عدم الإجابة

تواصل الأطفال مع أفراد الأسرة

- أقل من السابق
- كما كان من قبل
- أعلى / أكثر من قبل
- لا ينطبق
- أفضل عدم الإجابة

الحياة المدرسية للأطفال

- أقل من السابق
- كما كان من قبل
- أعلى / أكثر من قبل
- لا ينطبق
- أفضل عدم الإجابة

الصحة العقلية العامة للأطفال

- أقل من السابق
- كما كان من قبل
- أعلى / أكثر من قبل
- لا ينطبق
- أفضل عدم الإجابة

الصحة البدنية العامة للأطفال

○أقل من السابق
○كما كان من قبل
○أعلى / أكثر من قبل
○لا ينطبق
○أفضل عدم الإجابة

الصحة العاطفية العامة للأطفال(السعادة)
○أقل من السابق
○كما كان من قبل
○أعلى / أكثر من قبل
○لا ينطبق
○أفضل عدم الإجابة

28. مشاكل أخرى (رجاءاً، لا تتردد في الكتابة هنا)

يرجى الاتصال بنا إذا كنت ترغب في التشاور مباشرة مع أعضاء NPO
(البريد الإلكتروني: info@universalaid.jp؛ هاتف: 090-29003934).
نرغب في تقديم الدعم اللازم للمقيمين غير اليابانيين من أجل عيش حياة مريحة ، حتى في ظل فترة كوفيد-19 هذه.

شكرا لك مقدماً على تعاونك الطيب.

关于在日非本国籍居民在新冠肺炎期间的焦虑与生活问题的横断面调查

由 UNIVERSALAID.JP 携手长崎大学进行的本次调查，旨在了解在新冠肺炎流行期间，旅居日本的外国人（由其是长崎市）所面临的各种问题及焦虑，以期为这些问题及担忧提供相应的建议和咨询。

本次调查共包含 28 个问题，回答完需要约 7-10 分钟。您可以随时退出问卷调查。您在本次调查中提供的所有信息都是机密的，不会以任何方式泄露。此外，本次调查采取完全匿名的方式，无其它相关风险。

请通过点击下面链接或扫二维码的方式参与调查。
https://universalaid.jp/survey2020/

若您有任何关于本次调查的疑问或意见，请联系
UNIVERSALAID.JP 的 Yoshimi Matsuo 女士
邮件地址：info@universalaid.jp
联系电话：090-2900-3934
或长崎大学的 Nguyen Tien Huy 医生。
邮件地址：tienhuy@nagasaki-u.ac.jp

若完成并提交问卷，即表示您同意参与本次调查。

1. 您已年满十八周岁并同意参与本次研究调查？

○ 是
○ 否

第一部分：基本信息

2. 您的国籍

▢不想作答

3. 您的现居地

〇 长崎市

〇 长崎县的其它地区

〇 日本的其它县

〇 不想作答

4. 您的年龄

〇 18-24 岁

〇 25-34 岁

〇 35-44 岁

〇 45-54 岁

〇 55-64 岁

〇 65 岁及以上

〇 不想作答

5. 您的性别

〇 男性

〇 女性

〇 不想作答

〇 其他（请填写）

6. 您的婚姻状况

〇 单身，未婚

〇 已婚或同居

〇 丧偶

〇 离异

〇 分居

〇 不想作答

7. 您伴侣的国籍

○ 日本国籍
○ 和我的国籍一样
○ 我没有伴侣
○ 不想作答
○ 其他（请填写）

8. 您的职业（多选）

○ 全职工作
○ 兼职/临时工
○ 个体经营
○ 家庭主妇/家庭主夫
○ 待业
○ 学生
○ 退休
○ 不想作答
○ 其他（请填写）

9. 教育程度
○ 研究生及以上
○ 大学
○ 高中
○ 初中
○ 小学
○ 无正规教育
○ 不想作答

10. 签证类型
○ 入籍
○ 永居签证
○ 工作签证
○ 留学签证
○ 配偶签证
○ 短期签证（旅游，商务）
○ 不想作答
○ 其他（请填写）

11. 您的健康保险类型（单选或多选）

〇 国民健康保险
〇 社会保险
〇 其它个人健康保险
〇 海外旅行保险
〇 无
〇 不想作答

12. 您在日本多久了

〇 少于一年
〇 一到两年
〇 三到五年
〇 六到十年
〇 超过十年
〇 不想作答

13. 您的日语水平

〇 母语水平
〇 可以应对学习或工作场景
〇 生活中的交流没有问题
〇 我的日语不是很好
〇 我完全不会说日语
〇 不想作答

14. 您的家里住了几口人（包括您在内）

〇 1
〇 2
〇 3
〇 4
〇 5 及以上

15. 您家里 65 岁或以上老人的数目

- ○ 0
- ○ 1
- ○ 2
- ○ 3
- ○ 4
- ○ 5 及以上

16. 您别的家庭成员（父母、姐妹、兄弟等）的现居地（单选或多选）

- ○ 我没有别的家庭成员
- ○ 和我住在一起
- ○ 在日本别的地方居住
- ○ 在他们原本的国家居住
- ○ 在别的国家
- ○ 我不知道
- ○ 不想作答

17. 您上次见您的家庭成员（父母、姐妹、兄弟等）是什么时候

- ○ 少于一年
- ○ 一到两年
- ○ 三到五年
- ○ 六到十年
- ○ 超过十年
- ○ 不想作答

18. 您多久联系一次您的家庭成员（父母、姐妹、兄弟等）

- ○ 每天
- ○ 每周
- ○ 每月

○ 一年几次

○ 不记得了

○ 从不

○ 不想作答

第二部分：信息网络

19. 您有能与之谈论烦恼的人吗

○ 是

○ 否

○ 不想作答

20. 您一般和谁谈论您的烦恼（单选或多选）

○ 母亲

○ 父亲

○ 姐妹

○ 兄弟

○ 堂兄

○ 其他亲属

○ 老师

○ 同事

○ 在日本的朋友

○ 本国的朋友

○ 教会的牧师

○ 政府的咨询师

○ 不想作答

○ 其他（请填写）

21. 您一般从哪儿获取新冠肺炎的资讯（单选或多选）

○ 在国内的家人
○ 在日本的家人
○ 日本朋友
○ 同国籍的朋友
○ 一些组织和机构、同乡会、中国餐馆超市等
○ 县厅的告示
○ 健康手册或县厅的网站
○ 国际交流协会
○ 电视、报纸、杂志、网络
○ 社交网络（推特、Facebook、Instagram 等）
○ 我没有渠道获取信息
○ 不想作答
○ 其他（请填写）

22. 您想要获取什么样的中文资讯（单选或多选）

○ 当我有新冠肺炎相关症状时应该怎么做
○ 当我在新冠肺炎流行期间染上别的疾病或受伤时应该怎么做
○ 有灾难发生时该怎么做
○ 去医院或诊所时该怎么做
○ 与邻居有矛盾、遭遇家暴、遇到性骚扰时该怎么办
○ 生产和生育相关信息
○ 孩子的相关问题如何处理
○ 孩子的教育和学校资讯
○ 新冠肺炎的感染和控制情况
○ 新冠肺炎多处确诊的地方
○ 新冠肺炎的预防措施
○ 新冠肺炎相关的经济援助
○ 如何返回自己祖国
○ 怎样延期签证
○ 怎样从日本联系国内的家人
○ 公共交通系统的资讯
○ 怎样考驾照
○ 税收和养老金制度
○ 社区活动
○ 找房
○ 求职相关信息
○ 怎样购买国民健康保险或个人健康保险
○ 不想作答
○ 其他（请填写）

第三部分：最近遇到的问题

23. 您最近

有家庭成员感染新冠肺炎吗？

○ 是

○ 否

○ 不想作答

有家庭成员有新冠肺炎相关症状但没有途径进行核酸检测？

○ 是

○ 否

○ 不想作答

在学习或工作上遇到麻烦或困难？

○ 是

○ 否

○ 不想作答

因为外国人的身份受到歧视？

○ 是

○ 否

○ 不想作答

失去了工作？

○ 是

○ 否

○ 不想作答

遭遇了家庭暴力？

○ 是

○ 否

○ 不想作答

被性骚扰？

○ 是

○ 否

○ 不想作答

您最近有更多的宗教方面或精神层面的感受吗？

○ 是

○ 否

○ 不想作答

第四部分：个人生活上的改变

24.请描述您最近(或当下)的感受或心情

我感到心情平静

- ○ 完全没有
- ○ 有一些
- ○ 中等程度
- ○ 非常明显

我现在是紧绷的

- ○ 完全没有
- ○ 有一些
- ○ 中等程度
- ○ 非常明显

我感到心烦意乱

- ○ 完全没有
- ○ 有一些
- ○ 中等程度
- ○ 非常明显

我现在很放松

- ○ 完全没有
- ○ 有一些
- ○ 中等程度
- ○ 非常明显

我感到满足

- ○ 完全没有
- ○ 有一些
- ○ 中等程度
- ○ 非常明显

我很担心

- ○ 完全没有
- ○ 有一些
- ○ 中等程度
- ○ 非常明显

我感到寂寞

- ○ 完全没有
- ○ 有一些
- ○ 中等程度
- ○ 非常明显

我感到与世隔绝

- ○ 完全没有
- ○ 有一些
- ○ 中等程度
- ○ 非常明显

我害怕感染新冠肺炎

- ○ 完全没有
- ○ 有一些
- ○ 中等程度
- ○ 非常明显

25. 问题的程度

睡眠时长

- ○ 比之前少
- ○ 没有变化
- ○ 比之前多
- ○ 不适用
- ○ 不想作答

体重

- ○ 比之前少
- ○ 没有变化
- ○ 比之前多
- ○ 不适用
- ○ 不想作答

饮酒量

- ○ 比之前少
- ○ 没有变化
- ○ 比之前多
- ○ 不适用
- ○ 不想作答

吸烟量

- ○ 比之前少
- ○ 没有变化
- ○ 比之前多
- ○ 不适用
- ○ 不想作答

打电子游戏或者玩手机及看电视的时间

- ○ 比之前少
- ○ 没有变化
- ○ 比之前多
- ○ 不适用
- ○ 不想作答

运动或活动时间

- 〇 比之前少
- 〇 没有变化
- 〇 比之前多
- 〇 不适用
- 〇 不想作答

照顾孩子的时间

- 〇 比之前少
- 〇 没有变化
- 〇 比之前多
- 〇 不适用
- 〇 不想作答

家庭矛盾的次数

- 〇 比之前少
- 〇 没有变化
- 〇 比之前多
- 〇 不适用
- 〇 不想作答

与邻居或朋友的社交

- 〇 比之前少
- 〇 没有变化
- 〇 比之前多
- 〇 不适用
- 〇 不想作答

总体的精神状态

- 〇 比之前少
- 〇 没有变化
- 〇 比之前多
- 〇 不适用
- 〇 不想作答

总体的身体状态

- 〇 比之前少
- 〇 没有变化

- 〇 比之前多
- 〇 不适用
- 〇 不想作答

总体的心理建康状态(快乐程度)

- 〇 比之前少
- 〇 没有变化
- 〇 比之前多
- 〇 不适用
- 〇 不想作答

性生活的满意程度

- 〇 比之前少
- 〇 没有变化
- 〇 比之前多
- 〇 不适用
- 〇 不想作答

个人收入

- 〇 比之前少
- 〇 没有变化
- 〇 比之前多
- 〇 不适用
- 〇 不想作答

整个家庭的财务状况

- 〇 比之前少
- 〇 没有变化
- 〇 比之前多
- 〇 不适用
- 〇 不想作答

26. 与您一同生活的未成年孩子有几个

- 〇 0
- 〇 1
- 〇 2
- 〇 3

○ 4

○ 5 及以上

27. 您的孩子日常生活问题的程度

孩子的睡眠时长

- ○ 比之前少
- ○ 没有变化
- ○ 比之前多
- ○ 不适用
- ○ 不想作答

孩子打电子游戏或者玩手机及看电视的时间

- ○ 比之前少
- ○ 没有变化
- ○ 比之前多
- ○ 不适用
- ○ 不想作答

孩子运动或活动时间

- ○ 比之前少
- ○ 没有变化
- ○ 比之前多
- ○ 不适用
- ○ 不想作答

孩子闹脾气、生气

- ○ 比之前少
- ○ 没有变化
- ○ 比之前多
- ○ 不适用
- ○ 不想作答

孩子和朋友的交流时间

- ○ 比之前少
- ○ 没有变化
- ○ 比之前多
- ○ 不适用

- 不想作答

孩子和家庭成员的交流时间

- 比之前少
- 没有变化
- 比之前多
- 不适用
- 不想作答

孩子的校园生活

- 比之前少
- 没有变化
- 比之前多
- 不适用
- 不想作答

孩子的总体的精神状态

- 比之前差
- 没有变化
- 比之前好
- 不适用
- 不想作答

孩子的总体的身体状态

- 比之前差
- 没有变化
- 比之前好
- 不适用
- 不想作答

孩子的总体的心理建康状态(快乐程度)

- 比之前少
- 没有变化
- 比之前多
- 不适用
- 不想作答

28.您是否还有别的问题（请填写在下方）

如果您想直接与我们非营利组织的成员进行沟通、咨询，请联系我们。

邮件地址：info@universalaid.jp

联系电话：090-2900-3934

即使在新冠肺炎流行期间，我们也一直致力于给旅居日本的外国友人们提供创造安全舒适的生活环境的帮助。

非常感谢您参与本次调查。

Problems and Anxieties of non-Japanese residents living in Japan undergoing COVID-19 situation: A cross-sectional survey

We, UNIVERSALAID.JP and Nagasaki University, are conducting this survey to identify the problems and anxieties of non-Japanese residents living in Japan (particularly in Nagasaki City) caused by COVID-19 pandemic, in an effort to provide suggestions and consultations to heal such troubles and worries.

The survey comprises 28 questions and it may take approximately 7-10 minutes. You are free to decline or withdraw from participation at any time. The responses collected from this survey are confidential and will not be revealed under any condition. In addition, the survey will be completely anonymous and there are no risk associated.

Please participate in the survey by following URL or QR Code. https://universalaid.jp/survey2020/

For any further queries you can contact our team by emailing to Ms. Yoshimi Matsuo, UNIVERSALAID.JP (Email: info@universalaid.jp; Tel: 090-2900-3934) or Dr. Nguyen Tien Huy, Nagasaki University (Email: tienhuy@nagasaki-u.ac.jp).

By completing and submitting this survey, you are indicating your consent to participate in the survey.

1. Are you over 18 years old and do you agree to participate in this research project?

○ YES
○ NO

SECTION 1: GENERAL INFORMATIONS

2. What is your country of origin?

☐ Prefer not to answer

3. Where are you living?

◯ Nagasaki City
◯ Other cities of Nagasaki Prefecture
◯ Other prefectures
◯ Prefer not to answer

4. Your age range (years)

- ◯ 18-24 years old
- ◯ 25-34 years old
- ◯ 35-44 years old
- ◯ 45-54 years old
- ◯ 55-64 years old
- ◯ 65 years old or older
- ◯ Prefer not to answer

5. Gender

- ◯ Male
- ◯ Female
- ◯ Prefer not to answer
- ◯ Other (please specify)

6. Marital status

- ◯ Single, never married
- ◯ Married or domestic partnership
- ◯ Widowed
- ◯ Divorced
- ◯ Separated
- ◯ Prefer not to answer

7. What is the original nationality of your spouse/partner?

- ◯ Japanese
- ◯ Same nationality as myself
- ◯ I have no spouse/partner
- ◯ Prefer not to answer
- ◯ Others

8. Job/Occupation (select one or more)

- Full-time employee
- Part-time employee/casual employment
- Self-employed
- Housewife/husband
- Unemployed
- Student
- Retired
- Prefer not to answer
- Other (please specify)

9. Education

- Post-graduation or higher
- College/university degree
- Senior high school
- Junior high school
- Elementary/primary school
- No school
- Prefer not to answer

10. Immigration status

- ◯ Naturalized
- ◯ Permanent resident
- ◯ Work-permit
- ◯ Student visa
- ◯ Family dependent visa
- ◯ Temporary visa (tourists, business)
- ◯ Prefer not to answer
- ◯ Other (please specify)

11. What type of health insurance do you have? (select one or more)

- ◯ National Health Insurance
- ◯ Employees' Health Insurance
- ◯ Private Health Insurance
- ◯ Travel Health Insurance
- ◯ None
- ◯ Prefer not to answer

12. How long have you been staying in Japan?

- ◯ less than 1 year
- ◯ 1-2 year
- ◯ 3-5 years
- ◯ 6-10 years
- ◯ more than 10 years
- ◯ Prefer not to answer

13. What is your Japanese level?

- ◯ I can speak on the same level as Japanese people.
- ◯ I can speak well enough for work or study.
- ◯ I can speak well enough to have no trouble in everyday life.
- ◯ I can’t speak in Japanese very well.
- ◯ I can’t speak Japanese at all.
- ◯ Prefer not to answer.

14. The total number of people living in your home/room (including yourself).

▢ 1
▢ 2
▢ 3
▢ 4
▢ 5 or more

15. Number of adults aged 65 or above living with you.

▢ 0
▢ 1
▢ 2
▢ 3
▢ 4
▢ 5 or more

16. Where are other family members (parents, sisters, brothers) residing now? (select one or more)

▢ I don’t have other family members.
▢ With me in Japan.
▢ In other house in Japan
▢ Home country
▢ Other countries
▢ I don’t know.
▢ Prefer not to answer.

17. When was the last time you met your other family members (parents, sister, brothers)?

▢ Less than 1 year
▢ 1-2 year
▢ 3-5 years
▢ 6-10 years
▢ More than 10 years
▢ Prefer not to answer

18. How often do you call your family members (parents, sisters, brothers)?

▢ Every day
▢ Every week
▢ Every month
▢ Few times a year
▢ Can not recall
▢ Never
▢ Prefer not to answer

SECTION 2: INFORMATION NETWORK

19. Do you have anyone to share your problems with?

◯ Yes

◯ No

◯ Prefer not to answer

20. Who can you share your problems with? (select one or more)

▢ my mother
▢ my father
▢ my sister
▢ my brother
▢ my cousin
▢ other relatives
▢ teacher
▢ colleagues
▢ Japanese friend
▢ my home country' s friend
▢ religious leader
▢ Government consultant
▢ Prefer not to answer
▢ Other (please specify)

21. Where do you get the information about COVID-19? (select one or more)

▢ Family in your country
▢ Family in Japan
▢ Japanese friends
▢ Friends from the same country
▢ Organizations, facilities, or stores where people from the same country gather
▢ Municipal and prefectural office
▢ Public information magazines and websites of municipalities and prefectures
▢ International Exchange NPO
▢ TV, newspaper, magazines, or Internet
▢ SNS (Twitter, Facebook, Instagram, and so on)
▢ I don’t know how to get information
▢ Prefer not to answer
▢ Other (please specify)

22. What kind of information do you want in your language?

- ▢ What to do in case I have a suspected symptom of COVID-19
- ▢ What to do in case of illness or injuries other than COVID-19 in this COVID-19 period
- ▢ What to do in case of disaster
- ▢ What to do when going to hospitals/clinics
- ▢ What to do in case I have troubles with neighbors, domestic violence, or sexual harassment
- ▢ Delivery and childbearing
- ▢ What to do in case my kid has a problem
- ▢ Education, schools for children
- ▢ The infection-control situation of COVID-19
- ▢ Where the COVID-19 hotspots are
- ▢ Preventive measures against COVID-19
- ▢ Financial assistance related to COVID-19
- ▢ How to go back to the home country
- ▢ How to extend a visa
- ▢ How to call family from Japan
- ▢ Public transportations
- ▢ How to get a driving license
- ▢ The tax and pension system
- ▢ Community events
- ▢ Experiences looking for a house to live
- ▢ Job information and experiences looking for a job
- ▢ How to buy a national and/or private health insurance
- ▢ Prefer not to answer
- ▢ Other (please specify)

SECTION 3: SHORT QUESTIONS

23. Have you recently?

had a family member with confirmed COVID-19?

Yes No Prefer not to answer

had a family member with suspected COVID-19 infection but could not get a test?

Yes No Prefer not to answer

had troubles/difficulties with learning or working?

Yes No Prefer not to answer

felt discriminated for simply being a non-Japanese?

Yes No Prefer not to answer

lost your job?

Yes No Prefer not to answer

had domestic violence in your home?

Yes No Prefer not to answer

been sexually harassed?

Yes No Prefer not to answer

felt more spiritually/religiously activity?

Yes No Prefer not to answer

SECTION 4: CHANGES IN PERSONAL LIFE

24. Describe your recent (or current) feelings/moods?

I feel calm

◯Not at all ◯Somewhat ◯Moderately ◯Very much

I am tense

◯Not at all ◯Somewhat ◯Moderately ◯Very much

I feel upset

◯Not at all ◯Somewhat ◯Moderately ◯Very much

I am relaxed

◯Not at all ◯Somewhat ◯Moderately ◯Very much

I feel content

◯Not at all ◯Somewhat ◯Moderately ◯Very much

I am worried

◯Not at all ◯Somewhat ◯Moderately ◯Very much

I felt lonely

◯Not at all ◯Somewhat ◯Moderately ◯Very much

I felt isolated

◯Not at all ◯Somewhat ◯Moderately ◯Very much

I am afraid of getting COVID-19 infection

◯Not at all ◯Somewhat ◯Moderately ◯Very much

25. LEVEL OF YOUR PROBLEMS

Sleep duration

◯Less than before ◯Same as before ◯More than before ◯Not applicable ◯I prefer not to answer

Body weight

◯Less than before ◯Same as before ◯More than before ◯Not applicable ◯I prefer not to answer

Alcohol consumption

◯Less than before ◯Same as before ◯More than before ◯Not applicable ◯I prefer not to answer

Smoking

◯Less than before ◯Same as before ◯More than before ◯Not applicable ◯I prefer not to answer

Playing game/spending time on smartphone/TV

◯Less than before ◯Same as before ◯More than before ◯Not applicable ◯I prefer not to answer

Physical activities/doing exercise

◯Less than before ◯Same as before ◯More than before ◯Not applicable ◯I prefer not to answer

Duration of parenting or childcare

◯Less than before ◯Same as before ◯More than before ◯Not applicable ◯I prefer not to answer

Conflict/quarrel in your home

◯Less than before ◯Same as before ◯More than before ◯Not applicable ◯I prefer not to answer

Communication with neighbors or friends

◯Less than before ◯Same as before ◯More than before ◯Not applicable ◯I prefer not to answer

Your overall mental health

◯Less than before ◯Same as before ◯More than before ◯Not applicable ◯I prefer not to answer

Your overall physical health
◯Less than before ◯Same as before ◯More than before ◯Not applicable ◯I prefer not to answer

Your overall emotional health (happiness)
◯Less than before ◯Same as before ◯More than before ◯Not applicable ◯I prefer not to answer

Your overall sexual satisfaction
◯Less than before ◯Same as before ◯More than before ◯Not applicable ◯I prefer not to answer

Personal income
◯Less than before ◯Same as before ◯More than before ◯Not applicable ◯I prefer not to answer

Overall financial stability of your household
◯Less than before ◯Same as before ◯More than before ◯Not applicable ◯I prefer not to answer

26. Number of children under age 18 living with you.

- ○ 0
- ○ 1
- ○ 2
- ○ 3
- ○ 4
- ○ More than 4

27. LEVEL OF YOUR CHILDREN’S PROBLEMS

Children’ s sleep duration

Less than before Same as before More than before Not applicable I prefer not to answer

Children’ s playing game/spending time on smartphone/TV

Less than before Same as before More than before Not applicable I prefer not to answer

Children’ s physical activities/doing exercise

Less than before Same as before More than before Not applicable I prefer not to answer

Children’ s outburst/angriness

Less than before Same as before More than before Not applicable I prefer not to answer

Children’ s communication with friends

Less than before Same as before More than before Not applicable I prefer not to answer

Children’ s communication with family members

Less than before Same as before More than before Not applicable I prefer not to answer

Children’ s school life

Less than before Same as before More than before Not applicable I prefer not to answer

Children’ s overall mental health

Less than before Same as before More than before Not applicable I prefer not to answer

Children’ s overall physical health

Less than before Same as before More than before Not applicable I prefer not to answer

Children’ s overall emotional health happiness

◯Less than before ◯Same as before ◯More than before ◯Not applicable ◯I prefer not to answer

28. Others problems (please feel free to write here)

Please contact us if you wish to directly consult with our NPO members (Email: info@universalaid.jp; Tel: 090-2900-3934). We wish to provide the necessary support to non Japanese residents in order to lead a comfortable life, even under this COVID-19 period.

Thank you in advance for your kind collaborations.

Mga problema at mga ligalig ng mga residenteng hindi Hapones na naninirahan sa Bansang Hapon na sumasailalim sa sitwasyong COVID-19: Isang *cross-sectional survey*

Kami sa UNIVERSALAID.JP at Nagasaki University ay nagsasagawa ng pagsisiyasat patungkol sa mga problema at mga ligalig o mga rason ng pagkabalisa ng mga residenteng hindi Hapones na nakatira sa Bansang Hapon (lalong-lalo na sa lungsod ng Nagasaki) dulot ng pandemyang COVID-19, sa pagsisikap na makapagbigay ng mga mungkahi at konsultasyon para maibsan ang pagkaligalig at pag-aalala ng mga ito.

Ang pagsisiyasat na ito ay binubuo ng dalawampu't walong (28) tanong, na masasagutan sa humigit kumulang pito hanggang sampung (7-10) minuto. Ikaw ay pwedeng tumanggi o umurong sa pakikilahok anumang oras. Ang mga sagot na nakolekta sa pagsisiyasat na ito ay tinuturing na kumpidensyal at hindi malalantad sa kahit anumang kondisyon. Bilang dagdag, ang pagsusuri na ito ay ganap na walang lagda at walang panganib na nauugnay.

Maaaring lumahok po kayo sa pagsisiyasat na ito sa pamamagitan ng mga sumusunod na URL o QR code. https://universalaid.jp/survey2020/

Para sa anumang karagdagang mga katanungan, maaari kayong makipag-ugnayan sa aming grupo sa pamamagitan ng pag-email kay Bb. Yoshimi Matsuo, UNIVERSALAID.JP (Email: info@universalaid.jp; Tel: 090-2900-3934) o kay Dr. Nguyen Tien Huy, Nagasaki University (Email: tienhuy@nagasaki-u.ac.jp).

Sa pamamagitan ng pagkumpleto at pagsumite ng pagsisiyasat na ito, ipinapahiwatig mo ang iyong pahintulot na lumahok sa pagsisiyasat na ito.

1. Ikaw ba ay lampas labing walong (18) taong gulang at sumasang-ayon na lalahok sa proyekto ng pananaliksik na ito?
    - Oo
    - Hindi

SEKSYON 1: PANGKALAHATANG IMPORMASYON

2. Ano ang iyong pinagmulang bansa?

   - Mas gustong hindi sagutin

3. Saan ka nakatira?
   - Lungsod ng Nagasaki
   - Ibang lungsod sa Nagasaki Prefecture
   - Ibang Prefecture
   - Mas gustong hindi sagutin

4. Ano ang saklaw ng iyong edad (taon)?
   - 18-24 taong gulang
   - 25-34 taong gulang
   - 35-44 taong gulang
   - 45-54 taong gulang
   - 55-64 taong gulang
   - 65 taong gulang o higit pa
   - Mas gustong hindi sagutin

5. Kasarian
   - Lalaki
   - Babae
   - Mas gustong hindi sagutin
   - Iba pa (mangyaring tukuyin)

6. Estado ng kasal
   - Single, hindi nakapag-asawa
   - Kinasal o may kinakasama
   - Byudo o byuda
   - Dibursyado o dibursyada
   - Hiwalay sa asawa
   - Mas gustong hindi sagutin

7. Ano ang nasyonalidad ng iyong asawa/kinakasama?
   - Hapones
   - Parehas sa akin
   - Wala akong asawa/kinasakasama
   - Mas gustong hindi sagutin
   - Iba pa (mangyaring tukuyin)

8. Trabaho (pumili ng isa o higit pa)
   - Full-time na empleyado
   - Part-time na empleyado o kaswal na trabaho
   - Nagtatrabaho ng sarili
   - Maybahay/asawa
   - Walang trabaho
   - Mag-aaral
   - Nagretiro na
   - Mas gustong hindi sagutin
   - Iba pa (mangyaring tukuyin)

9. Edukasyon
   - *Post-graduation* o mas mataas pa
   - Kolehiyo/may degree sa unibersidad
   - *Senior High School*
   - *Junior High School*
   - Elementarya/pangunahing edukasyon
   - Hindi nakapag-aral
   - Mas gustong hindi sagutin

10. Estado ng imigrasyon
    - Naturalisasyon (pagtamo ng nasyonalidad ng Hapon)
    - Permanenteng residente
    - Permiso sa trabaho
    - Bisang pang-estudyante
    - Bisa na umaasa sa miyembro ng pamilya
    - Pansamantalang bisa (Turista, Negosyo)
    - Mas gustong hindi sagutin
    - Iba pa (mangyaring tukuyin)

11. Anong uri ng *health insurance* ang mayroon ka (pumili ng isa o higit pa) ?
    - Nasyonal na *Health Insurance*
    - Pang-empleyadong *Health Insurance*
    - Pribadong *Health Insurance*
    - Panglakbay na *Health Insurance*
    - Wala
    - Mas gustong hindi sagutin

12. Gaano katagal ka nang nananatili sa Bansang Hapon?
    - Hindi aabot ng 1 taon
    - 1-2 taon
    - 3-5 taon
    - 6-10 taon
    - Mahigit 10 taon
    - Mas gustong hindi sagutin

13. Nasa anong antas ng wikang Hapon ka na?
    - Ako ay nakasasalita kasabay ng mga Hapon.
    - Ako ay sapat na nakasasalita para sa trabaho o pag-aaral.
    - Ako ay sapat na nakasasalita para makaiwas sa problema sa pang-araw-araw na buhay.
    - Hindi ako masyadong nakasasalita ng wikang Hapon.
    - Hindi ako marunong magsalita ng wikang Hapon.
    - Mas gustong hindi sagutin

14. Kabuuang bilang ng mga taong nakatira sa inyong bahay o kwarto (kasama ang iyong sarili).
    - 1
    - 2
    - 3
    - 4
    - 5 o higit pa

15. Bilang ng matatanda na may edad 65 o higit pa na nakatira kasama mo.
    - 0
    - 1
    - 2
    - 3
    - 4
    - 5 o higit pa

16. Saan nakatira ang iyong mga kamag-anak (mga magulang, kapatid na babae, kapatid na lalaki) ngayon? (pumili ng isa o higit pa)
    - Wala akong ibang miyembro ng pamilya
    - Kasama ko sa bansang Hapon
    - Nasa ibang bahay sa bansang Hapon
    - Bansang pinagmulan
    - Iba pang mga bansa
    - Hindi ko alam.
    - Mas gustong hindi sagutin

17. Kailan ang huling pagkakataon na nagkita kayo ng iyong mga kamag-anak (mga magulang, kapatid na babae, kapatid na lalaki)?
    - Hindi aabot ng 1 taon
    - 1-2 taon
    - 3-5 taon
    - 6-10 taon
    - Mahigit 10 taon
    - Mas gustong hindi sagutin

18. Gaano mo kadalas tinatawagan ang iyong mga kamag-anak (mga magulang, kapatid na babae, kapatid na lalaki)?
    - Araw-araw
    - Kada-linggo
    - Kada-buwan
    - Kaunting beses sa isang taon
    - Hindi maalala
    - Hindi kailanman
    - Mas gustong hindi sagutin

SEKSYON 2: NETWORK NG IMPORMASYON

19. Mayroon ka bang taong mapagbabahagian ng iyong mga problema?
    - Oo
    - Hindi
    - Mas gustong hindi sagutin

20. Kanino mo ibinabahagi ang iyong mga problema? (pumili ng isa o higit pa)
    - Aking ina
    - Aking ama
    - Aking kapatid na babae
    - Aking kapatid na lalaki
    - Aking pinsan
    - Mga ibang kamag-anak
    - Guro
    - Mga kasamahan
    - Kaibigan na Hapon
    - Kaibigan sa bansang pinagmulan
    - Pinuno ng relihiyon
    - Mga kasangguni ng gobyerno
    - Mas gustong hindi sagutin
    - Iba pa (mangyaring tukuyin)

21. Saan ka nakakakuha ng impormasyon tungkol sa COVID-19? (pumili ng isa o higit pa)

- Pamilya sa sariling bansa
- Pamilya sa bansang Hapon
- Mga Hapones na kaibigan
- Mga kaibigan galing sa parehong bansa
- Mga organisasyon, mga pasilidad, o mga tindahan kung saan nagtitipon ang mga taong galing sa parehong bansa
- Mga munisipyo at *prefectural office*
- Mga magasin ng pampublikong impormasyon at *website* ng munisipyo at *prefecture*
- *International Exchange NPO* (*non-profit organization,* samahang hindi pangkalakalan)
- Telebisyon, pahayagan, magasin, o *internet*
- *SNS (Twitter, Facebook, Instagram*, at iba pa)
- Hindi ko alam paano makakakuha ng impormasyon.
- Mas gustong hindi sagutin
- Iba pa (mangyaring tukuyin)

22. Anong uri ng impormasyon ang nais mo na nasa iyong sariling wika? (pumili ng isa o higit pa)

- Ano ang gagawin kung sakaling mayroon akong hinihilaang sintomas ng COVID-19?
- Ano ang gagawin kung sakaling magkasakit o magkapinsala maliban sa COVID-19 sa panahon ng COVID-19?
- Ano ang gagawin kung sakaling magkasakuna?
- Ano ang gagawin kung pupunta ng ospital or *clinic*?
- Ano ang gagawin kung sakaling magkaproblema ako sa mga kapitbahay, karahasan sa tahanan, o sekswal na panliligalig
- Panganganak
- Ano ang gagawin kung sakaling may problema ang aking anak?
- Edukasyon, mga paaralan para sa mga bata
- Mga sitwasyon sa pagkontrol ng impeksyon ng COVID-19
- Kung nasaan ang COVID-19 *hotspots*
- Mga iwas-hakbang laban sa COVID-19
- Tulong-pinansyal kaugnay ng COVID-19
- Paano makakabalik sa sariling bansa
- Paano mapahaba ang bisa
- Paano tumawag sa pamilya mula sa Japan
- Mga pampublikong transportasyon
- Paano makakakuha ng lisensya sa pagmamaneho
- Ang sistema ng buwis at pensyon
- Kaganapan sa komunidad
- Mga karanasan sa paghahanap ng bahay na matitirhan
- Impormasyon sa trabaho at mga karanasan sa paghahanap ng trabaho
- Paano makabibili ng nasyonal o pribadong *health insurance*
- Mas gustong hindi sagutin
- Iba pa (mangyaring tukuyin)

SEKSYON 3: MAIKLING MGA TANONG

23. Nagkaroon ka ba kamakailan ng?

- kamag-anak na may kumpirmadong kaso ng COVID-19?
  - Oo
  - Hindi
  - Mas gustong hindi sagutin
- kamag-anak na may hinihilaang kaso ng COVID-19, ngunit hindi nakakuha ng pagsusuri?
  - Oo
  - Hindi
  - Mas gustong hindi sagutin
- problema o nahihirapan sa pag-aaral o trabaho?
  - Oo
  - Hindi
  - Mas gustong hindi sagutin
- pakiramdam ng diskriminasyon dahil lang sa hindi pagiging Hapon?
  - Oo
  - Hindi
  - Mas gustong hindi sagutin
- kawalan ng trabaho?
  - Oo
  - Hindi
  - Mas gustong hindi sagutin
- karahasan sa iyong sariling tahanan?
  - Oo
  - Hindi
  - Mas gustong hindi sagutin
- ligalig sekswal?
  - Oo
  - Hindi
  - Mas gustong hindi sagutin
- higit na dama pang-espiritwal/panrelihiyon?
  - Oo
  - Hindi
  - Mas gustong hindi sagutin

SEKSYON 4: MGA PAGBABAGO SA PERSONAL NA BUHAY

24. Ilarawan ang iyong kasalukuyang nararamdaman o kalooban.

- Kalmado ang aking pakiramdam.
    - Hindi sumasang-ayon
    - Medyo
    - Katamtaman
    - Labis na sumasang-ayon
- Ako ay tensyonado.
    - Hindi sumasang-ayon
    - Medyo
    - Katamtaman
    - Labis na sumasang-ayon
- Pakiramdam ko ay masama ang loob ko.
    - Hindi sumasang-ayon
    - Medyo
    - Katamtaman
    - Labis na sumasang-ayon
- Ako ay kalmado.
    - Hindi sumasang-ayon
    - Medyo
    - Katamtaman
    - Labis na sumasang-ayon

- Pakiramdam ko ay kontento na ako.
    - Hindi sumasang-ayon
    - Medyo
    - Katamtaman
    - Labis na sumasang-ayon

- Ako ay nag-aalala.
    - Hindi sumasang-ayon
    - Medyo
    - Katamtaman
    - Labis na sumasang-ayon

- Pakiramdam ko ay nag-iisa ako.
    - Hindi sumasang-ayon
    - Medyo
    - Katamtaman
    - Labis na sumasang-ayon

- Pakiramdam ko nakahiwalay ako.
    - Hindi sumasang-ayon
    - Medyo
    - Katamtaman
    - Labis na sumasang-ayon

- Ako ay natatakot na mahawaan ng impeksyong COVID-19.
    - Hindi sumasang-ayon
    - Medyo
    - Katamtaman
    - Labis na sumasang-ayon

25. Antas ng iyong mga problema

- Ang tagal ng pagtulog
    - Mas kaunti kaysa sa dati
    - Parehas ng dati
    - Higit pa sa dati
    - Hindi naaangkop
    - Mas gustong hindi sagutin
- Sariling timbang
    - Mas kaunti kaysa sa dati
    - Parehas ng dati
    - Higit pa sa dati
    - Hindi naaangkop
    - Mas gustong hindi sagutin

- Konsumo ng alkohol
    - Mas kaunti kaysa sa dati
    - Parehas ng dati
    - Higit pa sa dati
    - Hindi naaangkop

    - Mas gustong hindi sagutin

- Paninigarilyo
    - Mas kaunti kaysa sa dati
    - Parehas ng dati
    - Higit pa sa dati
    - Hindi naaangkop
    - Mas gustong hindi sagutin

- Paglalaro/paggugol ng oras sa smartphone o telebisyon
    - Mas kaunti kaysa sa dati
    - Parehas ng dati
    - Higit pa sa dati
    - Hindi naaangkop
    - Mas gustong hindi sagutin

- Mga pisikal na aktibidad/pag-eehersisyo
    - Mas kaunti kaysa sa dati
    - Parehas ng dati
    - Higit pa sa dati
    - Hindi naaangkop
    - Mas gustong hindi sagutin

- Oras sa pagiging magulang o pagbabantay sa bata
    - Mas kaunti kaysa sa dati
    - Parehas ng dati
    - Higit pa sa dati
    - Hindi naaangkop
    - Mas gustong hindi sagutin

- Hindi pagkakaintindihan/away sa bahay
    - Mas kaunti kaysa sa dati
    - Parehas ng dati
    - Higit pa sa dati
    - Hindi naaangkop
    - Mas gustong hindi sagutin

- Komunikasyon sa kapitbahay o mga kaibigan
    - Mas kaunti kaysa sa dati
    - Parehas ng dati
    - Higit pa sa dati
    - Hindi naaangkop
    - Mas gustong hindi sagutin

- Ang iyong pangkalahatang mental o sikolohikal na kalusugan
    - Mas kaunti kaysa sa dati
    - Parehas ng dati
    - Higit pa sa dati
    - Hindi naaangkop
    - Mas gustong hindi sagutin

- Ang iyong pangkalahatang pisikal na kalusugan o kalusugan ng pangangatawan
    - Mas kaunti kaysa sa dati
    - Parehas ng dati
    - Higit pa sa dati
    - Hindi naaangkop
    - Mas gustong hindi sagutin

- Ang iyong pangkalahatang emosyonal na kalusugan (kaligayahan)
    - Mas kaunti kaysa sa dati
    - Parehas ng dati
    - Higit pa sa dati
    - Hindi naaangkop
    - Mas gustong hindi sagutin

- Ang iyong pangkalahatang kasihayang pangsekswal
    - Mas kaunti kaysa sa dati
    - Parehas ng dati
    - Higit pa sa dati
    - Hindi naaangkop
    - Mas gustong hindi sagutin

- Personal na sweldo
    - Mas kaunti kaysa sa dati
    - Parehas ng dati
    - Higit pa sa dati
    - Hindi naaangkop
    - Mas gustong hindi sagutin

- Pangkalahatang katatagan ng inyong pananalapi ng pamilya
    - Mas kaunti kaysa sa dati
    - Parehas ng dati
    - Higit pa sa dati
    - Hindi naaangkop
    - Mas gustong hindi sagutin

26. Bilang ng mga batang wala pang 18 taong gulang na kasama sa bahay
    - 0
    - 1
    - 2
    - 3
    - 4
    - 5 o higit pa

27. Antas ng problema ng iyong mga anak
- Ang tagal ng pagtulog ng mga bata
    - Mas kaunti kaysa sa dati
    - Parehas ng dati
    - Higit pa sa dati
    - Hindi naaangkop
    - Mas gustong hindi sagutin
    -
- Oras sa paglalaro ng mga bata/paggugol ng oras sa *smartphone*/telebisyon
    - Mas kaunti kaysa sa dati
    - Parehas ng dati
    - Higit pa sa dati
    - Hindi naaangkop
    - Mas gustong hindi sagutin

- Mga pisikal na aktibidad ng mga bata/pag-eehersisyo
    - Mas kaunti kaysa sa dati
    - Parehas ng dati
    - Higit pa sa dati
    - Hindi naaangkop
    - Mas gustong hindi sagutin

- Ang biglang pagkagalit ng mga bata
    - Mas kaunti kaysa sa dati
    - Parehas ng dati
    - Higit pa sa dati
    - Hindi naaangkop
    - Mas gustong hindi sagutin

- Komunikasyon ng mga bata sa kanilang kaibigan
    - Mas kaunti kaysa sa dati
    - Parehas ng dati
    - Higit pa sa dati
    - Hindi naaangkop
    - Mas gustong hindi sagutin

- Komunikasyon ng mga bata sa mga kamag-anak
    - Mas kaunti kaysa sa dati
    - Parehas ng dati
    - Higit pa sa dati
    - Hindi naaangkop
    - Mas gustong hindi sagutin

- Buhay-eskwela ng mga bata
    - Mas kaunti kaysa sa dati
    - Parehas ng dati
    - Higit pa sa dati
    - Hindi naaangkop
    - Mas gustong hindi sagutin

- Ang pangkalahatang mental o sikolohikal na kalusugan ng mga bata
    - Mas kaunti kaysa sa dati
    - Parehas ng dati
    - Higit pa sa dati
    - Hindi naaangkop
    - Mas gustong hindi sagutin

- Ang pangkalahatang pisikal na kalusugan o kalusugan sa pangangatawan ng mga bata
    - Mas kaunti kaysa sa dati
    - Parehas ng dati
    - Higit pa sa dati
    - Hindi naaangkop
    - Mas gustong hindi sagutin

- Ang pangkalahatang emosyonal na kalusugan ng mga bata (kaligayahan)
    - Mas kaunti kaysa sa dati
    - Parehas ng dati
    - Higit pa sa dati
    - Hindi naaangkop
    - Mas gustong hindi sagutin

28. Iba pang mga problema (mangyaring huwag mag-atubiling sumulat dito)

Maaari po kayong makipag-ugnayan sa amin kung nais mong direktang kumonsulta sa aming mga miyembro ng NPO (Email: info@universalaid.jp; Tel: 090-2900-3934). Nais naming magbigay ng mga kinakailangang suporta sa mga residenteng hindi Hapones para mabuhay ng komportable kahit na nasa panahon ng COVID-19.

Maraming salamat sa inyong pakikipagtulungan.

Permasalahan dan kecemasan yang dihadapi penduduk non-Jepang di Jepang yang mengalami situasi COVID-19: survei *cross-sectional*

Kami, UNIVERSALAID.JP dan Nagasaki University, melakukan survei ini untuk mengidentifikasi masalah dan kecemasan warga non-Jepang yang tinggal di Jepang (khususnya di Kota Nagasaki) akibat pandemi COVID-19, dalam upaya memberikan saran dan konsultasi untuk mengatasi masalah dan kekhawatiran terkait.

Survei ini terdiri dari 28 pertanyaan dan membutuhkan waktu sekitar 7-10 menit. Anda dapat menolak atau menarik diri dari partisipasi kapan saja. Tanggapan yang dikumpulkan dari survei ini bersifat rahasia dan tidak akan diungkapkan dalam kondisi apa pun. Selain itu, survei akan sepenuhnya anonim dan tidak ada risiko yang ditimbulkan.

Mohon partisipasi Anda dalam survei ini melalui URL atau QR code. https://universalaid.jp/survey2020/

Untuk pertanyaan lebih lanjut, Anda dapat menghubungi tim kami dengan mengirimkan email pada Ms. Yoshimi Matsuo, UNIVERSALAID.JP (Email: info@universalaid.jp; Tel: 090-2900-3934) atau Dr. Nguyen Tien Huy, Nagasaki University (Email: tienhuy@nagasaki-u.ac.jp).

Dengan menyelesaikan dan mengirimkan survei ini, Anda telah menyetujui untuk berpartisipasi dalam survei.

1. Apakah Anda berusia lebih dari 18 tahun dan setuju untuk berpartisipasi dalam proyek ini?

- YA
- TIDAK

BAGIAN 1: INFORMASI UMUM

2. Dari negara mana Anda berasal?

- Memilih untuk tidak menjawab

3. Di mana Anda tinggal?

- Kota Nagasaki
- Kota lain di Perfektur Nagasaki
- Perfektur lainnya
- Memilih untuk tidak menjawab

4. Rentang usia Anda (tahun)

- 18-24 tahun
- 25-34 tahun
- 35-44 tahun
- 45-54 tahun
- 55-64 tahun
- 65 tahun atau diatas 65 tahun
- Memilih untuk tidak menjawab

5. Jenis kelamin

- Pria
- Wanita
- Memilih untuk tidak menjawab
- Lainnya (harap spesifikasikan)

6. Status pernikahan

- Lajang, belum pernah menikah
- Menikah atau pasangan tanpa ikatan pernikahan
- Duda/janda
- Bercerai
- Terpisah
- Memilih untuk tidak menjawab

7. Apakah asal kewarganegaraan dari pasangan Anda?

- Jepang
- Sama seperti saya
- Saya tidak memiliki pasangan
- Memilih untuk tidak menjawab
- Lainnya (harap spesifikasikan)

8. Pekerjaan (pilih salah satu atau lebih)

- Karyawan
- Karyawan paruh-waktu/pekerja lepas
- Bekerja sendiri
- Ibu/bapak rumah tangga
- Tidak bekerja
- Pelajar
- Pensiun
- Memilih untuk tidak menjawab
- Lainnya (harap spesifikasikan)

9. Pendidikan

- Pasca sarjana atau lebih tinggi
- Sarjana
- Sekolah Menengah Atas (SMA)
- Sekolah Menengah Pertama (SMP)
- Sekolah Dasar (SD)
- Tidak sekolah
- Memilih untuk tidak menjawab

10. Status imigrasi

- Naturalisasi
- Penduduk tetap
- Izin kerja
- Visa pelajar
- Visa tanggungan keluarga
- Visa sementara (turis, bisnis)
- Memilih untuk tidak menjawab
- Lainnya (harap spesifikasikan)

11.Jenis asuransi apa yang Anda miliki (pilih salah satu atau lebih)?

- Asuransi kesehatan nasional
- Asuransi kesehatan karyawan
- Asuransi kesehatan pribadi
- Asuransi kesehatan perjalanan
- Tidak ada
- Memilih untuk tidak menjawab

12. Sudah berapa lama Anda tinggal di Jepang?

- Kurang dari 1 tahun
- 1-2 tahun
- 3-5 tahun
- 6-10 tahun
- Lebih dari 10 tahun
- Memilih untuk tidak menjawab

13. Bagaimana kemampuan bahasa Jepang Anda?

- Saya dapat berbicara pada level yang sama dengan orang Jepang
- Saya dapat berbicara cukup baik untuk bekerja atau belajar
- Saya dapat berbicara cukup baik sehingga tidak mengalami kesulitan dalam hidup sehari-hari
- Saya tidak bisa berbicara dalam bahasa Jepang dengan baik.
- Saya tidak bisa bahasa Jepang sama sekali.
- Memilih untuk tidak menjawab

14. Jumlah total orang yang tinggal di rumah Anda (termasuk anda sendiri)
- 1
- 2
- 3
- 4
- 5 atau lebih

15. Jumlah orang dewasa berusia 65 atau lebih yang tinggal bersama Anda.
- 0
- 1
- 2
- 3
- 4
- 5 atau lebih

16. Di manakah anggota keluarga Anda yang lain (orang tua, saudara perempuan, saudara laki-laki) tinggal sekarang? (pilih satu atau lebih)

- Saya tidak punya anggota keluarga lain.
- Bersama saya di Jepang.
- Di rumah lain di Jepang
- Di negara asal
- Di negara lain
- Tidak tahu.
- Memilih untuk tidak menjawab

17. Kapan terakhir kali Anda bertemu dengan anggota keluarga lainnya (orangtua, saudara perempuan, saudara laki-laki)?

- Kurang dari 1 tahun
- 1-2 tahun
- 3-5 tahun
- 6-10 tahun
- Lebih dari 10 tahun
- Memilih untuk tidak menjawab

18. Seberapa sering Anda menghubungi anggota keluarga Anda (orangtua, saudara perempuan, saudara laki-laki)?

- Setiap hari
- Setiap minggu
- Setiap bulan
- Beberapa kali setahun
- Tidak ingat
- Tidak pernah
- Memilih untuk tidak menjawab

BAGIAN 2: INFORMASI JARINGAN

19. Apakah Anda memiliki seseorang untuk menceritakan masalah Anda?
    - YA
    - TIDAK
    - Memilih untuk tidak menjawab

20. Dengan siapa Anda dapat menceritakan masalah (pilih salah satu atau lebih)?
    - Ibu
    - Ayah
    - Saudara perempan
    - Saudara laki-laki
    - Sepupu
    - Kerabat lainnya
    - Guru
    - Kolega
    - Teman Jepang
    - Teman dari negara asal
    - Pemimpin keagamaan
    - Konsultan pemerintahan
    - Memilih untuk tidak menjawab
    - Lainnya (harap spesifikasikan)

21. Dari mana Anda mendapatkan informasi tentang COVID-19 (pilih salah satu atau lebih)?
    - Keluarga di negara Anda
    - Keluarga di Jepang
    - Teman Anda yang orang Jepang
    - Teman dari negara yang sama
    - Organisasi, fasilitas umum, atau toko tempat orang-orang dari negara yang sama berkumpul
    - Kantor kota dan prefektur
    - Majalah informasi publik dan situs web kota dan prefektur
    - Organisasi non-profit pertukaran internasional
    - TV, koran, majalah, atau Internet
    - Media sosial (Twitter, Facebook, Instagram, dan sebagainya)
    - Saya tidak tahu bagaimana cara untuk mendapatkan informasi
    - Memilih untuk tidak menjawab
    - Lainnya (harap spesifikasikan)

22. Jenis informasi apa yang Anda inginkan tersedia dalam bahasa Anda (pilih salah satu atau lebih)?

- Apa yang harus dilakukan jika saya memiliki dugaan gejala COVID-19
- Apa yang harus dilakukan jika terjadi sakit atau cedera selain COVID-19 dalam masa COVID-19 ini
- Apa yang harus dilakukan jika terjadi bencana
- Apa yang harus dilakukan saat pergi ke rumah sakit/klinik
- Apa yang harus saya lakukan jika saya memiliki masalah dengan tetangga, kekerasan dalam rumah tangga, atau pelecehan seksual
- Prosedur melahirkan anak
- Apa yang harus dilakukan jika anak saya memiliki masalah
- Pendidikan, sekolah untuk anak
- Situasi pengendalian infeksi COVID-19
- Di mana hotspot COVID-19 berada
- Tindakan pencegahan terhadap COVID-19
- Bantuan keuangan terkait COVID-19
- Cara kembali ke negara asal
- Cara memperpanjang visa
- Bagaimana cara menghubungi keluarga Anda dari Jepang
- Transportasi umum
- Cara mendapatkan Surat Ijin Mengemudi (SIM)
- Sistem pajak dan pensiun
- Acara komunitas
- Pengalaman mencari tempat tinggal
- Informasi pekerjaan dan pengalaman mencari pekerjaan
- Bagaimana cara membeli asuransi kesehatan nasional dan/atau swasta
- Memilih untuk tidak menjawab
- Lainnya (harap spesifikasikan)

BAGIAN 3: PERTANYAAN SINGKAT

23. Apakah Anda baru-baru ini?

- Memiliki anggota keluarga yang terkonfirmasi COVID-19?
  - ○ YA
  - ○ TIDAK
  - ○ Memilih untuk tidak menjawab

- Memiliki anggota keluarga yang diduga terinfeksi COVID-19 tetapi tidak bisa menjalani tes?
  - ○ YA
  - ○ TIDAK
  - ○ Memilih untuk tidak menjawab

- Mengalami masalah/kesulitan belajar atau bekerja?
  - ○ YA
  - ○ TIDAK
  - ○ Memilih untuk tidak menjawab
- Mendapatkan diskriminasi karena merupakan warga non-Jepang?
  - ○ YA
  - ○ TIDAK
  - ○ Memilih untuk tidak menjawab

- Kehilangan pekerjaan?
  - ○ YA
  - ○ TIDAK
  - ○ Memilih untuk tidak menjawab

- Mengalami kekerasan dalam rumah tangga?
  - ○ YA
  - ○ TIDAK
  - ○ Memilih untuk tidak menjawab

- Mengalami pelecehan seksual?
  - ○ YA
  - ○ TIDAK
  - ○ Memilih untuk tidak menjawab

- Menjadi lebih spiritual/aktif menjalani aktivitas keagamaan?
  - ○ YA
  - ○ TIDAK
  - ○ Memilih untuk tidak menjawab

BAGIAN 4: PERUBAHAN DALAM KEHIDUPAN PRIBADI

24. Jelaskan perasaan/suasana hati Anda saat ini

- Saya merasa tenang
  - ◯ Tidak tenang
  - ◯ Sedikit
  - ◯ Cukup
  - ◯ Sangat tenang

- Saya tegang
  - ◯ Tidak tenang
  - ◯ Sedikit
  - ◯ Cukup
  - ◯ Sangat tenang

- Saya kesal
  - ◯ Tidak tenang
  - ◯ Sedikit
  - ◯ Cukup
  - ◯ Sangat tenang

- Saya santai
  - ◯ Tidak tenang
  - ◯ Sedikit
  - ◯ Cukup
  - ◯ Sangat tenang

- Saya puas
  - ◯ Tidak tenang
  - ◯ Sedikit
  - ◯ Cukup
  - ◯ Sangat tenang

- Saya khawatir
  - ◯ Tidak tenang
  - ◯ Sedikit
  - ◯ Cukup
  - ◯ Sangat tenang

- Saya kesepian
  - ◯ Tidak tenang
  - ◯ Sedikit
  - ◯ Cukup
  - ◯ Sangat tenang

- Saya terisolasi
  - ○ Tidak tenang
  - ○ Sedikit
  - ○ Cukup
  - ○ Sangat tenang

- Saya takut terinfeksi COVID-19
  - ○ Tidak tenang
  - ○ Sedikit
  - ○ Cukup
  - ○ Sangat tenang

25. TINGKAT MASALAH ANDA

- Durasi (lama) tidur
  - ○ Lebih sedikit dari sebelumnya
  - ○ Sama seperti sebelumnya
  - ○ Lebih lama dari sebelumnya
  - ○ Tidak dapat ditentukan
  - ○ Memilih untuk tidak menjawab

- Berat badan
  - ○ Lebih sedikit dari sebelumnya
  - ○ Sama seperti sebelumnya
  - ○ Lebih lama dari sebelumnya
  - ○ Tidak dapat ditentukan
  - ○ Memilih untuk tidak menjawab

- Konsumsi alkohol
  - ○ Lebih sedikit dari sebelumnya
  - ○ Sama seperti sebelumnya
  - ○ Lebih lama dari sebelumnya
  - ○ Tidak dapat ditentukan
  - ○ Memilih untuk tidak menjawab

- Merokok
  - ○ Lebih sedikit dari sebelumnya
  - ○ Sama seperti sebelumnya
  - ○ Lebih lama dari sebelumnya
  - ○ Tidak dapat ditentukan
  - ○ Memilih untuk tidak menjawab

- Memainkan *game*/menghabiskan waktu menggunakan *smartphone*/TV

- ○ Lebih sedikit dari sebelumnya
- ○ Sama seperti sebelumnya
- ○ Lebih lama dari sebelumnya
- ○ Tidak dapat ditentukan
- ○ Memilih untuk tidak menjawab

- Aktivitas fisik/olah raga
  - ○ Lebih sedikit dari sebelumnya
  - ○ Sama seperti sebelumnya
  - ○ Lebih lama dari sebelumnya
  - ○ Tidak dapat ditentukan
  - ○ Memilih untuk tidak menjawab

- Durasi waktu pengasuhan anak
  - ○ Lebih sedikit dari sebelumnya
  - ○ Sama seperti sebelumnya
  - ○ Lebih lama dari sebelumnya
  - ○ Tidak dapat ditentukan
  - ○ Memilih untuk tidak menjawab

- Konflik/pertengkaran di rumah Anda
  - ○ Lebih sedikit dari sebelumnya
  - ○ Sama seperti sebelumnya
  - ○ Lebih lama dari sebelumnya
  - ○ Tidak dapat ditentukan
  - ○ Memilih untuk tidak menjawab

- Komunikasi dengan tetangga atau teman
  - ○ Lebih sedikit dari sebelumnya
  - ○ Sama seperti sebelumnya
  - ○ Lebih lama dari sebelumnya
  - ○ Tidak dapat ditentukan
  - ○ Memilih untuk tidak menjawab

- Kesehatan mental Anda secara keseluruhan
  - ○ Lebih sedikit dari sebelumnya
  - ○ Sama seperti sebelumnya
  - ○ Lebih lama dari sebelumnya
  - ○ Tidak dapat ditentukan
  - ○ Memilih untuk tidak menjawab

- Kesehatan fisik Anda secara keseluruhan
  - ○ Lebih sedikit dari sebelumnya
  - ○ Sama seperti sebelumnya
  - ○ Lebih lama dari sebelumnya

- ○ Tidak dapat ditentukan
- ○ Memilih untuk tidak menjawab

- Kesehatan emosional Anda secara keseluruhan (kebahagiaan)
  - ○ Lebih sedikit dari sebelumnya
  - ○ Sama seperti sebelumnya
  - ○ Lebih lama dari sebelumnya
  - ○ Tidak dapat ditentukan
  - ○ Memilih untuk tidak menjawab

- Kepuasan seksual Anda secara keseluruhan
  - ○ Lebih sedikit dari sebelumnya
  - ○ Sama seperti sebelumnya
  - ○ Lebih lama dari sebelumnya
  - ○ Tidak dapat ditentukan
  - ○ Memilih untuk tidak menjawab

- Penghasilan pribadi
  - ○ Lebih sedikit dari sebelumnya
  - ○ Sama seperti sebelumnya
  - ○ Lebih lama dari sebelumnya
  - ○ Tidak dapat ditentukan
  - ○ Memilih untuk tidak menjawab

- Kestabilan keuangan rumah tangga Anda secara keseluruhan
  - ○ Lebih sedikit dari sebelumnya
  - ○ Sama seperti sebelumnya
  - ○ Lebih lama dari sebelumnya
  - ○ Tidak dapat ditentukan
  - ○ Memilih untuk tidak menjawab

26. Jumlah anak dibawah usia 18 tahun yang tinggal bersama Anda
    - 0
    - 1
    - 2
    - 3
    - 4
    - 5 atau lebih

27. TINGKAT MASALAH PADA ANAK
    - Durasi tidur anak-anak
      - ○ Lebih sedikit/kurang dari sebelumnya
      - ○ Sama seperti sebelumnya
      - ○ Lebih lama dari sebelumnya
      - ○ Tidak dapat ditentukan

- ○ Memilih untuk tidak menjawab

- Anak-anak bermain *game*/menghabiskan waktu di *smartphone*/TV
  - ○ Lebih sedikit/kurang dari sebelumnya
  - ○ Sama seperti sebelumnya
  - ○ Lebih lama dari sebelumnya
  - ○ Tidak dapat ditentukan
  - ○ Memilih untuk tidak menjawab

- Aktivitas fisik anak/olahraga
  - ○ Lebih sedikit/kurang dari sebelumnya
  - ○ Sama seperti sebelumnya
  - ○ Lebih lama dari sebelumnya
  - ○ Tidak dapat ditentukan
  - ○ Memilih untuk tidak menjawab

- Anak-anak emosinya meledak-ledak/marah
  - ○ Lebih sedikit/kurang dari sebelumnya
  - ○ Sama seperti sebelumnya
  - ○ Lebih lama dari sebelumnya
  - ○ Tidak dapat ditentukan
  - ○ Memilih untuk tidak menjawab

- Komunikasi anak dengan teman-temannya
  - ○ Lebih sedikit/kurang dari sebelumnya
  - ○ Sama seperti sebelumnya
  - ○ Lebih lama dari sebelumnya
  - ○ Tidak dapat ditentukan
  - ○ Memilih untuk tidak menjawab

- Komunikasi anak dengan anggota keluarga
  - ○ Lebih sedikit/kurang dari sebelumnya
  - ○ Sama seperti sebelumnya
  - ○ Lebih lama dari sebelumnya
  - ○ Tidak dapat ditentukan
  - ○ Memilih untuk tidak menjawab

- Kehidupan sekolah anak-anak
  - ○ Lebih sedikit/kurang dari sebelumnya
  - ○ Sama seperti sebelumnya
  - ○ Lebih lama dari sebelumnya
  - ○ Tidak dapat ditentukan
  - ○ Memilih untuk tidak menjawab

- Kesehatan mental anak-anak secara keseluruhan

- ○ Lebih sedikit/kurang dari sebelumnya
- ○ Sama seperti sebelumnya
- ○ Lebih lama dari sebelumnya
- ○ Tidak dapat ditentukan
- ○ Memilih untuk tidak menjawab

▪ Kesehatan fisik anak-anak secara keseluruhan

- ○ Lebih sedikit/kurang dari sebelumnya
- ○ Sama seperti sebelumnya
- ○ Lebih lama dari sebelumnya
- ○ Tidak dapat ditentukan
- ○ Memilih untuk tidak menjawab

▪ Kebahagiaan/kesehatan emosional anak-anak secara keseluruhan

- ○ Lebih sedikit/kurang dari sebelumnya
- ○ Sama seperti sebelumnya
- ○ Lebih lama dari sebelumnya
- ○ Tidak dapat ditentukan
- ○ Memilih untuk tidak menjawab

28. Masalah lainnya (silakan tulis di sini)

Silakan hubungi kami jika Anda ingin berkonsultasi langsung dengan anggota organisasi non-profit kami (Email: info@universalaid.jp; Tel: 090-2900-3934).

Kami ingin memberikan dukungan yang diperlukan kepada penduduk non-Jepang untuk menjalani hidup yang nyaman, meskipun di bawah periode COVID-19 ini.

Terima kasih atas partisipasi Anda.

新型コロナウイルス感染拡大下で在日外国人が抱える問題や心配事に関するアンケート

私たち、UNIVERSALAID.JP と長崎大学は、在日外国人が新型コロナウイルス感染拡大により抱える問題や心配事を明らかにするため、このアンケートを実施しています。アンケート結果をもとに、これらの問題や心配事を和らげるための提案や相談などを行う予定です。

このアンケートには、28 問の質問があり、所要時間は約 7～10 分です。アンケートへの参加は自由で、参加を断ってもアンケートを途中でやめても構いません。このアンケートで集められた回答は、大切に扱われ公表されることはありません。また、このアンケートでは、全て匿名で個人が特定されることもありません。

下記 URL もしくは QR コードより、アンケートへの参加をお願いします。
https://universalaid.jp/survey2020/

このアンケートに関するお問合せは、下記までお願いします。

UNIVERSALAID.JP　代表　松尾　(Email: info@universalaid.jp; Tel: 090-2900-3934)
長崎大学　Dr. Nguyen Tien Huy　(Email: tienhuy@nagasaki-u.ac.jp)

なお、アンケートに最後まで回答し、提出することで、このアンケートへの参加に同意したものとみなされます。

１．あなたは 18 歳以上で、このアンケートへの参加に同意しますか？

〇はい
〇いいえ

セクション１：一般情報

2.　あなたの国籍はどこですか？

〇答えたくない

3.　あなたはどこに住んでいますか？
〇長崎市内
〇長崎市以外の長崎県内の市町村
〇長崎県以外の他県
〇答えたくない

4.　あなたの年齢層を教えてください。

〇 18 歳以上-24 歳以下
〇 25 歳以上-34 歳以下
〇 35 歳以上-44 歳以下
〇 45 歳以上-54 歳以下
〇 55 歳以上-64 歳以下
〇 65 歳以上
〇 答えたくない

5.　あなたの性別を教えてください。

〇男性
〇女性
〇答えたくない
〇その他（上記以外の場合は、以下に記入してください。）

6. あなたの婚姻状況を教えてください。

◯ 独身で結婚したことはありません。
◯ 配偶者（夫または妻）、またはパートナーがいます。
◯ **配偶者（夫または妻）が亡くなり**未亡人です。
◯ 離婚して、現在は独身です。
◯ 別居していますが、配偶者（夫または妻）がいます。
◯ 答えたくない

7. あなたの配偶者（夫または妻）／パートナーの国籍を教えてください。

◯ 日本人
◯ 私と同じ国籍です。
◯ 配偶者／パートナーはいません。
◯ 答えたくない
◯ その他（上記以外の場合は、以下に記入してください。）

8. あなたの雇用形態・職業を教えてください。（複数のオプションを選択できます。）

◯ フルタイム雇用
◯ パートタイム雇用／臨時雇用
◯ 自営業
◯ 主婦／主夫
◯ 無職
◯ 学生
◯ 定年退職者
◯ 答えたくない
◯ その他（上記以外の場合は、以下に記入してください。）

9. あなたの最終学歴を教えてください。

◯ 大学院卒業またはそれ以上
◯ 短大／大学卒業
◯ 高等学校卒業
◯ 中学校卒業
◯ 小学校卒業
◯ 学校教育を受けたことがない
◯ 答えたくない

10. あなたの在留資格を教えてください。

◯ 日本国籍を取得して帰化
◯ 永住者
◯ 就労ビザ
◯ 学生ビザ
◯ 家族滞在ビザ
◯ 短期滞在ビザ（観光・商用）
◯ 答えたくない
◯ その他（上記以外の場合は、以下に記入してください）

11. あなたが加入している健康保険について教えてください。（複数のオプションを選択できます。）

◯国民健康保険（**社会保険などの健康保険に加入していない人が加入する保険**）
◯社会保険 （**正社員や一定の条件を満たす非正規社員が加入する保険**）
◯民間の医療保険
◯ 海外旅行医療保険
◯ 未加入
◯ 答えたくない

12. あなたの日本滞在期間を教えてください。

◯1 年未満
◯1－2 年
◯3－5 年
◯6－10 年
◯11 年またはそれ以上
◯答えたくない

13. あなたの日本語のレベルを教えてください。

◯日本人と同じレベルで話せる
◯仕事または勉強に差し支えのないレベルで話せる
◯日常生活に差し支えのないレベルで話せる
◯あまり話せない
◯全く話せない
◯答えたくない

14. 一緒に暮らしている人数を教えてください（あなた自身も含めた人数を答えてください）。

▢ 1 人
▢ 2 人
▢ 3 人
▢ 4 人
▢ 5 人またはそれ以上

15.一緒に暮らしている 65 歳以上の高齢者の人数を教えてください。

▢ 0 人
▢ 1 人
▢ 2 人
▢ 3 人
▢ 4 人
▢ 5 人またはそれ以上

16. 現在、あなたの家族（両親・兄弟・姉妹）はどこに住んでいますか？（複数のオプションを選択できます。）

▢ その他の家族はいません。
▢ 日本で私と一緒に暮らしています。
▢ 日本に住んでいますが、一緒に暮らしていません。
▢ 母国に住んでいます。
▢ 日本・母国以外の国に住んでいます。
▢ 分かりません。
▢ 答えたくない

17. 家族（両親・兄弟・姉妹）と最後に会ったのはいつですか？

▢ 1 年以内
▢ 1－2 年前
▢ 3－5 年前
▢ 6－10 年前
▢ 11 年以上前
▢ 答えたくない

18. 家族（両親・兄弟・姉妹）とどのくらいの頻度で会話しますか？

▢ 毎日
▢ 毎週
▢ 毎月
▢ 年に数回
▢ 覚えていない
▢ 全くしない
▢ 答えたくない

SECTION 2:情報ネットワークについて

19. 困ったときに相談できる人はいますか？

◯ はい
◯ いいえ
◯ 答えたくない

20. 困ったときに誰に相談しますか？（複数のオプションを選択できます。）

▢ 自分の母親
▢ 自分の父親
▢ 自分の姉妹
▢ 自分の兄弟
▢ 自分のいとこ
▢ その他の親戚・親族
▢ **学校の**先生
▢ **職場の**同僚
▢ 日本人の友人
▢ 母国の友人
▢ 宗教の指導者
▢ 行政機関
▢ 答えたくない
▢ その他（上記以外の場合は、以下に記入してください。）

21. 新型コロナウイルスに関する情報をどこから入手していますか？（複数のオプションを選択できます。）

▢ 母国の家族
▢ 日本にいる家族
▢ 日本人の友人
▢ 母国の友人
▢ **同じ**母国出身の人の集まり（団体・施設・店など）
▢ 市役所や県庁
▢ 市町村や県の情報誌・ウェブサイト
▢ 国際交流団体
▢ テレビ・新聞・雑誌・インターネット

▢ SNS (Twitter, Facebook, Instagram 等)
▢ どのように入手できるか分かりません。
▢ 答えたくない
▢ その他（上記以外の場合は、以下に記入してください。）

22. どのような情報が母国語で欲しいですか？（複数のオプションを選択できます。）

▢ 新型コロナウイルス感染症と似た症状がある場合に取るべき行動
▢ 新型コロナウイルス感染拡大下で、新型コロナウイルス感染症以外の病気やケガをした場合に取るべき行動
▢ 災害が発生した場合に取るべき行動
▢ 病院やクリニック等の医療機関を受診する際に取るべき行動
▢ 近隣住民とのトラブル・家庭内暴力（DV）・セクハラがあった時に取るべき行動
▢ 出産関連
▢ 子どもの問題への対処方法
▢ 子どもの学校や教育
▢ 新型コロナウイルスの感染制御の状況
▢ 新型コロナウイルスの感染拡大地域（ホットスポット）
▢ 新型コロナウイルスの予防法
▢ 新型コロナウイルス感染拡大下での経済的支援
▢ 帰国方法
▢ ビザ延長手続き
▢ 日本から家族へ電話する方法
▢ 公共交通機関
▢ 運転免許取得
▢ 税金や年金制度
▢ 地域イベント
▢ 住む家を探す方法・体験談など
▢ 就職情報や就職活動の体験談など
▢ 国民健康保険や民間の医療保険の加入方法
▢ 答えたくない
▢ その他（上記以外の場合は、以下に記入してください。）

SECTION 3: 最近の状況に関する質問

23. あなたの最近の状況についておうかがいします。

新型コロナウイルスに感染した家族はいますか ？
◯はい ◯いいえ ◯ 答えたくない

新型コロナウイルス感染の疑いがあるが検査を受けられなかった家族はいますか？
◯はい ◯いいえ ◯ 答えたくない

学校や仕事で問題を抱えていますか？
◯はい ◯いいえ ◯ 答えたくない

「外国人」であるというだけで差別を受けたと感じましたか？
◯はい ◯いいえ ◯ 答えたくない

仕事を失い失業しましたか？
◯はい ◯いいえ ◯ 答えたくない

家庭内暴力（ＤＶ）を受けましたか?
◯はい ◯いいえ ◯ 答えたくない

セクハラを受けましたか？
◯はい ◯いいえ ◯ 答えたくない

スピリチュアル・宗教的な気持ちになりましたか?
◯はい ◯いいえ ◯ 答えたくない

SECTION 4: 生活の変化について

24. あなたの最近（または現在）の感情や気持ちについて教えてください。

気が落ち着いていますか？
◯全く違う ◯いくらかそうだ ◯まあそうだ ◯その通りだ

緊張していますか？
◯全く違う ◯いくらかそうだ ◯まあそうだ ◯その通りだ

気が転倒していますか？
◯全く違う ◯いくらかそうだ ◯まあそうだ ◯その通りだ

くつろいだ気持ちですか？
◯全く違う ◯いくらかそうだ ◯まあそうだ ◯その通りだ

満ち足りた気分ですか？
◯全く違う ◯いくらかそうだ ◯まあそうだ ◯その通りだ

心配がありますか？
◯全く違う ◯いくらかそうだ ◯まあそうだ ◯その通りだ

孤独を感じていますか？
◯全く違う ◯いくらかそうだ ◯まあそうだ ◯その通りだ

孤立していると感じますか？
◯全く違う ◯いくらかそうだ ◯まあそうだ ◯その通りだ

新型コロナウイルスへの感染の不安がありますか？
◯全く違う ◯いくらかそうだ ◯まあそうだ ◯その通りだ

25. あなたの日常生活の問題について

睡眠時間は？

○以前より短くなった ○ 変化なし ○ 以前より長くなった○ 該当しない ○ 答えたくない

体重は？

○以前より減った ○ 変化なし ○ 以前より増えた○ 該当しない ○ 答えたくない

お酒類を飲む量は？

○以前より減った ○ 変化なし ○ 以前より増えた○ 該当しない ○ 答えたくない

タバコを吸う量は？

○以前より減った ○ 変化なし ○ 以前より増えた○ 該当しない ○ 答えたくない

ゲームで遊ぶ・スマートフォンやテレビを見る時間は？

○以前より短くなった ○ 変化なし ○ 以前より長くなった○ 該当しない ○ 答えたくない

体を動かす・運動する時間は？

○以前より短くなった ○ 変化なし ○ 以前より長くなった○ 該当しない ○ 答えたくない

子育てや育児の時間は？

○以前より短くなった ○ 変化なし ○ 以前より長くなった○ 該当しない ○ 答えたくない

家庭内での争いやケンカは？

○以前より減った ○ 変化なし ○ 以前より増えた○ 該当しない ○ 答えたくない

友達や近所の人とのコミュニケーションは？

○以前より減った ○ 変化なし ○ 以前より増えた○ 該当しない ○ 答えたくない

全体的な心の健康状態は？

○以前より悪い ○ 変化なし ○ 以前より良い○ 該当しない ○ 答えたくない

全体的な身体の健康状態は？

○以前より悪い ○ 変化なし ○ 以前より良い○ 該当しない ○ 答えたくない

全体的な感情の健康状態は？ （楽しい・嬉しいなどの感情）

○以前より悪い ○ 変化なし ○ 以前より良い○ 該当しない ○ 答えたくない

全体的な性的満足度は？

◯以前より減った ◯ 変化なし ◯ 以前より増えた ◯ 該当しない ◯ 答えたくない

個人収入は？

◯以前より減った ◯ 変化なし ◯ 以前より増えた ◯ 該当しない ◯ 答えたくない

家庭での全体的な経済的安定は？

◯以前より不安定 ◯ 変化なし ◯ 以前より安定 ◯ 該当しない ◯ 答えたくない

26. 一緒に暮らしている 18 歳未満の子どもの人数を教えてください。

◯0 人
◯1 人
◯2 人
◯3 人
◯4 人
◯5 人またはそれ以上

27. あなたの子どもの問題について

子どもの睡眠時間は？
◯以前より短くなった ◯ 変化なし ◯ 以前より長くなった◯ 該当しない ◯ 答えたくない

子どもがゲームで遊ぶ・スマートフォンやテレビを見る時間は？
◯以前より短くなった ◯ 変化なし ◯ 以前より長くなった◯ 該当しない ◯ 答えたくない

子どもが体を動かす・運動する時間は？
◯以前より短くなった ◯ 変化なし ◯ 以前より長くなった◯ 該当しない ◯ 答えたくない

子どもの感情の起伏（暴言・怒り）は？
◯以前より激しくない ◯ 変化なし ◯ 以前より激しい ◯ 該当しない ◯ 答えたくない

子どもの友達とのコミュニケーションは？
◯以前より減った ◯ 変化なし ◯ 以前より増えた◯ 該当しない ◯ 答えたくない

子どもの家族とのコミュニケーションは？
◯以前より減った ◯ 変化なし ◯ 以前より増えた◯ 該当しない ◯ 答えたくない

子どもの学校生活は？
◯以前より短くなった ◯ 変化なし ◯ 以前より長くなった◯ 該当しない ◯ 答えたくない

子どもの全体的な心の健康状態は？
◯以前より悪い ◯ 変化なし ◯ 以前より良い◯ 該当しない ◯ 答えたくない

子どもの全体的な身体の健康状態は？
◯以前より悪い ◯ 変化なし ◯ 以前より良い◯ 該当しない ◯ 答えたくない

子どもの全体的な感情の健康状態は？ （楽しい・嬉しいなどの感情）
◯以前より悪い ◯ 変化なし ◯ 以前より良い◯ 該当しない ◯ 答えたくない

28. その他に困っていることや問題があれば、記入してください。

私たちＮＰＯ職員に直接相談したい場合は、下記にご連絡ください。
Email:　info@universalaid.jp; Tel: 090-2900-3934

私たちは、新型コロナウイルス感染拡大下という困難な状況でも、外国人の皆さまが安心して生活できる環境づくりのために必要なサポートを提供していきたいと願っています。

アンケートにご協力いただき、ありがとうございます。

COVID-19 감염 확산으로 인한 일본내 외국인 거주자의 문제와 불안에 관한 조사 : 횡단 연구

UNIVERSALAID.JP 와 나가사키 대학은 COVID-19 대유행으로 인해 일본에 거주하는(특히 나가사키시) 외국인의 문제와 불안을 확인하기 위해 설문 조사를 실시하고 문제와 불안을 해소를 위한 제안과 상담을 제공하기 위해 노력하고 있습니다.

설문 조사는 28 개의 질문으로 구성되었으며 약 7-10 분 정도 소요됩니다. 귀하는 언제든지 조사 참여를 거부하거나 철회 할 수 있습니다. 이 설문 조사에서 수집 된 응답은 기밀이며 어떠한 조건에서도 공개되지 않습니다. 또한 설문 조사는 완전히 익명으로 처리되며 관련 위험이 없습니다.

URL 또는 QR 코드를 통해 설문에 참여해 주십시오.
https://universalaid.jp/survey2020/
추가 문의 사항이 있으시면 Ms. Yoshimi Matsuo, UNIVERSALAID.JP 로 이메일을 보내 주십시오. (Email: info@universalaid.jp; Tel: 090-2900-3934) or Dr. Nguyen Tien Huy, Nagasaki University (Email: tienhuy@nagasaki-u.ac.jp).

이 설문 조사를 완료하고 제출함으로써 귀하는 설문 조사 참여에 동의하는 것입니다.

1. 귀하는 18 세 이상이며 이번 연구 참가에 동의하십니까?

○ 네
○ 아니오

섹션 1: 일반 정보

2. 귀하의 국적은 어디입니까?

▫ 대답하고 싶지 않음

3. 현재 어디에 살고 있습니까?

- ◯ 나가사키시
- ◯ 나가사키현내의 다른 도시
- ◯ 다른 도시
- ◯ 대답하고 싶지 않음

4. 연령

- ◯ 18-24 세
- ◯ 25-34 세
- ◯ 35-44 세
- ◯ 45-54 세
- ◯ 55-64 세
- ◯ 65 세이상
- ◯ 대답하고 싶지 않음

5. 성별

- ◯ 남성
- ◯ 여성
- ◯ 대답하고 싶지 않음
- ◯ 그 외 (명시해 주십시오)

6. 결혼 상태

○ 미혼, 결혼력이 없음
○ 기혼 또는 동거
○ 사별
○ 이혼
○ 별거
○ 대답하고 싶지 않음

7. 귀하의 배우자/파트너의 국적은 어디입니까?

○ 일본인
○ 귀하와 같은 국적
○ 현재 배우자/파트너가 없음
○ 대답하고 싶지 않음
○ 그 외 (명시해 주십시오)

8. 직업 (복수 선택 가능)

○ 정직원
○ 아르바이트/비정규직
○ 자영업
○ 전업주부
○ 무직
○ 학생
○ 은퇴
○ 대답하고 싶지 않음
○ 그 외 (명시해 주십시오)

9. 교육

- ◯ 대학원 혹은 이상
- ◯ 전문대학/대학 학위
- ◯ 고등학교
- ◯ 중학교
- ◯ 유치원/초등학교
- ◯ 교육력 없음
- ◯ 대답하고 싶지 않음

10. 이민 상황

- ◯ 귀화
- ◯ 영주권
- ◯ 취로 비자
- ◯ 유학 비자
- ◯ 가족 부양 비자
- ◯ 임시 비자 (관광, 비지니스)
- ◯ 대답하고 싶지 않음
- ◯ 그 외 (명시해 주십시오)

11. 어떤 종류의 건강 보험을 가지고 있습니까? (복수 선택 가능)

- ◯ 국민 건강 보험
- ◯ 직장 건강 보험
- ◯ 개인 건강 보험
- ◯ 여행자 건강 보험
- ◯ 없음
- ◯ 대답하고 싶지 않음

12. 얼마동안 일본에 거주하고 있습니까?

- ◯ 1 년 미만
- ◯ 1-2 년
- ◯ 3-5 년
- ◯ 6-10 년
- ◯ 10 년 이상
- ◯대답하고 싶지 않음

13. 당신의 일본어 능력은 어떻습니까?

- ◯ 일본인과 같은 수준으로 말할 수 있습니다.
- ◯ 일이나 학습면에 있어서 충분히 말할 수 있습니다.
- ◯ 일상 생활에 지장이 없을 만큼 충분히 말할 수 있습니다.
- ◯ 일본어를 잘 못합니다.
- ◯ 일본어를 전혀 못합니다.
- ◯대답하고 싶지 않음

14. 집/방에 거주하는 총 인원 (귀하 포함).

- ▫ 1
- ▫ 2
- ▫ 3
- ▫ 4
- ▫ 5 명 이상

15. 함께 거주하는 65 세 이상 고령자 인원

▫ 0
▫ 1
▫ 2
▫ 3
▫ 4
▫ 5 명 이상

16. 다른 가족(배우자, 자매, 형제)은 어디에 거주하고 있습니까? (복수 선택 가능)

-다른 가족은 없습니다
-일본에서 저와 함께 거주합니다
-일본 다른 곳에서 거주합니다
-본국
-다른 나라
-잘 모릅니다
-대답하고 싶지 않음

17. 언제 다른 가족들(배우자, 형제, 자매)과 마지막으로 만났습니까?

◯ 1 년 미만
◯ 1-2 년
◯ 3-5 년
◯ 6-10 년
◯ 10 년 이상
◯대답하고 싶지 않음

18. 얼마나 자주 가족들과 연락합니까(배우자, 형제, 자매)?

- ○ 매일
- ○ 매주
- ○ 매달
- ○ 일년에 몇 번
- ○ 기억 안남
- ○ 전혀
- ○ 대답하고 싶지 않음

섹션 2: 정보 네트워크

19. 귀하의 문제를 공유할 사람이 있습니까?

◯네

◯아니오

◯대답하고 싶지 않음

20. 귀하의 문제를 누구와 상의합니까? (복수 선택 가능)

-어머니

-아버지

-자매

-형제

-사촌

-다른 친척

-선생님

-동료

-일본인 친구

-본국의 친구

-종교 지도자

-정부 상담자

-대답하고 싶지 않음

-그 외 (명시해 주십시오)

21. COVID-19 관한 정보는 어디에서 얻습니까? (복수 선택 가능)

-본국의 가족

-일본내 가족

-일본인 친구

-일본내 같은 국적의 친구

-단체, 시설 혹은 같은 국적이 모이는 상점

-시청 및 현청

-시청 및 현청이 발행하는 간행물 혹은 웹사이트

-국제 교류 NPO

-텔레비젼, 신문, 잡지 및 인터넷

-SNS (Twitter, Facebook, Instagram, and 등)

-정보를 얻는 방법을 모릅니다.

-대답하고 싶지 않음

-그 외 (명시해 주십시오)

22. 모국어로 어떠한 정보를 원합니까? (복수 선택 가능)

- -COVID-19 증상이 의심되는 경우 대처 방법
- -이번 COVID-19 기간에 COVID-19 이외의 질병이나 부상이 발생한 경우 대처 방법
- -재해 발생 시 대처 방법
- -병원/클리닉에 가는 방법
- -이웃과의 어려움, 가정 폭력, 성희롱 등의 대처 방법
- -분만 및 출산
- -자녀에게 문제가 생겼을 때 대처 방법
- -자녀를 위한 교육 및 학교
- -COVID-19 의 감염 통제 상황
- -COVID-19 주발생 지역
- -COVID-19 예방 조치
- -COVID-19 관련 재정 지원
- -본국으로 귀국 방법
- -비자 연장 방법
- -일본에서 가족에게 연락하는 방법
- -대중 교통
- -운전 면허증 취득 방법
- -세금과 연금 제도
- -지역 이벤트
- -집 구하는 방법
- -직업 정보 및 구직 방법
- -국민 및 민간 건강 보험에 가입하는 방법
- -대답하고 싶지 않음
- -그 외 (명시해 주십시오)

섹션 3:간단 질문

23. 최근에?

-COVID-19 에 감염된 가족이 있습니까?

- 네
- 아니오
- 대답하고 싶지 않음

-COVID-19 감염이 의심되는 가족이 있었지만 검사를 받을 수 없었습니까?

- 네
- 아니오
- 대답하고 싶지 않음

-배우거나 일하는 데 문제 혹은 어려움이 있습니까?

- 네
- 아니오
- 대답하고 싶지 않음

-단순히 일본인이 아니라는 이유로 차별을 받았다고 느낀 적이 있습니까?

- 네
- 아니오
- 대답하고 싶지 않음

-직업을 잃었습니까?

- 네
- 아니오
- 대답하고 싶지 않음

-가정내 폭력이 있었습니까?

- 네
- 아니오
- 대답하고 싶지 않음

-성희롱 및 성폭력이 있었습니까?

- ○ 네
- ○ 아니오
- ○ 대답하고 싶지 않음

-이전 보다 영적/종교적 활동을 느꼈습니까?

- ○ 네
- ○ 아니오
- ○ 대답하고 싶지 않음

섹션 4:개인의 생활 변화

24. 최근 (또는 현재) 감정/기분을 설명해주십시오.

-평온하다

- ○ 전혀
- ○ 조금
- ○ 적당히
- ○ 매우

-긴장하다

- ○ 전혀
- ○ 조금
- ○ 적당히
- ○ 매우

-화가난다

- ○ 전혀
- ○ 조금
- ○ 적당히
- ○ 매우

-편안하다

- ○ 전혀
- ○ 조금

- 적당히
- 매우

-만족하다

- 전혀
- 조금
- 적당히
- 매우

-걱정하다

- 전혀
- 조금
- 적당히
- 매우

-외롭다

- 전혀
- 조금
- 적당히
- 매우

-소외되다

- 전혀
- 조금
- 적당히
- 매우

-COVID-19 감염이 두렵다

- 전혀
- 조금
- 적당히
- 매우

25. 귀하의 문제 수준

-수면 시간

- 이전보다 줄었다
- 이전과 같다
- 이전보다 늘었다
- 해당 없음
- 대답하고 싶지 않음

-체중

- 이전보다 줄었다
- 이전과 같다
- 이전보다 늘었다
- 해당 없음
- 대답하고 싶지 않음

-음주

- 이전보다 줄었다
- 이전과 같다
- 이전보다 늘었다
- 해당 없음
- 대답하고 싶지 않음

-흡연

- 이전보다 줄었다
- 이전과 같다
- 이전보다 늘었다
- 해당 없음
- 대답하고 싶지 않음

-게임 또는 스마트폰/티비 시청 시간

- ○ 이전보다 줄었다
- ○
- ○ 이전과 같다
- ○ 이전보다 늘었다
- ○ 해당 없음
- ○ 대답하고 싶지 않음

-신체 활동/운동

- ○ 이전보다 줄었다
- ○ 이전과 같다
- ○ 이전보다 늘었다
- ○ 해당 없음
- ○ 대답하고 싶지 않음

-가족 요양 혹은 육아 시간

- ○ 이전보다 줄었다
- ○ 이전과 같다
- ○ 이전보다 늘었다
- ○ 해당 없음
- ○ 대답하고 싶지 않음

-가족내 갈등/다툼

- ○ 이전보다 줄었다
- ○ 이전과 같다
- ○ 이전보다 늘었다
- ○ 해당 없음
- ○ 대답하고 싶지 않음

-이웃 및 친구들과의 대화

- ○ 이전보다 줄었다
- ○ 이전과 같다
- ○ 이전보다 늘었다
- ○ 해당 없음
- ○ 대답하고 싶지 않음

-전반적인 정신 건강

- ◯ 이전보다 줄었다
- ◯ 이전과 같다
- ◯ 이전보다 늘었다
- ◯ 해당 없음
- ◯ 대답하고 싶지 않음

-전반적인 신체 건강

- ◯ 이전보다 줄었다
- ◯ 이전과 같다
- ◯ 이전보다 늘었다
- ◯ 해당 없음
- ◯ 대답하고 싶지 않음

-전반적인 정서적 건강(행복)

- ◯ 이전보다 줄었다
- ◯ 이전과 같다
- ◯ 이전보다 늘었다
- ◯ 해당 없음
- ◯ 대답하고 싶지 않음

-전반적인 성적 만족

- ◯ 이전보다 줄었다
- ◯ 이전과 같다
- ◯ 이전보다 늘었다
- ◯ 해당 없음
- ◯ 대답하고 싶지 않음

-개인 소득

- ◯ 이전보다 줄었다
- ◯ 이전과 같다
- ◯ 이전보다 늘었다
- ◯ 해당 없음
- ◯ 대답하고 싶지 않음

-가구의 전반적인 재정적 안정성

- ○ 이전보다 줄었다
- ○ 이전과 같다
- ○ 이전보다 늘었다
- ○ 해당 없음
- ○ 대답하고 싶지 않음

26. 함께 살고 있는 18 세 미만의 자녀 수

- ▢ 1
- ▢ 2
- ▢ 3
- ▢ 4
- ▢ 5 명 이상

27. 자녀의 문제 수준

-자녀의 수면 시간

- ○ 이전보다 줄었다
- ○ 이전과 같다
- ○ 이전보다 늘었다
- ○ 해당 없음
- ○ 대답하고 싶지 않음

-자녀의 게임 또는 스마트폰/티비 시청 시간

- ○ 이전보다 줄었다
- ○ 이전과 같다
- ○ 이전보다 늘었다
- ○ 해당 없음
- ○ 대답하고 싶지 않음

-자녀의 신체 활동/운동

- 이전보다 줄었다
- 이전과 같다
- 이전보다 늘었다
- 해당 없음
- 대답하고 싶지 않음

-자녀의 폭발/분노

- 이전보다 줄었다
- 이전과 같다
- 이전보다 늘었다
- 해당 없음
- 대답하고 싶지 않음

-자녀의 친구들과의 대화

- 이전보다 줄었다
- 이전과 같다
- 이전보다 늘었다
- 해당 없음
- 대답하고 싶지 않음

-자녀의 가족들과의 대화

- 이전보다 줄었다
- 이전과 같다
- 이전보다 늘었다
- 해당 없음
- 대답하고 싶지 않음

-자녀의 학교 생활

- ○ 이전보다 줄었다
- ○ 이전과 같다
- ○ 이전보다 늘었다
- ○ 해당 없음
- ○ 대답하고 싶지 않음

-자녀의 전반적인 정신 건강

- ○ 이전보다 줄었다
- ○ 이전과 같다
- ○ 이전보다 늘었다
- ○ 해당 없음
- ○ 대답하고 싶지 않음

-자녀의 전반적인 신체 건강

- ○ 이전보다 줄었다
- ○ 이전과 같다
- ○ 이전보다 늘었다
- ○ 해당 없음
- ○ 대답하고 싶지 않음

-자녀의 전반적인 정서적 건강(행복)

- ○ 이전보다 줄었다
- ○ 이전과 같다
- ○ 이전보다 늘었다
- ○ 해당 없음
- ○ 대답하고 싶지 않음

28. 그 외 문제 (자유롭게 적어주세요)

저희 NPO 멤버와 직접 상담을 원하시면 연락
바랍니다 (Email:info@universalaid.jp; Tel: 090-2900-3934).

저희는 이번 COVID-19 기간에도 일본내 외국 국적 거주자 여러분들이 쾌적한 생활을 할 수 있도록 필요한 지원을 하고 싶습니다.

당신의 친절한 협력에 미리 감사드립니다.

ແບບຟອມການສຳຫຼວດເຖິງບັນຫາ ແລະ ຄວາມວິຕົກກັງວົນຂອງຄົນຕ່າງປະເທດທີ່ອາໃສຢູ່ໃນຍີ່ປຸ່ນ

ພາຍໃຕ້ສະຖານະການ ໂຄວິດ- 19 (COVID-19)

ພວກເຮົາອົງການຊ່ວຍເຫຼືອສາກົນ.ຍີ່ປຸ່ນ (UNIVERSALAID.JP) ແລະ ມະຫາວິທະຍາໄລ ນາງາຊາກິ (Nagasaki), ກຳລັງດຳເນີນການສຳຫຼວດນີ້ ເພື່ອກຳນົດບັນຫາ ແລະ ຄວາມກັງວົນໃຈຂອງຜູ້ທີ່ບໍ່ແມ່ນຄົນຍີ່ປຸ່ນ ພັກອາໃສຢູ່ໃນປະເທດຍີ່ປຸ່ນ (ໂດຍສະເພາະແມ່ນຢູ່ໃນເມືອງ ນາງາຊາກິ) ທີ່ໄດ້ຮັບຜົນກະທົບຈາກການລະບາດຂອງພະຍາດ ໂຄວິດ -19, ຈຸດປະສົງຂອງການສຳຫຼວດນີ້ແມ່ນເພື່ອຈະຈັດຫາຄຳແນະນຳ ແລະ ໃຫ້ຄຳປຶກສາໃນການແກ້ໄຂບັນຫາ ແລະ ຫຼຸດຜ່ອນຄວາມວິຕົກກັງວົນຕ່າງໆ.

ການສຳຫຼວດປະກອບມີ 28 ຄຳຖາມ ແລະ ມັນອາດຈະໃຊ້ເວລາ ປະມານ 7-10 ນາທີ ໃນການຕອບຄຳຖາມ. ທ່ານມີສິດທີ່ຈະປະຕິເສດ ຫຼື ຖອນຕົວອອກຈາກການມີສ່ວນຮ່ວມການສຳຫຼວດໃນຄັ້ງນີ້ໄດ້ທຸກເວລາ. ຄຳຕອບທັງໝົດທີ່ໄດ້ຮັບຈາກການສຳຫຼວດນີ້ຈະຖືກເກັບເປັນຄວາມລັບ ແລະ ຂໍ້ມູນນີ້ຈະບໍ່ຖືກເປີດເຜີຍບໍ່ວ່າຈະຢູ່ພາຍໃຕ້ສະພາບການໃດກໍ່ຕາມ. ນອກຈາກນັ້ນ, ການສຳຫຼວດຈະບໍ່ລະບຸຊື່ ແລະ ຈະບໍ່ມີຄວາມສ່ຽງໃດໆເກີດຂື້ນທັງໝົດ.

ກະລຸນາເຂົ້າຮ່ວມໃນການສຳຫຼວດໂດຍການຕິດຕາມ URL ຫຼື QR Code.

https://universalaid.jp/survey2020/

ສຳລັບການສອບຖາມເພີ່ມຕື່ມທ່ານສາມາດຕິດຕໍ່ທີມງານຂອງພວກເຮົາໂດຍການສົ່ງອີເມວຫາ

ທ່ານນາງ. Yoshimi Matsuo, UNIVERSALAID.JP (Email: info@universalaid.jp;

ໂທ: 090-2900-3934) ຫຼື ທ່ານ ດຣ Nguyen Tien Huy, ມະຫາວິທະຍາໄລ ນາງາຊາກິ (Nagasaki)

(Email: tienhuy@nagasaki-u.ac.jp).

ການຕອບ ແລະ ສົ່ງບົດສຳຫຼວດແມ່ນໝາຍຄວາມວ່າທ່ານຍິນຍອມທີ່ຈະເຂົ້າຮ່ວມໃນການສຳຫຼວດໃນຄັ້ງນີ້.

1. ທ່ານມີອາຍຸຫຼາຍກວ່າ 18 ປີ ແລະ ທ່ານຕົກລົງເຫັນດີທີ່ຈະເຂົ້າຮ່ວມໃນໂຄງການຄົ້ນຄ້ວານີ້ ຫຼື ບໍ່?

- ○ ແມ່ນແລ້ວ
- ○ ບໍ່ແມ່ນ

ພາກທີ 1: ຂໍ້ມູນທົ່ວໄປ

2. ປະເທດຕົ້ນກຳເນີດຂອງທ່ານແມ່ນປະເທດໃດ?

▢ ຂໍບໍ່ຕອບ

3. ທ່ານອາໃສຢູ່ໃສ?

- ○ ເມືອງ ນາງາຊາກິ (Nagasaki)
- ○ ເມືອງອື່ນໆຂອງແຂວງ ນາງາຊາກິ (Nagasaki)
- ○ ແຂວງອື່ນໆ
- ○ ຂໍບໍ່ຕອບ

4. ເກນອາຍຸຂອງທ່ານເທົ່າໃດ (ປີ)?

- ○ 18 - 24 ປີ
- ○ 25 - 34 ປີ
- ○ 35 - 44 ປີ
- ○ 45 - 54 ປີ
- ○ 55 - 64 ປີ
- ○ ອາຍຸ 65 ປີຂຶ້ນໄປ
- ○ ຂໍບໍ່ຕອບ

5. ເພດ

- ○ ຊາຍ
- ○ ຍິງ
- ○ ຂໍບໍ່ຕອບ
- ○ ອື່ນ ໆ (ກະລຸນາລະບຸ)

6. ສະຖານະພາບການແຕ່ງງານ

- ໂສດ, ບໍ່ເຄີຍແຕ່ງງານ
- ແຕ່ງງານແລ້ວ ຫຼື ມີຄູ່ຮັກແຕ່ຍັງບໍ່ໄດ້ແຕ່ງງານ
- ໝ້າຍ
- ຢ່າຮ້າງ
- ແຍກກັນຢູ່
- ຂໍບໍ່ຕອບ

7. ສັນຊາດຂອງຜົວ / ເມຍຂອງທ່ານແມ່ນສັນຊາດໃດ?

- ຍີ່ປຸ່ນ
- ສັນຊາດດຽວກັນກັບຕົນເອງ
- ຂ້ອຍບໍ່ມີຄູ່ສົມລົດ / ບໍ່ມີຄູ່ຄອງ
- ຂໍບໍ່ຕອບ
- ອື່ນ ໆ (ກະລຸນາ ລະບຸ)...

8. ວຽກ / ອາຊີບ (ສາມາດເລືອກໜຶ່ງ ຫຼື ຫຼາຍຂໍ້ໄດ້)

- ພະນັກງານເຮັດວຽກເຕັມເວລາ
- ພະນັກງານເຮັດວຽກນອກເວລາ / ຮັບຈ້າງທົ່ວໄປ
- ເຮັດວຽກອິດສະຫຼະ (ທຸລະກິດຕົນເອງ)
- ແມ່ເຮືອນ / ພໍ່ເຮືອນ
- ຫວ່າງງານ
- ນັກຮຽນ/ນັກສຶກສາ
- ພະນັກງານບໍານານ
- ຂໍບໍ່ຕອບ
- ອື່ນ ໆ (ກະລຸນາລະບຸ)

9. ການສຶກສາ

- ຈົບເໜືອປະລິນຍາຕີຂຶ້ນໄປ/ຈົບມະຫາວິທະຍາໄລ ຫຼື ສູງກວ່າ
- ລະດັບວິທະຍາໄລ / ມະຫາວິທະຍາໄລ

- ມັດທະຍົມຕອນປາຍ
- ມັດທະຍົມຕອນຕົ້ນ
- ໂຮງຮຽນປະຖົມ / ລະດັບປະຖົມ
- ບໍ່ໄດ້ຮຽນ
- ຂໍບໍ່ຕອບ

10. ສະຖານະພາບການເຂົ້າເມືອງ

- ໂອນສັນຊາດແລ້ວ
- ຢູ່ແບບຖາວອນ
- ວີຊ່າ ເຮັດວຽກ
- ວີຊ່ານັກຮຽນ/ ນັກສຶກສາ
- ວີຊ່າຢູ່ນຳຄອບຄົວ
- ວີຊ່າຊົ່ວຄາວ (ນັກທ່ອງທ່ຽວ, ທຸລະກິດ)
- ຂໍບໍ່ຕອບ
- ອື່ນ ໆ (ກະລຸນາລະບຸ)

11. ທ່ານມີປະກັນສຸຂະພາບປະເພດໃດ? (ສາມາດເລືອກໜຶ່ງ ຫຼື ຫຼາຍຂໍ້)

- ປະກັນສຸຂະພາບແຫ່ງຊາດ
- ປະກັນສຸຂະພາບຂອງພະນັກງານ
- ປະກັນສຸຂະພາບເອກະຊົນ
- ປະກັນສຸຂະພາບການເດີນທາງ
- ບໍ່ມີ
- ຂໍບໍ່ຕອບ

12. ທ່ານອາໄສຢູ່ຍີ່ປຸ່ນດົນປານໃດ?

- ໜ້ອຍກວ່າ 1 ປີ
- 1 - 2 ປີ
- 3 - 5 ປີ
- 6 - 10 ປີ
- ຫຼາຍກ່ວາ 10 ປີ
- ຂໍບໍ່ຕອບ

13. ລະດັບພາສາຍີ່ປຸ່ນຂອງທ່ານແມ່ນລະດັບໃດ?

- ຂ້ອຍສາມາດເວົ້າໄດ້ໃນລະດັບດຽວກັນກັບຄົນຍີ່ປຸ່ນ.
- ຂ້ອຍສາມາດເວົ້າໄດ້ດີພໍສໍາລັບການເຮັດວຽກ ຫຼື ການຮຽນ.
- ຂ້ອຍສາມາດເວົ້າໄດ້ດີພໍທີ່ຈະບໍ່ມີບັນຫາໃນຊີວິດປະຈໍາວັນ.
- ຂ້ອຍບໍ່ສາມາດເວົ້າພາສາຍີ່ປຸ່ນໄດ້ດີປານໃດ.
- ຂ້ອຍບໍ່ສາມາດເວົ້າພາສາຍີ່ປຸ່ນໄດ້ເລີຍ.
- ຂໍບໍ່ຕອບ

14. ຈໍານວນຄົນທັງໝົດທີ່ອາໄສຢູ່ໃນເຮືອນ / ຫ້ອງຂອງທ່ານ (ລວມທັງຕົວທ່ານເອງ) ມີທັງໝົດຈັກຄົນ?

▢ 1

▢ 2

▢ 3

▢ 4

▢ 5 ຄົນຂຶ້ນໄປ

15. ຈໍານວນຄົນທີ່ມີອາຍຸ 65 ປີຂຶ້ນໄປ ທີ່ອາໄສຢູ່ນໍາທ່ານ.

▢ 0

▢ 1

▢ 2

▢ 3

▢ 4

▢ 5 ຄົນຂຶ້ນໄປ

16. ປະຈຸບັນນີ້ສະມາຊິກຄອບຄົວຄົນອື່ນໆ (ພໍ່ແມ່, ເອື້ອຍ, ອ້າຍ, ນ້ອງ) ອາໄສຢູ່ໃສ? (ສາມາດເລືອກໜຶ່ງ ຫຼື ຫຼາຍຂໍ້ໄດ້)

- ຂ້ອຍບໍ່ມີສະມາຊິກຄອບຄົວຄົນອື່ນ.
- ອາໄສຢູ່ກັບຂ້ອຍຢູ່ຍີ່ປຸ່ນ.
- ອາໄສຢູ່ເຮືອນອື່ນໃນປະເທດຍີ່ປຸ່ນ
- ຢູ່ປະເທດຂອງຂ້ອຍ
- ຢູ່ປະເທດອື່ນໆ
- ຂ້ອຍບໍ່ຮູ້.

- ຂໍບໍ່ຕອບ

17. ຄັ້ງສຸດທ້າຍທີ່ທ່ານໄດ້ພົບກັບສະມາຊິກຄອບຄົວຄົນອື່ນໆຂອງທ່ານ (ພໍ່ແມ່, ເອື້ອຍ, ອ້າຍ, ນ້ອງ) ແມ່ນຕອນໃດ?

- ໜ້ອຍກວ່າ 1 ປີ
- 1 - 2 ປີ
- 3 - 5 ປີ
- 6 - 10 ປີ
- ຫຼາຍກ່ວາ 10 ປີ
- ຂໍບໍ່ຕອບ

18. ທ່ານໂທຫາຄົນໃນຄອບຄົວຂອງທ່ານເລື້ອຍປານໃດ (ພໍ່ແມ່, ເອື້ອຍ, ອ້າຍ, ນ້ອງ)?

- ທຸກໆມື້
- ທຸກໆອາທິດ
- ທຸກໆເດືອນ
- 2 - 3 ເທື່ອ ຕໍ່ ປີ
- ບໍ່ສາມາດຈື່ໄດ້
- ບໍ່ເຄີຍ
- ຂໍບໍ່ຕອບ

ພາກທີ 2: ເຄືອຂ່າຍຂໍ້ມູນຂ່າວສານ

19. ເຈົ້າມີໃຜທີ່ຈະແບ່ງປັນບັນຫາຂອງເຈົ້າບໍ່?

- ມີ
- ບໍ່ມີ
- ຂໍບໍ່ຕອບ

20. ທ່ານສາມາດແບ່ງປັນບັນຫາຂອງທ່ານກັບໃຜ? (ສາມາດເລືອກໜຶ່ງ ຫຼື ຫຼາຍຂໍ້ໄດ້)

- ແມ່ຂອງຂ້ອຍ
- ພໍ່ຂອງຂ້ອຍ
- ເອື້ອຍຂອງຂ້ອຍ
- ອ້າຍຂອງຂ້ອຍ
- ນ້ອງຂອງຂ້ອຍ
- ພີ່ນ້ອງຂອງຂ້ອຍ
- ຍາດຕິພີ່ນ້ອງຄົນອື່ນໆ
- ຄູ, ອາຈານ
- ເພື່ອນຮ່ວມງານ
- ໝູ່ຄົນຍີ່ປຸ່ນ

- ໝູ່ຢູ່ບ້ານເກີດຂອງຂ້ອຍ
- ຜູ້ນຳທາງສາສະໜາ
- ທີ່ປຶກສາທາງພາກລັດ
- ຂໍບໍ່ຕອບ
- ອື່ນ ໆ (ກະລຸນາ ລະບຸ)..

21. ທ່ານໄດ້ຮັບຂໍ້ມູນກ່ຽວກັບ ໂຄວິດ- 19 (COVID-19) ຢູ່ໃສ? (ສາມາດເລືອກໜຶ່ງ ຫຼື ຫຼາຍຂໍ້ໄດ້)

- ຄອບຄົວຢູ່ປະເທດຂອງທ່ານ
- ຄອບຄົວຢູ່ຍີ່ປຸ່ນ
- ໝູ່ຄົນຍີ່ປຸ່ນ
- ໝູ່ເພື່ອນຈາກປະເທດດຽວກັນ
- ການຈັດຕັ້ງ, ຮ້ານຄ້າສະດວກຊື້ ຫຼື ຮ້ານຕ່າງໆ ບ່ອນທີ່ຜູ້ຄົນມາຈາກປະເທດດຽວກັນມາເຕົ້າໂຮມກັນ.
- ຫ້ອງການປົກຄອງເມືອງ ແລະ ເທດສະບານ
- ຂໍ້ມູນຂ່າວສານ, ວາລະສານ ຂອງສາທາລະນະ ແລະ ເວບໄຊທ໌ຂອງເທດສະບານ ແລະ ແຂວງ
- ອົງການສາກົນລະຫວ່າງປະເທດ NPO
- ໂທລະພາບ, ໜັງສືພິມ, ວາລະສານ, ຫຼື ອິນເຕີເນັດ
- ເຄືອຄ່າຍສັງຄົມອອນລາຍ SNS (Twitter, Facebook, Instagram ແລະ ອື່ນໆ)
- ຂ້ອຍບໍ່ຮູ້ວິທີທີ່ຈະໄດ້ຮັບຂໍ້ມູນ
- ຂໍບໍ່ຕອບ
- ອື່ນ ໆ (ກະລຸນາລະບຸ)

22. ທ່ານຕ້ອງການຂໍ້ມູນປະເພດໃດແດ່ໃນພາສາຂອງທ່ານ? (ສາມາດເລືອກໜຶ່ງ ຫຼື ຫຼາຍຂໍ້ໄດ້)

- ສິ່ງທີ່ຄວນເຮັດໃນກໍລະນີທີ່ທີ່ສົງໃສວ່າຂ້ອຍມີອາການຂອງ ໂຄວິດ - 19 (COVID-19)
- ສິ່ງທີ່ຄວນເຮັດໃນກໍລະນີທີ່ມີການເຈັບປ່ວຍ ຫຼື ການບາດເຈັບ ນອກເໜືອຈາກ ໂຄວິດ - 19 (COVID-19) ແລະ ໃນໄລຍະເວລາການລະບາດຂອງ COVID-19 ນີ້
- ສິ່ງທີ່ຄວນເຮັດໃນກໍລະນີເກີດໄພພິບັດ

- ສິ່ງທີ່ຄວນເຮັດເມື່ອໄປໂຮງໝໍ / ຄລີນິກ
- ສິ່ງທີ່ຄວນເຮັດໃນກໍລະນີທີ່ຂ້ອຍມີບັນຫາກັບເພື່ອນບ້ານ, ເກີດຄວາມຮຸນແຮງໃນຄອບຄົວ, ຫຼື ຖືກລ່ວງລະເມີດທາງເພດ
- ການເກີດລູກ
- ສິ່ງທີ່ຄວນເຮັດໃນກໍລະນີທີ່ລູກຂ້ອຍມີບັນຫາ
- ການສຶກສາ, ໂຮງຮຽນສໍາລັບເດັກນ້ອຍ
- ສະພາບການຄວບຄຸມການຕິດເຊື້ອຂອງ COVID-19
- ບ່ອນທີ່ມີຈຸດສຸມ COVID-19
- ມາດຕະການປ້ອງກັນຕໍ່ກັບ COVID-19
- ການຊ່ວຍເຫຼືອດ້ານການເງິນທີ່ກ່ຽວຂ້ອງກັບ COVID-19
- ເຮັດແນວໃດເພື່ອກັບຄືນປະເທດ
- ວິທີການຕໍ່ອາຍຸວີຊ່າ
- ການໂທຫາຄອບຄົວຈາກປະເທດຍີ່ປຸ່ນ
- ການຂົນສົ່ງສາທາລະນະ
- ເຮັດແນວໃດເພື່ອໃຫ້ໄດ້ຮັບໃບຂັບຂີ່
- ລະບົບອາກອນ ແລະ ເງິນບໍານານ
- ກິດຈະກໍາໃນຊຸມຊົນ
- ປະສົບການໃນການຊອກຫາເຮືອນ, ທີ່ຢູ່ອາໄສ
- ຂໍ້ມູນຂ່າວສານກ່ຽວກັບວຽກງານ ແລະ ປະສົບການໃນການຊອກຫາວຽກເຮັດງານທໍາ
- ວິທີການຊື້ປະກັນສຸຂະພາບແຫ່ງຊາດ ແລະ / ຫຼື ຂອງເອກະຊົນ
- ຂໍບໍ່ຕອບ
- ອື່ນ ໆ (ກະລຸນາລະບຸ)

ພາກສ່ວນທີ 3: ຄໍາຖາມສັ້ນໆ

23. ເມື່ອບໍ່ດົນມານີ້ທ່ານມີສິ່ງຕໍ່ໄປນີ້ບໍ່?

- ມີສະມາຊິກຄອບຄົວຄົນໜຶ່ງທີ່ໄດ້ຮັບການຢັ້ງຢືນວ່າຕິດ COVID-19 ຫຼື ບໍ່?
  - ມີ
  - ບໍ່ມີ
  - ຂໍບໍ່ຕອບ

- ມີສະມາຊິກໃນຄອບຄົວສົງໃສວ່າຕິດເຊື້ອ COVID-19 ແຕ່ບໍ່ສາມາດກວດໄດ້ບໍ?
  - ມີ
  - ບໍ່ມີ
  - ຂໍບໍ່ຕອບ

- ມີບັນຫາ / ຄວາມຫຍຸ້ງຍາກກ່ຽວກັບການຮຽນ ຫຼື ການເຮັດວຽກບໍ່?
  - ມີ
  - ບໍ່ມີ
  - ຂໍບໍ່ຕອບ

- ຮູ້ສຶກຖືກຈຳແນກຍ້ອນບໍ່ແມ່ນຄົນຍີ່ປຸ່ນບໍ?
  - ແມ່ນແລ້ວ
  - ບໍ່
  - ຂໍບໍ່ຕອບ

- ທ່ານຕົກວຽກບໍ?
  - ແມ່ນແລ້ວ
  - ບໍ່
  - ຂໍບໍ່ຕອບ

- ເກີດຄວາມຮຸນແຮງໃນຄອບຄົວຂອງທ່ານບໍ?
  - ແມ່ນແລ້ວ
  - ບໍ່
  - ຂໍບໍ່ຕອບ

- ທ່ານຖືກຂົ່ມເຫັງທາງເພດບໍ?
  - ແມ່ນແລ້ວ
  - ບໍ່
  - ຂໍບໍ່ຕອບ

- ທ່ານເຮັດກິດຈະກຳດ້ານຈິດວິນຍານ / ສາສະໜາຫຼາຍຂຶ້ນບໍ່?
  - ແມ່ນແລ້ວ
  - ບໍ່
  - ຂໍບໍ່ຕອບ

ພາກທີ 4: ການປ່ຽນແປງໃນຊີວິດສ່ວນຕົວ

24. ອະທິບາຍ ຄວາມຮູ້ສຶກ / ອາລົມ ໃນປະຈຸບັນຂອງທ່ານ

- ຂ້ອຍຮູ້ສຶກສະຫງົບ
  - ບໍ່ແມ່ນ
  - ແມ່ນເລັກໜ້ອຍ

- ປານກາງ
- ຫຼາຍ

- ຂ້ອຍຮູ້ສຶກເຄັ່ງຕຶງ
    - ບໍ່ແມ່ນ
    - ແມ່ນເລັກໜ້ອຍ
    - ປານກາງ
    - ຫຼາຍ

- ຂ້ອຍຮູ້ສຶກອຸກໃຈ
    - ບໍ່ແມ່ນ
    - ແມ່ນເລັກໜ້ອຍ
    - ປານກາງ
    - ຫຼາຍ

- ຂ້ອຍສະບາຍໃຈ
    - ບໍ່ແມ່ນ
    - ແມ່ນເລັກໜ້ອຍ
    - ປານກາງ
    - ຫຼາຍ

- ຂ້ອຍຮູ້ສຶກພໍໃຈ
    - ບໍ່ແມ່ນ
    - ແມ່ນເລັກໜ້ອຍ
    - ປານກາງ
    - ຫຼາຍ

- ຂ້ອຍຮູ້ສຶກກັງວົນ
    - ບໍ່ແມ່ນ
    - ແມ່ນເລັກໜ້ອຍ
    - ປານກາງ
    - ຫຼາຍ

- ຂ້ອຍຮູ້ສຶກເຫີງາ
    - ບໍ່ແມ່ນ
    - ແມ່ນເລັກໜ້ອຍ
    - ປານກາງ

- ຫຼາຍ

- ຂ້ອຍຮູ້ສຶກໂດດດ່ຽວ
  - ບໍ່ແມ່ນ
  - ແມ່ນເລັກໜ້ອຍ
  - ປານກາງ
  - ຫຼາຍ

- ຂ້ອຍຢ້ານທີ່ຈະຕິດເຊື້ອ COVID-19
  - ບໍ່ແມ່ນ
  - ແມ່ນເລັກໜ້ອຍ
  - ປານກາງ
  - ຫຼາຍ

25. ລະດັບບັນຫາຂອງທ່ານ

- ໄລຍະເວລານອນຫຼັບ
  - ໜ້ອຍກວ່າແຕ່ກ່ອນ
  - ຄືກັນກັບແຕ່ກ່ອນ
  - ຫຼາຍກວ່າແຕ່ກ່ອນ
  - ຕອບບໍ່ໄດ້
  - ຂໍບໍ່ຕອບ
- ນໍ້າໜັກຮ່າງກາຍ
  - ໜ້ອຍກວ່າແຕ່ກ່ອນ
  - ຄືກັນກັບແຕ່ກ່ອນ
  - ຫຼາຍກວ່າແຕ່ກ່ອນ
  - ຕອບບໍ່ໄດ້
  - ຂໍບໍ່ຕອບ
- ການບໍລິໂພກເຄື່ອງດື່ມມຶນເມົາ (ເຫຼົ້າ)
  - ໜ້ອຍກວ່າແຕ່ກ່ອນ
  - ຄືກັນກັບແຕ່ກ່ອນ
  - ຫຼາຍກວ່າແຕ່ກ່ອນ
  - ຕອບບໍ່ໄດ້
  - ຂໍບໍ່ຕອບ
- ການສູບຢາ
  - ໜ້ອຍກວ່າແຕ່ກ່ອນ
  - ຄືກັນກັບແຕ່ກ່ອນ
  - ຫຼາຍກວ່າແຕ່ກ່ອນ

- ຕອບບໍ່ໄດ້
- ຂໍບໍ່ຕອບ

- ຫຼິ້ນເກມ / ໃຊ້ເວລາໃນສະມາດໂຟນ (ໂທລະສັບ) / ໂທລະພາບ
  - ໜ້ອຍກວ່າແຕ່ກ່ອນ
  - ຄືກັນກັບແຕ່ກ່ອນ
  - ຫຼາຍກວ່າແຕ່ກ່ອນ
  - ຕອບບໍ່ໄດ້
  - ຂໍບໍ່ຕອບ
- ກິດຈະກຳທາງກາຍະພາບ / ອອກກຳລັງກາຍ
  - ໜ້ອຍກວ່າແຕ່ກ່ອນ
  - ຄືກັນກັບແຕ່ກ່ອນ
  - ຫຼາຍກວ່າແຕ່ກ່ອນ
  - ຕອບບໍ່ໄດ້
  - ຂໍບໍ່ຕອບ
- ໄລຍະເວລາການດູແລເບິ່ງແຍງລູກ/ເດັກ
  - ໜ້ອຍກວ່າແຕ່ກ່ອນ
  - ຄືກັນກັບແຕ່ກ່ອນ
  - ຫຼາຍກວ່າແຕ່ກ່ອນ
  - ຕອບບໍ່ໄດ້
  - ຂໍບໍ່ຕອບ
- ຄວາມຂັດແຍ່ງ / ຜິດຖຽງກັນໃນເຮືອນຂອງທ່ານ
  - ໜ້ອຍກວ່າແຕ່ກ່ອນ
  - ຄືກັນກັບແຕ່ກ່ອນ
  - ຫຼາຍກວ່າແຕ່ກ່ອນ
  - ຕອບບໍ່ໄດ້
  - ຂໍບໍ່ຕອບ
- ການຕິດຕໍ່ພົວພັນກັບເພື່ອນບ້ານ ຫຼື ໝູ່ເພື່ອນ
  - ໜ້ອຍກວ່າແຕ່ກ່ອນ
  - ຄືກັນກັບແຕ່ກ່ອນ
  - ຫຼາຍກວ່າແຕ່ກ່ອນ
  - ຕອບບໍ່ໄດ້
  - ຂໍບໍ່ຕອບ
- ສຸຂະພາບຈິດຂອງທ່ານໂດຍລວມ
  - ໜ້ອຍກວ່າແຕ່ກ່ອນ
  - ຄືກັນກັບແຕ່ກ່ອນ
  - ຫຼາຍກວ່າແຕ່ກ່ອນ
  - ຕອບບໍ່ໄດ້

- ຂໍບໍ່ຕອບ

- ສຸຂະພາບຮ່າງກາຍຂອງທ່ານໂດຍລວມ
  - ໜ້ອຍກວ່າແຕ່ກ່ອນ
  - ຄືກັນກັບແຕ່ກ່ອນ
  - ຫຼາຍກວ່າແຕ່ກ່ອນ
  - ຕອບບໍ່ໄດ້
  - ຂໍບໍ່ຕອບ
- ສຸຂະພາບດ້ານອາລົມຈິດຂອງທ່ານໂດຍລວມ (ຄວາມສຸກ)
  - ໜ້ອຍກວ່າແຕ່ກ່ອນ
  - ຄືກັນກັບແຕ່ກ່ອນ
  - ຫຼາຍກວ່າແຕ່ກ່ອນ
  - ຕອບບໍ່ໄດ້
  - ຂໍບໍ່ຕອບ

- ຄວາມເພິ່ງພໍໃຈທາງເພດຂອງທ່ານໂດຍລວມ
  - ໜ້ອຍກວ່າແຕ່ກ່ອນ
  - ຄືກັນກັບແຕ່ກ່ອນ
  - ຫຼາຍກວ່າແຕ່ກ່ອນ
  - ຕອບບໍ່ໄດ້
  - ຂໍບໍ່ຕອບ
- ລາຍໄດ້ສ່ວນຕົວ
  - ໜ້ອຍກວ່າແຕ່ກ່ອນ
  - ຄືກັນກັບແຕ່ກ່ອນ
  - ຫຼາຍກວ່າແຕ່ກ່ອນ
  - ຕອບບໍ່ໄດ້
  - ຂໍບໍ່ຕອບ
- ຄວາມໝັ້ນຄົງດ້ານການເງິນຂອງຄອບຄົວທ່ານໂດຍລວມ
  - ໜ້ອຍກວ່າແຕ່ກ່ອນ
  - ຄືກັນກັບແຕ່ກ່ອນ
  - ຫຼາຍກວ່າແຕ່ກ່ອນ
  - ຕອບບໍ່ໄດ້
  - ຂໍບໍ່ຕອບ

26. ຈຳນວນເດັກນ້ອຍອາຍຸຕ່ຳກວ່າ 18 ປີ ທີ່ອາໄສຢູ່ກັບທ່ານ.

▢ 0

▢ 1

▢ 2

▢ 3

▢ 4

▢ 5 ຄົນຂຶ້ນໄປ

27. ບັນຫາຂອງລູກ/ເດັກນ້ອຍຂອງທ່ານ

- ໄລຍະເວລານອນຂອງເດັກ
  - ຕ່ຳ / ໜ້ອຍກ່ວາແຕ່ກ່ອນ
  - ຄືກັນກັບແຕ່ກ່ອນ
  - ສູງກວ່າ / ຫຼາຍກວ່າແຕ່ກ່ອນ
  - ຕອບບໍ່ໄດ້
  - ຂໍບໍ່ຕອບ

- ເດັກນ້ອຍຫຼິ້ນເກມ / ໃຊ້ເວລາໃນສະມາດໂຟນ (ໂທລະສັບ) / ໂທລະພາບ
  - ຕ່ຳ / ໜ້ອຍກ່ວາແຕ່ກ່ອນ
  - ຄືກັນກັບແຕ່ກ່ອນ
  - ສູງກວ່າ / ຫຼາຍກວ່າແຕ່ກ່ອນ
  - ຕອບບໍ່ໄດ້
  - ຂໍບໍ່ຕອບ

- ກິດຈະກຳທາງດ້ານຮ່າງກາຍ / ການອອກກຳລັງກາຍຂອງເດັກ
  - ຕ່ຳ / ໜ້ອຍ ກ່ວາແຕ່ກ່ອນ
  - ຄືກັນກັບແຕ່ກ່ອນ
  - ສູງກວ່າ / ຫຼາຍກວ່າແຕ່ກ່ອນ
  - ຕອບບໍ່ໄດ້
  - ຂໍບໍ່ຕອບ

- ຄວາມຮຸນແຮງ / ຄວາມໃຈຮ້າຍຂອງເດັກ
  - ຕ່ຳ / ໜ້ອຍກ່ວາແຕ່ກ່ອນ
  - ຄືກັນກັບແຕ່ກ່ອນ
  - ສູງກວ່າ / ຫຼາຍກວ່າແຕ່ກ່ອນ
  - ຕອບບໍ່ໄດ້
  - ຂໍບໍ່ຕອບ

- ການສື່ສານກັບໝູ່ເພື່ອນຂອງເດັກ
  - ຕ່ຳ / ໜ້ອຍກວ່າແຕ່ກ່ອນ
  - ຄືກັນກັບແຕ່ກ່ອນ
  - ສູງກວ່າ / ຫຼາຍກວ່າແຕ່ກ່ອນ
  - ຕອບບໍ່ໄດ້
  - ຂໍບໍ່ຕອບ

- ການສື່ສານກັບບຸກຄົນທີ່ເປັນສະມາຊິກໃນຄອບຄົວຂອງເດັກ
  - ຕ່ຳ / ໜ້ອຍກວ່າແຕ່ກ່ອນ
  - ຄືກັນກັບແຕ່ກ່ອນ
  - ສູງກວ່າ / ຫຼາຍກວ່າແຕ່ກ່ອນ
  - ຕອບບໍ່ໄດ້
  - ຂໍບໍ່ຕອບ

- ຊີວິດໃນໂຮງຮຽນຂອງເດັກ
  - ຕ່ຳ / ໜ້ອຍກວ່າແຕ່ກ່ອນ
  - ຄືກັນກັບແຕ່ກ່ອນ
  - ສູງກວ່າ / ຫຼາຍກວ່າແຕ່ກ່ອນ
  - ຕອບບໍ່ໄດ້
  - ຂໍບໍ່ຕອບ

- ສຸຂະພາບຈິດຂອງເດັກໂດຍລວມ
  - ຕ່ຳ / ໜ້ອຍກວ່າແຕ່ກ່ອນ
  - ຄືກັນກັບແຕ່ກ່ອນ
  - ສູງກວ່າ / ຫຼາຍກວ່າແຕ່ກ່ອນ
  - ຕອບບໍ່ໄດ້
  - ຂໍບໍ່ຕອບ

- ສຸຂະພາບທາງຮ່າງກາຍຂອງເດັກໂດຍລວມ
  - ຕ່ຳ/ ໜ້ອຍກວ່າແຕ່ກ່ອນ
  - ຄືກັນກັບແຕ່ກ່ອນ
  - ສູງກວ່າ / ຫຼາຍກວ່າແຕ່ກ່ອນ
  - ຕອບບໍ່ໄດ້
  - ຂໍບໍ່ຕອບ

- ຄວາມສຸກທາງດ້ານຄວາມຮູ້ສຶກຂອງເດັກໂດຍລວມ
  - ຕ່ຳ / ໜ້ອຍ ກ່ວາແຕ່ກ່ອນ
  - ຄືກັນກັບແຕ່ກ່ອນ
  - ສູງກວ່າ / ຫຼາຍກວ່າແຕ່ກ່ອນ
  - ຕອບບໍ່ໄດ້
  - ຂໍບໍ່ຕອບ

28. ບັນຫາອື່ນໆ (ກະລຸນາຂຽນໃສ່ບ່ອນນີ້)

(Email: info@universalaid.jp; Tel: 090-2900-3934)

ພວກເຮົາມີຄວາມປາຖະໜາຢາກຈະໃຫ້ການສະໜັບສະໜູນທີ່ຈຳເປັນແກ່ຜູ້ທີ່ບໍ່ແມ່ນຄົນຍີ່ປຸ່ນ ແຕ່ອາໄສຢູ່ໃນປະເທດຍີ່ປຸ່ນ ເພື່ອໃຫ້ມີການດຳລົງຊີວິດທີ່ສະດວກສະບາຍ, ເຖີງແມ່ນວ່າຈະຢູ່ໃນຊ່ວງໄລຍະການແຜ່ລະບາດຂອງພະຍາດ ໂຄວິດ - 19 (COVID-19)

ຂອບໃຈລ່ວງໜ້າສຳລັບການຮ່ວມມືຂອງທ່ານ.

Masalah dan Kebimbangan pemastautin bukan Jepun yang Mengalami Situasi COVID-19: Tinjauan "Cross-Sectional"

Kami, UNIVERSALAID.JP dan Universiti Nagasaki sedang menjalankan tinjauan untuk mengenalpasti masalah dan kebimbangan pemastautin bukan Jepun yang menetap di Jepun (terutamanya di Bandar Nagasaki) disebabkan oleh pandemik COVID-19, dengan usaha untuk memberikan cadangan dan konsultasi bagi mengatasi masalah dan kebimbangan tersebut.

Tinjauan ini terdiri daripada 28 soalan dan akan mengambil masa lebih kurang 7-10 minit untuk menjawab kesemuanya. Anda berhak untuk menolak atau menarik diri daripada tinjauan ini pada bila-bila masa. Semua maklumat adalah sulit dan dirahsiakan. Tambahan pula, tinjauan ini dijalankan secara tanpa nama dan tiada risiko yang terlibat.

Sila sertai tinjauan ini melalui URL atau Kod QR berikut. https://universalaid.jp/survey2020/

Untuk sebarang pertanyaan , anda boleh menghubungi pasukan kami dengan menghantar Emel kepada Cik Yoshimi Matuso, UNIVERSALAID.JP (Email: info@universalaid.jp; Tel: 090-2900-3934) atau Dr.Nguyen Tien Huy, Universiti Nagasaki (Email: tienhuy@nagasaki-u.ac.jp).

Dengan melengkapkan dan menghantar tinjauan ini, anda bersetuju untuk mengambil bahagian dalam tinjauan ini.

1. Adakah anda berumur 18 tahun ke atas dan adakah anda bersetuju untuk mengambil bahagian dalam projek kajian ini?

○ YA
○ TIDAK

BAHAGIAN 1: MAKLUMAT UMUM

2. Apakah negara asal anda?

▢ Memilih untuk tidak menjawab

3. Dimanakah anda menetap sekarang?

- ◯ Bandar Nagasaki
- ◯ Bandar lain di wilayah Nagasaki
- ◯ Wilayah lain
- ◯ Memilih untuk tidak menjawab

4. Umur anda (tahun)

- 18-24 tahun
- 25-34 tahun
- 35-44 tahun
- 45-54 tahun
- 55-64 tahun
- 65 tahun atau lebih
- Memilih untuk tidak menjawab

5. Jantina

- Lelaki
- Perempuan
- Memilih untuk tidak menjawab
- Lain-lain (Sila nyatakan)

6. Status Perkahwinan

- ◯ Bujang, tidak pernah berkahwin
- ◯ Sudah berkahwin atau hubungan domestik
- ◯ Janda/Duda
- ◯ Bercerai
- ◯ Berpisah
- ◯ Memilih untuk tidak menjawab

7. Apakah kewarganegaraan asal pasangan anda?

- ◯ Jepun
- ◯ Kewarganegaraan sama seperti saya
- ◯ Saya tidak mempunyai pasangan
- ◯ Memilih untuk tidak menjawab
- ◯ Lain-lain

8. Status pekerjaan (Pilih satu atau lebih)

- ◯ Pekerja sepenuh masa
- ◯ Pekerja separuh masa/ sambilan
- ◯ Berkerja sendiri
- ◯ Suri rumah
- ◯ Tidak bekerja
- ◯ Pelajar
- ◯ Pesara
- ◯ Memilh untuk tidak menjawab
- ◯ Lain-lain (Sila nyatakan)

9. Tahap pendidikan

- ◯ Pascasiswazah atau lebih tinggi
- ◯ Ijazah universiti/ kolej
- ◯ Diploma atau setaraf
- ◯ Sekolah menengah atau setaraf
- ◯ Sekolah rendah atau setaraf
- ◯ Tidak bersekolah
- ◯ Memilih untuk tidak menjawab

10. Status Imigrasi

- ◯ Warganegara yang dinaturalisasikan
- ◯ Penduduk tetap
- ◯ Permit kerja
- ◯ Visa pelajar
- ◯ Visa tanggungan keluarga
- ◯ Visa sementara (pelancong, peniaga)
- ◯ Memilih untuk tidak menjawab
- ◯ Lain-lain (Sila nyatakan)

11. Apakah jenis insurans kesihatan yang anda ada? (Pilih satu atau lebih)

- ◯ Insurans Kesihatan Kebangsaan
- ◯ Insurans Kesihatan Pekerja
- ◯ Insurans Kesihatan Swasta
- ◯ Insurans Kesihatan Pelancongan
- ◯ Tiada insurans kesihatan
- ◯ Memilih untuk tidak menjawab

12. Berapa lamakah anda telah menetap di Jepun?

- kurang dari 1 tahun
- 1-2 tahun
- 3-5 tahun
- 6-10 tahun
- lebih dari 10 tahun
- Memilih untuk tidak menjawab

13. Apakah tahap kefasihan Bahasa Jepun anda?

- Saya boleh bertutur fasih, sama seperti orang Jepun.
- Bahasa Jepun saya cukup fasih untuk berkerja and belajar.
- Bahasa Jepun saya cukup fasih untuk memastikan tiada masalah dalam kehidupan harian.
- Saya tidak boleh bertutur dengan fasih.
- Saya tidak boleh bertutur langsung.
- Memilih untuk tidak menjawab.

14. Jumlah orang yang tinggal di rumah/ bilik anda (termasuk anda).

▢ 1
▢ 2
▢ 3
▢ 4
▢ 5 atau lebih

15. Jumlah orang dewasa yang berumur 65 tahun dan ke atas, yang tinggal bersama anda.

▢ 0
▢ 1
▢ 2
▢ 3
▢ 4
▢ 5 atau lebih

16. Dimanakah ahli keluarga anda yang lain (ibu bapa, adik-beradik) menetap sekarang? (Pilih satu atau lebih)

▢ Saya tidak mepunyai ahli keluarga yang lain.
▢ Bersama saya di Jepun.
▢ Rumah lain di Jepun.
▢ Negara asal.
▢ Negara lain.
▢ Saya tidak tahu.
▢ Memilih untuk tidak menjawab.

17. Bilakah kali terakhir anda menjumpai ahli keluarga (ibu, bapa,adik-beradik) anda?

▢ Kurang daripada 1 tahun
▢ 1-2 tahun
▢ 3-5 tahun
▢ 6-10 tahun
▢ Lebih daripada 10 tahun
▢ Memilih untuk tidak menjawab

18. Berapa kerap anda menelefon ahli keluarga anda (ibu, bapa, adik-beradik)?

▢ Setiap hari
▢ Setiap minggu
▢ Setiap bulan
▢ Beberapa kali dalam setahun
▢ Saya tidak ingat
▢ Tidak pernah
▢ Memilih untuk tidak menjawab

BAHAGIAN 2: Rangkaian Informasi

19. Adakah anda mempunyai sesiapa sahaja untuk berkongsi masalah anda?

◯ Ya
◯ Tidak
◯ Memilih untuk tidak menjawab

20. Dengan siapakah anda boleh berkongsi masalah anda? (Pilih satu atau lebih)

☐ Ibu saya
☐ Ayah saya
☐ Adik perempuan/ kakak saya
☐ Adik lelaki/ abang saya
☐ Sepupu saya
☐ Saudara-mara lain
☐ Guru
☐ Rakan sekerja
☐ Rakan Jepun
☐ Rakan di negara asal saya
☐ Agamawan
☐ Perunding kerajaan
☐ Memilih untuk tidak menjawab
☐ Lain-lain (Sila nyatakan)

21. Dari manakah anda memperoleh maklumat mengenai COVID-19? (Pilih satu atau lebih)

☐ Ahli keluarga di negara asal anda
☐ Ahli keluarga di Jepun
☐ Rakan Jepun
☐ Rakan dari negara yang sama.
☐ Organisasi, kemudahan, atau kedai dimana individu dari negara yang sama berkumpul.
☐ Majlis perbandaran dan wilayah
☐ Majalah informasi orang awam dan laman sesawang majlis perbandaran dan wilayah.
☐ NPO pertukaran antarabangsa.
☐ TV, surat khabar, majalah, atau Internet
☐ SNS (Twitter, Facebook, Instagram, and lain-lain)
☐ Saya tidak tahu bagaimana untuk mendapatkan maklumat.
☐ Memilih untuk tidak menjawab
☐ Lain-lain (Sila nyatakan)

22. Apakah jenis maklumat yang anda mahu dalam bahasa anda? (Pilih satu atau lebih)

▢ Apa yang perlu dilakukan sekiranya saya disyaki mempunyai simptom-simptom COVID-19
▢ Apa yang perlu dilakukan sekiranya penyakit atau kecederaan selain covid-19 dalam tempoh COVID-19 ini.
▢ Apa yang perlu dilakukan sekiranya berlaku bencana.
▢ Apa yang perlu dilakukan semasa pergi ke hospital/ klinik.
▢ Apakah yang perlu dilakukan sekiranya saya mempunyai masalah dengan jiran, keganasan rumah tangga, atau gangguan seksual.
▢ Informasi mengenai melahirkan anak.
▢ Apakah yang perlu dilakukan sekiranya anak saya mempunyai masalah.
▢ Pendidikan, sekolah untuk kanak-kanak.
▢ Kawalan jangkitan COVID-19.
▢ Dimanakah kawasan "Hotspot" COVID-19.
▢ Langkah-langkah pencegahan COVID-19.
▢ Bantuan kewangan berkaitan COVID-19.
▢ Bagaimana untuk kembali ke negara asal.
▢ Bagaimana untuk melanjutkan tempoh visa.
▢ Bagaimana untuk menelefon ahli keluarga saya dari Jepun.
▢ Pengangkutan awam.
▢ Bagaimana untuk mendapatkan lesen memandu.
▢ Sistem cukai dan pencen
▢ Acara Komuniti
▢ Pengalaman mencari rumah untuk didiami
▢ Maklumat pekerjaan dan pengalaman mencarinya.
▢ Bagaimana untuk membeli insurans kebangsaan/ swasta.
▢ Memilih untuk tidak menjawab
▢ Lain-lain (Sila nyatakan)

BAHAGIAN 3: Jawapan pendek

23. Adakah baru-baru ini?

ahli keluarga anda disahkan positif COVID-19?

○ Ya ○ Tidak ○ Memilih untuk tidak menjawab

ahli keluarga anda disyaki mempunyai jangkitan COVID-19 tetapi tidak membuat saringan?

○ Ya ○ Tidak ○ Memilih untuk tidak menjawab

anda telah mempunyai masalah/ kesukaran dalam pembelajaran atau kerja?

○ Ya ○ Tidak ○ Memilih untuk tidak menjawab

berasa diskriminasi disebabkan oleh status bukan orang Jepun?

○ Ya ○ Tidak ○ Memilih untuk tidak menjawab

anda kehilangan pekerjaan?

○ Ya ○ Tidak ○ Memilih untuk tidak menjawab

anda mengalami keganasan rumah tangga?

○ Ya ○ Tidak ○ Memilih untuk tidak menjawab

anda mengalami gangguan seksual?

○ Ya ○ Tidak ○ Memilih untuk tidak menjawab

anda merasai anda lebih aktif dalam aspek keagamaan?

○ Ya ○ Tidak ○ Memilih untuk tidak menjawab

BAHAGIAN 4: PERUBAHAN DALAM KEHIDUPAN PERIBADI

24. Nyatakan perasaan dan mood anda baru-baru ini (atau sekarang)?

Saya rasa tenang
○Tidak langsung ○Sedikit ○Sederhana ○Sangat

Saya rasa tertekan
○Tidak langsung ○Sedikit ○Sederhana ○Sangat

Saya rasa sedih
○Tidak langsung ○Sedikit ○Sederhana ○Sangat

Saya rasa relaks
○Tidak langsung ○Sedikit ○Sederhana ○Sangat

Saya berpuas hati
○Tidak langsung ○Sedikit ○Sederhana ○Sangat

Saya rasa risau
○Tidak langsung ○Sedikit ○Sederhana ○Sangat

I rasa sepi
○Tidak langsung ○Sedikit ○Sederhana ○Sangat

I rasa terasing
○Tidak langsung ○Sedikit ○Sederhana ○Sangat

Saya takut dijangkiti COVID-19
○Tidak langsung ○Sedikit ○Sederhana ○Sangat

25. TAHAP MASALAH ANDA

Durasi tidur

- ◯ Kurang daripada sebelum ini
- ◯ Sama seperti sebelum ini
- ◯ Lebih daripada sebelum ini
- ◯ Tidak berkenaan
- ◯ Saya memilih untuk tidak menjawab

Berat badan

- ◯ Kurang daripada sebelum ini
- ◯ Sama seperti sebelum ini
- ◯ Lebih daripada sebelum ini
- ◯ Tidak berkenaan
- ◯ Saya memilih untuk tidak menjawab

Pengambilan alkohol

- ◯ Kurang daripada sebelum ini
- ◯ Sama seperti sebelum ini
- ◯ Lebih daripada sebelum ini
- ◯ Tidak berkenaan
- ◯ Saya memilih untuk tidak menjawab

Merokok

- ◯ Kurang daripada sebelum ini
- ◯ Sama seperti sebelum ini
- ◯ Lebih daripada sebelum ini
- ◯ Tidak berkenaan
- ◯ Saya memilih untuk tidak menjawab

Bermain permainan/meluangkan masa dengan telefon pintar/ TV

- ◯ Kurang daripada sebelum ini
- ◯ Sama seperti sebelum ini
- ◯ Lebih daripada sebelum ini
- ◯ Tidak berkenaan
- ◯ Saya memilih untuk tidak menjawab

Aktiviti fizikal/ melakukan senaman

- ◯ Kurang daripada sebelum ini
- ◯ Sama seperti sebelum ini
- ◯ Lebih daripada sebelum ini

- Tidak berkenaan
- Saya memilih untuk tidak menjawab

Durasi menjaga anak-anak
- Kurang daripada sebelum ini
- Sama seperti sebelum ini
- Lebih daripada sebelum ini
- Tidak berkenaan
- Saya memilih untuk tidak menjawab

Konflik/ pertengkaran di rumah anda
- Kurang daripada sebelum ini
- Sama seperti sebelum ini
- Lebih daripada sebelum ini
- Tidak berkenaan
- Saya memilih untuk tidak menjawab

Komunikasi dengan jiran dan rakan-rakan
- Kurang daripada sebelum ini
- Sama seperti sebelum ini
- Lebih daripada sebelum ini
- Tidak berkenaan
- Saya memilih untuk tidak menjawab

Kesihatan mental anda secara keseluruhan
- Kurang daripada sebelum ini
- Sama seperti sebelum ini
- Lebih daripada sebelum ini
- Tidak berkenaan
- Saya memilih untuk tidak menjawab

Kesihatan fizikal anda secara keseluruhan
- Kurang daripada sebelum ini
- Sama seperti sebelum ini
- Lebih daripada sebelum ini
- Tidak berkenaan
- Saya memilih untuk tidak menjawab

Kesihatan emosi anda secara keseluruhan (kegembiraan)
- Kurang daripada sebelum ini
- Sama seperti sebelum ini

◯ Lebih daripada sebelum ini
◯ Tidak berkenaan
◯ Saya memilih untuk tidak menjawab

Kepuasan seksual anda secara keseluruhan
◯ Kurang daripada sebelum ini
◯ Sama seperti sebelum ini
◯ Lebih daripada sebelum ini
◯ Tidak berkenaan
◯ Saya memilih untuk tidak menjawab

Pendapatan sendiri
◯ Kurang daripada sebelum ini
◯ Sama seperti sebelum ini
◯ Lebih daripada sebelum ini
◯ Tidak berkenaan
◯ Saya memilih untuk tidak menjawab

Kestabilan kewangan rumah tangga anda secara keseluruhan
◯ Kurang daripada sebelum ini
◯ Sama seperti sebelum ini
◯ Lebih daripada sebelum ini
◯ Tidak berkenaan
◯ Saya memilih untuk tidak menjawab

26. Bilangan kanak-kanak di bawah umur 18 tahun yang tinggal bersama anda.

- ◯ 0
- ◯ 1
- ◯ 2
- ◯ 3
- ◯ 4
- ◯ Lebih daripada 4

27. TAHAP MASALAH ANAK-ANAK ANDA

Tempoh tidur anak-anak

- ◯ Kurang daripada sebelum ini
- ◯ Sama seperti sebelum ini
- ◯ Lebih daripada sebelum ini
- ◯ Tidak berkenaan
- ◯ Saya memilih untuk tidak menjawab

Waktu anak-anak bermain /meluangkan masa dengan telefon pintar/TV

- ◯ Kurang daripada sebelum ini
- ◯ Sama seperti sebelum ini
- ◯ Lebih daripada sebelum ini
- ◯ Tidak berkenaan
- ◯ Saya memilih untuk tidak menjawab

Aktiviti fizikal/ senaman anak-anak

- ◯ Kurang daripada sebelum ini
- ◯ Sama seperti sebelum ini
- ◯ Lebih daripada sebelum ini
- ◯ Tidak berkenaan
- ◯ Saya memilih untuk tidak menjawab

Kemarahan anak-anak

- ◯ Kurang daripada sebelum ini
- ◯ Sama seperti sebelum ini
- ◯ Lebih daripada sebelum ini
- ◯ Tidak berkenaan
- ◯ Saya memilih untuk tidak menjawab

Komunikasi anak-anak bersama rakan mereka

- ◯ Kurang daripada sebelum ini
- ◯ Sama seperti sebelum ini
- ◯ Lebih daripada sebelum ini
- ◯ Tidak berkenaan
- ◯ Saya memilih untuk tidak menjawab

Komunikasi anak-anak bersama ahli keluarga

- ◯ Kurang daripada sebelum ini
- ◯ Sama seperti sebelum ini
- ◯ Lebih daripada sebelum ini

- Tidak berkenaan
- Saya memilih untuk tidak menjawab

Kehidupan persekolahan anak-anak
- Kurang daripada sebelum ini
- Sama seperti sebelum ini
- Lebih daripada sebelum ini
- Tidak berkenaan
- Saya memilih untuk tidak menjawab

Kesihatan mental anak-anak secara keseluruhan
- Kurang daripada sebelum ini
- Sama seperti sebelum ini
- Lebih daripada sebelum ini
- Tidak berkenaan
- Saya memilih untuk tidak menjawab

Kesihatan fizikal anak-anak secara keseluruhan
- Kurang daripada sebelum ini
- Sama seperti sebelum ini
- Lebih daripada sebelum ini
- Tidak berkenaan
- Saya memilih untuk tidak menjawab

Kesihatan emosi anak-anak secara keseluruhan (kegembiraan)
- Kurang daripada sebelum ini
- Sama seperti sebelum ini
- Lebih daripada sebelum ini
- Tidak berkenaan
- Saya memilih untuk tidak menjawab

28. Masalah lain (Sila nyatakan di ruangan di bawah)

Sila hubungi kami sekiranya anda ingin berunding secara terus dengan ahli NPO (Email: info@universalaid.jp; Tel: 090-2900-3934). Kami ingin memberikan sokongan yang diperlukan bagi pemastautin bukan Jepun agar dapat menjalani kehidupan yang selesa, walaupun dalam situasi COVID-19 ini.

Terima kasih atas kerjasama anda.

जापानमा बसोबास गर्ने गैर-जापानी बासिन्दाहरूको कोभिड-१९ स्थितिको अवस्थामा भएका समस्या र चिन्ताहरू : एक क्रस-अनुभागीय सर्वेक्षण

हामी, UNIVERSALAID.JP र नागासाकी विश्वविध्यालय , जापानमा (विशेष गरी नागासाकी शहरमा) बसोबास गर्ने गैर-जापानी निवासीहरूमा कोभिड-१९ महामारीको कारणले भएका समस्या र चिन्ता पत्ता लगाउन यो सर्वेक्षण गरिरहेका छौं, समस्या र चिन्ता निवारणको लागी सुझाव र परामर्श प्रदान गर्ने प्रयासमा ।

सर्वेक्षणमा जम्मा २८ वटा प्रश्नहरू छन् र यसले करीव ७-१० मिनेट लिन सक्दछ। तपाईं कुनै पनि समय सहभागी हुन अस्वीकार गर्न वा भाग लिन बाट हट्न स्वतन्त्र हुनुहुन्छ। यस सर्वेक्षणमा संकलन गरिएका प्रतिक्रियाहरू गोप्य राखिने छन् र कुनै पनि हालतमा जारी गरिने छैनन्। थप, यस सर्वेक्षण पूर्ण रुपमा अज्ञात रहने छ र यससंग कुनै जोखिम सम्बन्धित छैन।

कृपया URL वा QR कोड अनुसरण गरेर सर्वेक्षणमा भाग लिनुहोस् ।
https://universalaid.jp/survey2020/

कुनै पनि थप प्रश्नहरूको लागि तपाईंले हाम्रो टोलिसंग सम्पर्क गर्न सुश्री योशमी मत्सुओ, UNIVERSALAID.JP (ईमेल: info@universalaid.jp; फोन: ०९०-२९००-३९३४) अथवा डा. नुग्वेन टियन ह्यु, नागासाकी विश्वविध्यालयलाई, ईमेल गर्न सक्नुहुन्छ। (ईमेल: tienhuy@nagasaki-u.ac.jp)

यो प्रश्नावली पूरा गरेर बुझाई, तपाईं सर्वेक्षणमा भाग लिनको लागि आफ्नो सहमति जनाउँदै हुनुहुन्छ।

१. के तपाईं १८ बर्ष भन्दा माथिको हुनुहुन्छ र यस अनुसन्धान परियोजनामा भाग लिन सहमत हुनुहुन्छ?

○ छ

○ छैन

खण्ड १ : सामान्य जानकारी

२. तपाईंको जन्म भएको देश कुन हो?

○ जवाफ दिन चाहन्न

३. तपाईं कहाँ बस्नुहुन्छ?

○ नागासाकी शहर
○ नागासाकी प्रान्तका अन्य शहरहरू
○ अन्य प्रान्तहरू
○ जवाफ दिन चाहन्न

४. तपाईंको उमेर दायरा (बर्ष)

१८-२४ वर्ष
○ २५-३४
○ ३५-४४
○ ४५-५४
○ ५५-६४
○ ६५ वर्ष वा सो भन्दा बढी
○ जवाफ दिन चाहन्न

५. लिंग

○ पुरुष
○ महिला
○ जवाफ दिन चाहन्न
○ अन्य (कृपया निर्दिष्ट गर्नुहोस्)

६. वैवाहिक स्थिति

- ○ अविवाहित/ कहिल्यै विवाह गरेको छैन
- ○ विवाहित वा घरेलु साझेदारी
- ○ विधवा
- ○ सम्बन्धविच्छेद
- ○ छुट्टिएको
- ○ जवाफ दिन चाहन्न

७. तपाईंको जीवनसाथी / साझेदारको मूल नागरिकता के हो?

- ○ जापानी
- ○ आफ्नो जस्तै नागरिकता
- ○ मसँग जीवनसाथी / साझेदार छैन
- ○ जवाफ दिन चाहन्न
- ○ अन्य (कृपया निर्दिष्ट गर्नुहोस्)

८. काम / पेशा (एक वा बढी चयन गर्नुहोस्)

- ○ पूर्ण-समय कर्मचारी
- ○ अंशकालिक कर्मचारी / अनौपचारिक रोजगार
- ○ स्वरोजगार
- ○ गृहिणी / पति
- ○ बेरोजगार
- ○ विद्यार्थी
- ○ सेवानिवृत्त
- ○ जवाफ दिन चाहन्न
- ○ अन्य (कृपया निर्दिष्ट गर्नुहोस्)

९. शिक्षा

○ पोस्ट-ग्रेजुएशन वा उच्च
○ कलेज / विश्वविद्यालय डिग्री
○ उच्च माध्यमिक बिद्यालय
○ निम्न माध्यमिक बिद्यालय
○ प्राथमिक बिद्यालय
○ कुनै स्कूल गएको छैन
○ जवाफ दिन चाहन्न

१०. अध्यागमन स्थिति

○ प्रक्रितिकीकृत
○ स्थायी निवासी
○ कार्य अनुमति
○ विद्यार्थी भिसा
○ परिवार निर्भर भिसा
○ अस्थायी भिसा (पर्यटक, व्यवसाय)
○ जवाफ दिन चाहन्न
○ अन्य (कृपया निर्दिष्ट गर्नुहोस्)

११. तपाईंसँग कस्तो प्रकारको स्वास्थ्य बीमा छ? (एक वा बढी चयन गर्नुहोस्)
○ राष्ट्रिय स्वास्थ्य बीमा
○ कर्मचारी स्वास्थ्य बीमा
○ निजी स्वास्थ्य बीमा
○ यात्रा स्वास्थ्य बीमा
○ कुनै पनि छैन
○ जवाफ दिन चाहन्न

१२. तपाईं कहिलेदेखि जापानमा बस्दै हुनुहुन्छ?
○ १ बर्ष भन्दा कम
○ १-२ बर्ष
○ ५-६ बर्ष
○ ६-१० वर्ष
○ १० बर्ष भन्दा बढी
○ जवाफ दिन चाहन्न

१३. तपाईंको जापानी भाषा कुन स्तरको छ?

○ म जापानी मानिसहरु जस्तै समान स्तरमा बोल्न सक्छु।

○ म काम गर्न वा अध्ययन को लागी पर्याप्त बोल्न सक्छु।

○ दैनिक जीवनमा कुनै समस्या नपर्ने गरि म राम्ररी बोल्न सक्छु।

○ म जापानीमा धेरै राम्रोसँग बोल्न सक्दिन ।

○ म जापानी पटक्कै बोल्न सक्दिन ।

○ जवाफ दिन चाहन्न

१४. तपाईंको घर / कोठामा बस्ने व्यक्तिहरूको जम्मा संख्या (आफू सहित)।

▢ १

▢ २

▢ ३

▢ ४

▢ ५ वा अधिक

१५. तपाईंसँग बस्नु हुने ६५ बर्ष वा माथिका वयस्कहरूको संख्या।

○ ०

○ १

○ २

○ ३

○ ४

○ ५ वा अधिक

१६. परिवारका अन्य सदस्यहरू (आमा बुबा, बहिनीहरू, भाइहरू) अहिले कहाँ बस्छन्? (एक वा बढी चयन गर्नुहोस्)

- मसँग परिवारका अन्य सदस्यहरू छैनन्
- आफू संगै जापानमा
- जापानको अन्य घरमा
- स्वदेशमा
- अन्य देशहरूमा
-मलाई थाहा छैन
-जवाफ दिन चाहन्न

१७. अन्तिम पटक कहिले तपाईंले आफ्नो परिवारका अन्य सदस्यहरूसँग (आमा बुबा, बहिनी, दाजुभाइहरू) भेटघाट गर्नुभयो?

○ १ बर्ष भन्दा कम
○ १-२ बर्ष
○ ५-६ बर्ष
○ ६-१० वर्ष
○ १० बर्ष भन्दा बढी
○ जवाफ दिन चाहन्न

१८. तपाईं आफ्नो परिवारका सदस्यहरूलाई कति पटक फोन गर्नुहुन्छ (आमा बुबा, बहिनी, दाजुभाइहरू)?

○ हरेक दिन
○ हरेक हप्ता
○ हरेक महिना
○ बर्षमा कहिलेकाहिँ
○ सम्झना छैन
○ कहिले पनि गर्दिन
○ जवाफ दिन चाहन्न

खण्ड २: सूचना संजाल

१९. आफ्नो समस्याहरू बाँड्न तपाईंसँग कोहि छ ?
○ छ
○ छैन
○ जवाफ दिन चाहन्न

२०. तपाईं कोसँग आफ्नो समस्याहरु बाँड्न सक्नुहुन्छ? (एक वा बढी चयन गर्नुहोस्)

- आफ्नो आमा
- आफ्नो बुबा
- आफ्नो बहिनी
- आफ्नो भाई
- आफ्नो दाई/भाई वा दिदी/बहिनी पर्ने
- अन्य आफन्तहरू
- शिक्षक
- सहकर्मीहरू
- जापानी मित्र
- स्वदेशी मित्र
- धार्मिक नेता
- सरकारी परामर्शदाता
- जवाफ दिन चाहन्न
- अन्य (कृपया निर्दिष्ट गर्नुहोस्)

२१. कोभिड-१९ को बारेमा जानकारी तपाईंले कहाँबाट पाउनुहुन्छ? (एक वा बढी चयन गर्नुहोस्)

- स्वदेशमा भएका परिवार
-जापानमा भएका परिवार
-जापानी साथीहरु
-स्वदेशी साथीहरु
- संगठन, सुविधा वा स्टोरहरू जहाँ एकै देशका मानिसहरु भेला हुन्छन
- नगरपालिका र प्रान्तीय कार्यालय
- सार्वजनिक सूचना पत्रिकाहरू र नगरपालिका र प्रान्तहरूका वेबसाइटहरू
- अन्तर्राष्ट्रिय एक्सचेन्ज एनपीओ
- टिभी, समाचार पत्र, म्यागजिन, वा इन्टरनेट
- SNS (ट्विटर, फेसबुक, इन्स्टाग्राम, र यस्तै)
- मलाई जानकारी प्राप्त गर्ने तरिका थाहा छैन
- जवाफ दिन चाहन्न
- अन्य (कृपया निर्दिष्ट गर्नुहोस्)

२२. तपाईं आफ्नो भाषामा कस्तो प्रकारको जानकारी चाहानुहुन्छ? (एक वा बढी चयन गर्नुहोस्)

- आफू सँग कोभिड-१९ संदिग्ध लक्षण भएको अवस्थामा के गर्ने
-   यस कोभिड-१९ अवधिमा, कोभिड-१९ बाहेक अन्य बिरामी वा चोटपटकको अवस्थामा के गर्ने
- प्रकोपको अवस्थामा के गर्ने
- अस्पताल / क्लिनिकहरूमा जाँदा के गर्ने
- यदि मलाई छर-छिमेक, घरेलु हिंसा, वा यौन उत्पीडनको समस्या छ भने के गर्ने
- प्रशुती र बच्चा जन्माउने बारे
- यदि मेरो बच्चामा कुनै समस्या छ भने के गर्ने
- बच्चाहरूको लागि शिक्षा, विद्यालय
- कोभिड-१९ को संक्रमण नियन्त्रण भएको स्थिति
- कहाँ कोभिड-१९ हटस्पटहरू छन्
-कोभिड-१९ रोकथामका उपायहरू
-कोभिड-१९ सम्बन्धि वित्तीय सहयोग
- स्वदेश फिर्ता कसरी जाने
- भिसा कसरी बढाउने
- जापानबाट परिवारलाई कसरी फोन गर्ने
- सार्वजनिक यातायात
- ड्राइविंग इजाजत पत्र कसरी प्राप्त गर्ने
-कर र पेन्सन प्रणाली
- सामुदायिक कार्यक्रमहरु
- बसाईको लागि घर खोज्ने अनुभव
-काम सम्बन्धि जानकारी र रोजगार खोज्दाको अनुभव
- राष्ट्रीय र / वा निजी स्वास्थ्य बीमा कसरी खरीद गर्ने
- जवाफ दिन चाहन्न
- अन्य (कृपया निर्दिष्ट गर्नुहोस्)

खण्ड ३: छोटो प्रश्नहरू

२३. - के तपाईंको/ ले हालसालै

- परिवारको कुनै सदस्यमा कोभिड-१९ पुष्टि भएको छ ?

○ छ

○ छैन

○ जवाफ दिन चाहन्न

- परिवारको कुनै सदस्यमा कोभिड-१९ लक्षण देखा परेको थियो तर परिक्षण गर्न सकिएन ?

○ छ

○ छैन

○ जवाफ दिन चाहन्न

- कुनै कुरा सिक्न वा काम गर्नमा समस्याहरु/कठिनाइहरु थिए ?

○ छ

○ छैन

○ जवाफ दिन चाहन्न

- केवल एक गैर-जापानी भएको कारण भेदभाव गरिएको अनुभव गर्नु भएको थियो?

○ छ

○ छैन

○ जवाफ दिन चाहन्न

- रोजगारी गुमाउनु भयो?

○ छ

○ छैन

○ जवाफ दिन चाहन्न

- घरमा घरेलु हिंसा भएको थियो ?

○ छ

○ छैन

○ जवाफ दिन चाहन्न

-यौन उत्पीडन भएको थियो ?

○ छ

○ छैन

○ जवाफ दिन चाहन्न

- बढी अध्यात्मिक/ धार्मिक गतिविधिमा सक्रिय भएको महसुस गर्नु भयो ?

○ छ

○ छैन

○ जवाफ दिन चाहन्न

खण्ड ४: व्यक्तिगत जीवनमा आएका परिवर्तनहरू

२४ तपाइँको हालसालैको (वा वर्तमानका) भावनाहरू / स्थिति वर्णन गर्नुहोस्?

- म शान्त महसुस गर्छु

  ○ पटक्कै गर्दिन
  ○ केहि हदसम्म
  ○ सामान्य रूपमा
  ○ अत्याधिक

-म तनावपूर्ण छु

  ○ पटक्कै गर्दिन
  ○ केहि हदसम्म
  ○ सामान्य रूपमा
  ○ अत्याधिक

- म निराश छु

  ○ पटक्कै गर्दिन
  ○ केहि हदसम्म
  ○ सामान्य रूपमा
  ○ अत्याधिक

- मैले आराम पाएको छु

  ○ पटक्कै गर्दिन
  ○ केहि हदसम्म
  ○ सामान्य रूपमा
  ○ अत्याधिक

- म सन्तुष्ट छु

  ○ पटक्कै गर्दिन
  ○ केहि हदसम्म

- सामान्य रूपमा
- अत्याधिक

-म चिन्तित छु
- पटक्कै गर्दिन
- केहि हदसम्म
- सामान्य रूपमा
- अत्याधिक

-मैले एक्लो महसुस गरें
- पटक्कै गर्दिन
- केहि हदसम्म
- सामान्य रूपमा
- अत्याधिक

-मैले एक्लिएको महसुस गरें
- पटक्कै गर्दिन
- केहि हदसम्म
- सामान्य रूपमा
- अत्याधिक

-मलाई कोभिड-१९ संक्रमण हुने डर छ
- पटक्कै गर्दिन
- केहि हदसम्म
- सामान्य रूपमा
- अत्याधिक

२५. तपाईका समस्याहरूको स्तर

- निद्रा अवधि
    - ○ पहिले भन्दा कम
    - ○ पहिले जस्तै
    - ○ पहिले भन्दा बढि
    - ○ लागु हुँदैन
    - ○ जवाफ दिन चाहन्न

- शारीरिक तौल
    - ○ पहिले भन्दा कम
    - ○ पहिले जस्तै
    - ○ पहिले भन्दा बढि
    - ○ लागु हुँदैन
    - ○ जवाफ दिन चाहन्न

- मदिरा खपत
    - ○ पहिले भन्दा कम
    - ○ पहिले जस्तै
    - ○ पहिले भन्दा बढि
    - ○ लागु हुँदैन
    - ○ जवाफ दिन चाहन्न

-धुम्रपान
    - ○ पहिले भन्दा कम
    - ○ पहिले जस्तै
    - ○ पहिले भन्दा बढि
    - ○ लागु हुँदैन
    - ○ जवाफ दिन चाहन्न

खेल खेल्दै / स्मार्टफोन / टिभीमा समय व्यतित गर्दै
    - ○ पहिले भन्दा कम
    - ○ पहिले जस्तै
    - ○ पहिले भन्दा बढि
    - ○ लागु हुँदैन
    - ○ जवाफ दिन चाहन्न

- भौतिक गतिविधिहरू / व्यायाम गर्दै

   ○ पहिले भन्दा कम
   ○ पहिले जस्तै
   ○ पहिले भन्दा बढि
   ○ लागु हुँदैन
   ○ जवाफ दिन चाहन्न

- प्यारेन्टिंग वा बाल-स्याहार अवधि

   ○ पहिले भन्दा कम
   ○ पहिले जस्तै
   ○ पहिले भन्दा बढि
   ○ लागु हुँदैन
   ○ जवाफ दिन चाहन्न

- तपाईंको घरमा द्वन्द्व / झगडा

   ○ पहिले भन्दा कम
   ○ पहिले जस्तै
   ○ पहिले भन्दा बढि
   ○ लागु हुँदैन
   ○ जवाफ दिन चाहन्न

- छिमेकीहरू वा साथीहरूसँग संचार

   ○ पहिले भन्दा कम
   ○ पहिले जस्तै
   ○ पहिले भन्दा बढि
   ○ लागु हुँदैन
   ○ जवाफ दिन चाहन्न

- तपाईंको समग्र मानसिक स्वास्थ्य

   ○ पहिले भन्दा कम
   ○ पहिले जस्तै
   ○ पहिले भन्दा बढि
   ○ लागु हुँदैन
   ○ जवाफ दिन चाहन्न

तपाईंको समग्र शारीरिक स्वास्थ्य

   ○ पहिले भन्दा कम

○ पहिले जस्तै
○ पहिले भन्दा बढि
○ लागु हुँदैन
○ जवाफ दिन चाहन्न

- तपाईंको समग्र भावनात्मक स्वास्थ्य (खुशी)
  ○ पहिले भन्दा कम
  ○ पहिले जस्तै
  ○ पहिले भन्दा बढि
  ○ लागु हुँदैन
  ○ जवाफ दिन चाहन्न

- तपाईंको समग्र यौन सन्तुष्टि
  ○ पहिले भन्दा कम
  ○ पहिले जस्तै
  ○ पहिले भन्दा बढि
  ○ लागु हुँदैन
  ○ जवाफ दिन चाहन्न

-व्यक्तिगत आय
  ○ पहिले भन्दा कम
  ○ पहिले जस्तै
  ○ पहिले भन्दा बढि
  ○ लागु हुँदैन
  ○ जवाफ दिन चाहन्न

तपाईंको परिवारको समग्र आर्थिक स्थिरता
  ○ पहिले भन्दा कम
  ○ पहिले जस्तै
  ○ पहिले भन्दा बढि
  ○ लागु हुँदैन
  ○ जवाफ दिन चाहन्न

२६. तपाईं संग बस्ने १८ बर्ष मुनिका बच्चाहरुको संख्या ।

○ ०
○ १

○ २
○ ३
○ ४
○ ५ वा अधिक

२७. तपाईंको बच्चाहरूको समस्याहरूको स्तर

- बच्चाहरूको निद्रा अवधि
    ○ कम / पहिला भन्दा कम
    ○ पहिला जस्तै
    ○ बढी / पहिला भन्दा बढी
    ○ लागु हुँदैन
    ○ जवाफ दिन चाहन्न

- बच्चाहरूको खेलकुद / स्मार्टफोन / टिभीमा समय व्यतित गर्दै
    ○ कम / पहिला भन्दा कम
    ○ पहिला जस्तै
    ○ बढी / पहिला भन्दा बढी
    ○ लागु हुँदैन
    ○ जवाफ दिन चाहन्न

- बच्चाहरूको शारीरिक गतिविधिहरू / व्यायाम गर्दै
    ○ कम / पहिला भन्दा कम
    ○ पहिला जस्तै
    ○ बढी / पहिला भन्दा बढी
    ○ लागु हुँदैन
    ○ जवाफ दिन चाहन्न

- बच्चाहरूको आक्रोश / क्रोध
    ○ कम / पहिला भन्दा कम
    ○ पहिला जस्तै
    ○ बढी / पहिला भन्दा बढी
    ○ लागु हुँदैन
    ○ जवाफ दिन चाहन्न

- बच्चाहरूको साथीहरूसँग संचार
    - कम / पहिला भन्दा कम
    - पहिला जस्तै
    - बढी / पहिला भन्दा बढी
    - लागु हुँदैन
    - जवाफ दिन चाहन्न

- बच्चाहरुको परिवारका सदस्यहरूसँग संचार
    - कम / पहिला भन्दा कम
    - पहिला जस्तै
    - बढी / पहिला भन्दा बढी
    - लागु हुँदैन
    - जवाफ दिन चाहन्न

- बच्चाहरूको स्कूले जीवन
    - कम / पहिला भन्दा कम
    - पहिला जस्तै
    - बढी / पहिला भन्दा बढी
    - लागु हुँदैन
    - जवाफ दिन चाहन्न

- बच्चाहरूको समग्र मानसिक स्वास्थ्य
    - कम / पहिला भन्दा कम
    - पहिला जस्तै
    - बढी / पहिला भन्दा बढी
    - लागु हुँदैन
    - जवाफ दिन चाहन्न

- बच्चाहरूको समग्र शारीरिक स्वास्थ्य
    - कम / पहिला भन्दा कम
    - पहिला जस्तै
    - बढी / पहिला भन्दा बढी
    - लागु हुँदैन
    - जवाफ दिन चाहन्न

- बच्चाहरूको समग्र भावनात्मक स्वास्थ्य खुशी

○ कम / पहिला भन्दा कम

○ पहिला जस्तै

○ बढी / पहिला भन्दा बढी

○ लागु हुँदैन

○ जवाफ दिन चाहन्न

२८. अन्य समस्याहरू (कृपया यहाँ लेख्नुहोस)

यदि तपाईं सिधा हाम्रा एनपीओ सदस्यहरूसँग परामर्श लिन चाहनुहुन्छ भने, कृपया हामीलाई सम्पर्क गर्नुहोस् ।

(ईमेल: info@universalaid.jp; फोन: ०९०-२९००-३९३४)

हामी गैर कोभिड-१९ अवधि अन्तर्गत पनि, आरामदायी जीवन बिताउन गैर-जापानी निवासीहरूलाई आवश्यक सहयोग प्रदान गर्न चाहन्छौं।

तपाईंको आत्मीय सहयोगका लागि अग्रिम धन्यवाद।

Problemas y ansiedades de los residentes no japoneses que viven en Japón y se encuentran en situación de COVID-19: una encuesta transversal

Nosotros, UNIVERSALAID.JP y la Universidad de Nagasaki, estamos realizando esta encuesta para identificar los problemas y ansiedades de los residentes no japoneses que viven en Japón (particularmente en la ciudad de Nagasaki) causados por la pandemia del COVID-19, en un esfuerzo por brindar sugerencias y consultas para solucionar tales problemas y preocupaciones.

La encuesta consta de 28 preguntas y puede tardar aproximadamente entre 7 y 10 minutos. Usted es libre de rechazar o retirarse de la participación en cualquier momento. Las respuestas recopiladas de esta encuesta son confidenciales y no se revelarán bajo ninguna condición. Además, la encuesta será completamente anónima y no habrá ningún riesgo asociado.

Participe en la encuesta siguiendo la URL o el código QR. https://universalaid.jp/survey2020/

Para cualquier consulta adicional, puede ponerse en contacto con nuestro equipo enviando un correo electrónico a la Sra. Yoshimi Matsuo, UNIVERSALAID.JP (Correo electrónico: info@universalaid.jp ; Tel: 090-2900-3934) o al Dr. Nguyen Tien Huy, Universidad de Nagasaki (Correo electrónico: tienhuy@nagasaki-u.ac.jp).

Al completar y enviar esta encuesta, usted está afirmando su consentimiento para participar en el estudio.

1. ¿Tiene más de 18 años y acepta participar en este proyecto de investigación?

   ○ SI
   ○ NO

SECCIÓN 1: INFORMACIONES GENERALES

2. ¿Cuál es su país de origen?

☐ Prefiero no responder

3. ¿Dónde se encuentra viviendo actualmente?

◯ Ciudad de Nagasaki
◯ Otras ciudades de la prefectura de Nagasaki
◯ Otras prefecturas
◯ Prefiero no responder

4. Su rango de edad (años)

- 18-24 años
- 25-34 años
- 35-44 años
- 45-54 años
- 55-64 años
- 65 años o más
- Prefiero no responder

5. Género

- Masculino
- Femenino
- Prefiero no responder
    - Otro (por favor especifique)

6. Estado civil

- ◯ Soltero, nunca casado
- ◯ Casado o pareja de hecho
- ◯ Viudo/a
- ◯ Divorciado/a
- ◯ Separado/a
- ◯ Prefiero no responder

7. ¿Cuál es la nacionalidad de origen de su cónyuge / pareja?

- ◯ Japonés/a
- ◯ Misma nacionalidad que yo
- ◯ No tengo esposo(a)/ pareja
- ◯ Prefiero no responder
- ◯ Otro/a

8. Trabajo / Ocupación (seleccione uno o más)

- ◯ Empleado/a de tiempo completo
- ◯ Empleado/a a tiempo parcial / empleo ocasional
- ◯ trabajador/a independiente
- ◯ Ama/o de casa / esposo/a
- ◯ Desempleado/a
- ◯ Estudiante
- ◯ Retirado/a
- ◯ Prefiero no responder
- ◯ Otro (por favor especifique)

9. Educación

- ◯ Posgrado o superior
- ◯ Título universitario
- ◯ Secundaria superior
- ◯ Escuela secundaria media
- ◯ Escuela primaria
- ◯ Ningún centro educativo
- ◯ Prefiero no responder

10. Estado migratorio

- Naturalizado
- Residente permanente
- Permiso de trabajo
- Visa de estudiante
- Visa de dependiente familiar
- Visa temporal (turistas, negocios)
- Prefiero no responder
- Otro (por favor especifique)

11. ¿Qué tipo de seguro médico tiene? (seleccione uno o más)

- Seguro Nacional de Salud
- Seguro de salud de los empleados
- Seguro médico privado
- Seguro médico de viaje
- Ninguno
- Prefiero no responder

12. ¿Cuánto tiempo llevas viviendo en Japón?

- ◯ Menos de 1 año
- ◯ 1-2 años
- ◯ 3-5 años
- ◯ 6-10 años
- ◯ Más de 10 años
- ◯ Prefiero no responder

13. ¿Cuál es su nivel de japonés?

- ◯ Puedo hablar al mismo nivel que los japoneses.
- ◯ Puedo hablar lo suficientemente bien para trabajar o estudiar.
- ◯ Puedo hablar lo suficientemente bien como para no tener problemas en la vida diaria.
- ◯ No puedo hablar muy bien en japonés.
- ◯ No puedo hablar japonés en absoluto.
- ◯ Prefiero no responder

14. El número total de personas que viven en su hogar / habitación (incluido usted).

▢ 1
▢ 2
▢ 3
▢ 4
▢ 5 o más

15. Número de adultos de 65 años o mayores que vivan con usted.

▢ 0
▢ 1
▢ 2
▢ 3
▢ 4
▢ 5 o más

16. ¿Dónde residen sus otros miembros de la familia (padres, hermanas, hermanos) actualmente? (seleccione uno o más)

▢ No tengo otros miembros de la familia.
▢ Conmigo en Japón.
▢ En otra casa en Japón
▢ País de origen
▢ Otros países
▢ No lo sé
▢ Prefiero no responder

17. ¿Cuándo fue la última vez que estuvo con sus otros miembros de su familia en persona (padres, hermana, hermanos)?

▢ Menos de 1 año
▢ 1-2 años
▢ 3-5 años
▢ 6-10 años
▢ Mas de 10 años
▢ Prefiero no responder

18. ¿Con qué frecuencia llama a los miembros de su familia (padres, hermanas, hermanos)?

▢ Todos los días
▢ Cada semana
▢ Cada mes
▢ Pocas veces al año
▢ No recuerdo
▢ Nunca
▢ Prefiero no responder

SECCIÓN 2: RED DE INFORMACIÓN

19. ¿Tiene alguien con quien compartir sus problemas personales?

◯ Si
◯ No
◯ Prefiero no responder.

20. ¿Con quién puede compartir sus problemas? (seleccione uno o más)

☐ mi madre
☐ mi padre
☐ mi hermana
☐ mi hermano
☐ mi primo
☐ otros parientes
☐ profesor/a
☐ colegas
☐ Amigos Japoneses
☐ Amigos de mi país de origen
☐ Líder religioso
☐ Consultor/a gubernamental
☐ Prefiero no responder.
☐ Otro (por favor especifique)

21. ¿De dónde obtiene información sobre el COVID-19? (seleccione uno o más)

☐ Familia en tu país
☐ Familia en Japón
☐ Amigos japoneses
☐ Amigos del mismo país
☐ Organizaciones, instalaciones o tiendas donde se reúnen personas de un mismo país
☐ Oficina municipal y prefectural
☐ Revistas de información pública y sitios web de municipios y prefecturas
☐ NPO de intercambio internacional
☐ TV, periódico, revistas o Internet
☐ SNS (Twitter, Facebook, Instagram, etc.)
☐ No sé cómo obtener información
☐ Prefiero no responder
☐ Otro (por favor especifique)

22. ¿Qué tipo de información desea en su idioma? (seleccione uno o más)

▢ Qué hacer en caso de que sospechara que tengo un síntoma de COVID-19
▢ Qué hacer en caso de tener alguna enfermedad o lesiones distintas al COVID-19, en este período de pandemia del COVID-19
▢ Qué hacer en caso de desastre
▢ Qué hacer cuando se trata de ir a hospitales / clínicas
▢ Qué hacer en caso de que tenga problemas con los vecinos, violencia doméstica o acoso sexual
▢ Parto y maternidad
▢ Que hacer en caso de que mi hijo/a tenga un problema
▢ Educación, escuelas para niños
▢ La situación del control de infecciones del COVID-19
▢ Dónde están los puntos de mayor concentración de COVID-19
▢ Medidas preventivas contra el COVID-19
▢ Asistencia financiera relacionada con COVID-19
▢ Cómo volver al país de origen
▢ Cómo extender una visa
▢ Cómo llamar a familiares desde Japón
▢ Transportes públicos
▢ Cómo obtener una licencia de conducir
▢ El sistema tributario y de pensiones
▢ Eventos comunitarios
▢ Experiencias buscando una casa para vivir
▢ Información laboral y experiencias en busca de trabajo
▢ Cómo contratar un seguro médico nacional y / o privado
▢ Prefiero no responder.
▢ Otro (por favor especifique)

SECCIÓN 3: PREGUNTAS BREVES

23. ¿Ha tenido recientemente

un familiar con COVID-19 confirmado?

○ Si ○ No ○ Prefiero no responder

un familiar con sospecha de infección por COVID-19 pero no pudo hacerse una prueba?

○ Si ○ No ○ Prefiero no responder

problemas / dificultades con el aprendizaje o de trabajo?

○ Si ○ No ○ Prefiero no responder

episodios de discriminación por simplemente ser no japonés?

○ Si ○ No ○ Prefiero no responder

algún despido laboral?

○ Si ○ No ○ Prefiero no responder

violencia doméstica en su hogar?

○ Si ○ No ○ Prefiero no responder

un acoso sexual?

○ Si ○ No ○ Prefiero no responder

más actividad espiritual / religiosa?

○ Si ○ No ○ Prefiero no responder

SECCIÓN 4: CAMBIOS EN LA VIDA PERSONAL

24. Describa sus sentimientos / estados de ánimo recientes (o actuales).

Me siento cómodo/a (estoy a gusto)
◯ De ningún modo ◯ Algo ◯ Moderadamente ◯ Mucho

Me siento angustiado/a
◯ De ningún modo ◯ Algo ◯ Moderadamente ◯ Mucho

Me siento disgustado
◯ De ningún modo ◯ Algo ◯ Moderadamente ◯ Mucho

Me siento relajado/a
◯ De ningún modo ◯ Algo ◯ Moderadamente ◯ Mucho

Me siento contento/a
◯ De ningún modo ◯ Algo ◯ Moderadamente ◯ Mucho

Me siento preocupado
◯ De ningún modo ◯ Algo ◯ Moderadamente ◯ Mucho

Me siento solo/a
◯ De ningún modo ◯ Algo ◯ Moderadamente ◯ Mucho

Me siento aislado/a
◯ De ningún modo ◯ Algo ◯ Moderadamente ◯ Mucho

Tengo miedo de contraer la infección por COVID-19.
◯ De ningún modo ◯ Algo ◯ Moderadamente ◯ Mucho

25. NIVEL DE SUS PROBLEMAS

Duración del sueño
◯ Menos que antes ◯ Lo mismo que antes ◯ Más que antes ◯ No aplicable ◯ Prefiero no contestar

Peso corporal
◯ Menos que antes ◯ Lo mismo que antes ◯ Más que antes ◯ No aplicable ◯ Prefiero no contestar

Consumo de alcohol
◯ Menos que antes ◯ Lo mismo que antes ◯ Más que antes ◯ No aplicable ◯ Prefiero no contestar

Fumar
◯ Menos que antes ◯ Lo mismo que antes ◯ Más que antes ◯ No aplicable ◯ Prefiero no contestar

Jugar videojuegos / pasar tiempo en un teléfono inteligente / TV
◯ Menos que antes ◯ Lo mismo que antes ◯ Más que antes ◯ No aplicable ◯ Prefiero no contestar

Actividades físicas / hacer ejercicio
◯ Menos que antes ◯ Lo mismo que antes ◯ Más que antes ◯ No aplicable ◯ Prefiero no contestar

Duración de la crianza o el cuidado de los hijos
◯ Menos que antes ◯ Lo mismo que antes ◯ Más que antes ◯ No aplicable ◯ Prefiero no contestar

Conflicto / disputa en su hogar
◯ Menos que antes ◯ Lo mismo que antes ◯ Más que antes ◯ No aplicable ◯ Prefiero no contestar

Comunicación con vecinos o amigos
◯ Menos que antes ◯ Lo mismo que antes ◯ Más que antes ◯ No aplicable ◯ Prefiero no contestar

Tu salud mental en general
◯ Menos que antes ◯ Lo mismo que antes ◯ Más que antes ◯ No aplicable ◯ Prefiero no contestar

Tu salud física general
◯ Menos que antes ◯ Lo mismo que antes ◯ Más que antes ◯ No aplicable ◯ Prefiero no contestar

Tu salud emocional general (felicidad)
◯ Menos que antes ◯ Lo mismo que antes ◯ Más que antes ◯ No aplicable ◯ Prefiero no contestar

Tu satisfacción sexual general
◯ Menos que antes ◯ Lo mismo que antes ◯ Más que antes ◯ No aplicable ◯ Prefiero no contestar

Ingreso personal

◯ Menos que antes ◯ Lo mismo que antes ◯ Más que antes ◯ No aplicable ◯ Prefiero no contestar

Estabilidad financiera general de su hogar

◯ Menos que antes ◯ Lo mismo que antes ◯ Más que antes ◯ No aplicable ◯ Prefiero no contestar

26. Número de niños menores de 18 años que viven con usted.

- ○ 0
- ○ 1
- ○ 2
- ○ 3
- ○ 4
- ○ Mas de 4

27. NIVEL DE LOS PROBLEMAS DE SUS HIJOS

Duración del sueño de los niños

◯ Más bajo / menos que antes ◯ Lo mismo que antes ◯ Más alto / más que antes
◯ No aplicable ◯ Prefiero no contestar

Niños jugando / pasando tiempo en teléfonos inteligentes / TV

◯ Más bajo / menos que antes ◯ Lo mismo que antes ◯ Más alto / más que antes
◯ No aplicable ◯ Prefiero no contestar

Actividades físicas de los niños / hacer ejercicio

◯ Más bajo / menos que antes ◯ Lo mismo que antes ◯ Más alto / más que antes
◯ No aplicable ◯ Prefiero no contestar

Arrebato / enojo de los niños

◯ Más bajo / menos que antes ◯ Lo mismo que antes ◯ Más alto / más que antes
◯ No aplicable ◯ Prefiero no contestar

La comunicación de los niños con los amigos

◯ Más bajo / menos que antes ◯ Lo mismo que antes ◯ Más alto / más que antes
◯ No aplicable ◯ Prefiero no contestar

La comunicación de los niños con los miembros de la familia

◯ Más bajo / menos que antes ◯ Lo mismo que antes ◯ Más alto / más que antes
◯ No aplicable ◯ Prefiero no contestar

La vida escolar de los niños

◯ Más bajo / menos que antes ◯ Lo mismo que antes ◯ Más alto / más que antes
◯ No aplicable ◯ Prefiero no contestar

La salud mental general de los niños

◯ Más bajo / menos que antes ◯ Lo mismo que antes ◯ Más alto / más que antes
◯ No aplicable ◯ Prefiero no contestar

La salud física general de los niños

◯ Más bajo / menos que antes ◯ Lo mismo que antes ◯ Más alto / más que antes
◯ No aplicable ◯ Prefiero no contestar

La felicidad de la salud emocional general de los niños

◯ Más bajo / menos que antes ◯ Lo mismo que antes ◯ Más alto / más que antes
◯ No aplicable ◯ Prefiero no contestar

28. Otros problemas (no dude en escribir aquí)

Por favor contáctenos si desea consultar directamente con nuestros miembros de NP
O (Correo electrónico:
info@universalaid.jp; Tel: 090-2900-3934). Deseamos brindar el apoyo necesario a los residentes no japoneses para que puedan llevar una vida cómoda, incluso en este período de COVID-19.

Gracias de antemano por sus amables colaboraciones.

ปัญหาและความวิตกกังวลของผู้ที่ไม่ใช่ชาวญี่ปุ่นที่อาศัยอยู่ในญี่ปุ่นระหว่างสถานการณ์ COVID-19: การสำรวจภาคตัดขวาง

ทาง UNIVERSALAID.JP และ มหาวิทยาลัยนางาซากิ จัดทำการสำรวจนี้ขึ้นเพื่อระบุปัญหาและความวิตกกังวลของผู้ที่ไม่ใช่ชาวญี่ปุ่นที่อาศัยอยู่ในญี่ปุ่น (โดยเฉพาะในเมืองนางาซากิ) ที่เกิดจากการระบาดของ COVID-19 เพื่อค้นหาข้อเสนอแนะและคำปรึกษาที่เหมาะสมสำหรับการเยียวยาปัญหาและความกังวลดังกล่าว

แบบสำรวจประกอบด้วยคำถาม 28 ข้อและใช้เวลาประมาณ 7-10 นาที คุณมีอิสระที่จะปฏิเสธหรือถอนตัวจากการเข้าร่วมได้ตลอดเวลา คำตอบที่ได้จากแบบสำรวจนี้จะเป็นความลับและจะไม่ถูกเปิดเผยไม่ว่ากรณีใด ๆ นอกจากนี้การสำรวจจะไม่มีการระบุชื่อโดยสิ้นเชิงและปราศจากความเสี่ยงใด ๆ

กรุณาเข้าร่วมตอบแบบสอบถามตาม URL หรือ QR Code

https://universalaid.jp/survey2020/

หากมีข้อสงสัยเพิ่มเติมคุณสามารถติดต่อทีมงานของเราโดยส่งอีเมลไปที่

Ms. Yoshimi Matsuo, UNIVERSALAID.JP (Email: info@universalaid.jp; Tel: 090-2900-3934) หรือ Dr. Nguyen Tien Huy, Nagasaki University (Email: tienhuy@nagasaki-u.ac.jp)

การกรอกและกดส่งแบบสำรวจนี้แสดงว่าคุณยินยอมที่จะเข้าร่วมในการสำรวจ

1. คุณอายุมากกว่า 18 ปี และคุณตกลงที่จะเข้าร่วมในโครงการวิจัยนี้หรือไม่?

○ ใช่

○ ไม่

ส่วนที่ 1: ข้อมูลทั่วไป

2. ประเทศที่คุณถือกำเนิดคือประเทศอะไร?

▢ไม่ต้องการตอบ

3. ปัจจุบันคุณอาศัยอยู่ที่ไหน?

- เมืองนางาซากิ
- เมืองอื่น ๆ ของจังหวัดนางาซากิ
- จังหวัดอื่น ๆ
- ไม่ต้องการตอบ

4. ช่วงอายุของคุณ (ปี)

- 18-24 ปี
- 25-34 ปี
- 35-44 ปี
- 45-54 ปี
- 55-64 ปี
- อายุตั้งแต่ 65 ปีขึ้นไป
- ไม่ต้องการตอบ

5. เพศ

- ชาย
- หญิง
- ไม่ต้องการตอบ
- อื่น ๆ (โปรดระบุ)

6. สถานภาพสมรส

- โสดไม่เคยแต่งงาน
- แต่งงานแล้ว หรือ มีแฟนที่อยู่ด้วยกัน
- ม่าย
- หย่าร้าง
- แยกกันอยู่
- ไม่ต้องการตอบ

7. คู่สมรส / แฟนของคุณมีสัญชาติเดิมคือสัญชาติใด?

- ญี่ปุ่น
- สัญชาติเดียวกับตัวเอง
- ฉันไม่มีคู่สมรส / คู่ครอง
- ไม่ต้องการตอบ
- อื่น ๆ (โปรดระบุ)

8. การทำงาน / อาชีพ (เลือกได้มากกว่าหนึ่งอย่าง)

- พนักงานประจำ
- พนักงานพาร์ทไทม์ / รับจ้างทั่วไป
- ประกอบอาชีพอิสระ
- แม่บ้าน / พ่อบ้าน
- ว่างงาน
- นักศึกษา
- เกษียณแล้ว
- ไม่ต้องการตอบ
- อื่น ๆ (โปรดระบุ)

9. การศึกษา

- สำเร็จการศึกษาสูงกว่าระดับปริญญาตรีขึ้นไป
- ระดับวิทยาลัย / มหาวิทยาลัย
- มัธยมปลาย
- มัธยมต้น
- ประถมศึกษา
- ไม่ได้เรียนหนังสือ
- ไม่ต้องการตอบ

10. สถานะการเข้าเมือง

- โอนสัญชาติแล้ว
- อยู่ถาวร
- วีซ่าอนุญาตทำงาน
- วีซ่านักเรียน
- วีซ่าสำหรับครอบครัว
- วีซ่าชั่วคราว (นักท่องเที่ยว, นักธุรกิจ)
- ไม่ต้องการตอบ
- อื่น ๆ (โปรดระบุ)

11. คุณมีประกันสุขภาพประเภทใด? (เลือกได้มากกว่าหนึ่งอย่าง)

- ประกันสุขภาพแห่งชาติ
- ประกันสุขภาพพนักงาน
- ประกันสุขภาพของเอกชน
- ประกันการเดินทาง
- ไม่มี
- ไม่ต้องการตอบ

12. คุณอยู่ญี่ปุ่นมานานแค่ไหน?

- น้อยกว่า 1 ปี
- 1-2 ปี
- 3-5 ปี
- 6-10 ปี
- มากกว่า 10 ปี
- ไม่ต้องการตอบ

13. ภาษาญี่ปุ่นของคุณอยู่ในระดับใด?

- ฉันสามารถพูดได้ในระดับเดียวกับคนญี่ปุ่น
- ฉันสามารถพูดได้ดีพอสำหรับการทำงานหรือการเรียน
- ฉันสามารถพูดได้ดีพอที่จะไม่ทำให้เกิดปัญหาในชีวิตประจำวัน
- ฉันพูดภาษาญี่ปุ่นได้ไม่ดีนัก
- ฉันพูดภาษาญี่ปุ่นไม่ได้เลย
- ไม่ต้องการตอบ

14. จำนวนคนที่อาศัยอยู่ในบ้าน / ห้องของคุณ (รวมทั้งตัวคุณเอง)

▢ 1

▢ 2

▢ 3

▢ 4

▢ 5 คนขึ้นไป

15. จำนวนผู้ใหญ่อายุ 65 ปีขึ้นไปที่อาศัยอยู่กับคุณ

▢ 0

▢ 1

▢ 2

▢ 3

▢ 4

▢ 5 คนขึ้นไป

16. ตอนนี้สมาชิกในครอบครัวคนอื่น ๆ (พ่อแม่ พี่สาว พี่ชาย) อาศัยอยู่ที่ไหน? (เลือกได้มากกว่าหนึ่งข้อ)

- ฉันไม่มีสมาชิกคนอื่นในครอบครัว
- อยู่กับฉันที่ญี่ปุ่น
- อยู่ที่อื่นในญี่ปุ่น
- อยู่ในประเทศบ้านเกิดของฉัน
- อยู่ในประเทศอื่นที่ไม่ใช่ประเทศบ้านเกิด
- ฉันไม่รู้
- ไม่ต้องการตอบ

17. ครั้งสุดท้ายที่คุณพบสมาชิกในครอบครัวคนอื่น ๆ (พ่อแม่ พี่สาว พี่ชาย) คือเมื่อไร?

- น้อยกว่า 1 ปี
- 1-2 ปี
- 3-5 ปี
- 6-10 ปี
- มากกว่า 10 ปี
- ไม่ต้องการตอบ

18. คุณโทรหาสมาชิกในครอบครัวบ่อยแค่ไหน (พ่อแม่ พี่สาว พี่ชาย)?

- ทุกวัน
- ทุกสัปดาห์
- ทุกเดือน
- ปีละไม่กี่ครั้ง
- จำไม่ได้
- ไม่เคยเลย
- ไม่ต้องการตอบ

ส่วนที่ 2: การแลกเปลี่ยนข้อมูลข่าวสาร

19. คุณมีใครที่สามารถพูดคุยเกี่ยวกับปัญหาของคุณไหม?

○ มี

○ ไม่มี

○ ไม่ต้องการตอบ

20. คุณสามารถพูดเรื่องปัญหาของคุณกับใครได้บ้าง? (เลือกได้มากกว่าหนึ่งอย่าง)

- แม่ของฉัน
- พ่อของฉัน
- น้องสาวของฉัน
- น้องชายของฉัน
- ลูกพี่ลูกน้องของฉัน
- ญาติคนอื่น ๆ
- ครู
- เพื่อนร่วมงาน
- เพื่อนชาวญี่ปุ่น
- เพื่อนในประเทศบ้านเกิดของฉัน
- ผู้นำทางศาสนา
- ที่ปรึกษาภาครัฐ
- ไม่ต้องการตอบ
- อื่น ๆ (โปรดระบุ)

21. คุณมักจะหาข้อมูลเกี่ยวกับ COVID-19 จากที่ไหน? (เลือกได้มากกว่าหนึ่งอย่าง)

- ครอบครัวในประเทศบ้านเกิดของคุณ
- ครอบครัวในญี่ปุ่น
- เพื่อนชาวญี่ปุ่น
- เพื่อนจากประเทศบ้านเกิดของคุณ
- องค์กร สถานที่ หรือ ร้านค้าที่ผู้คนมาจากประเทศเดียวกันมารวมตัวกัน
- สำนักงานเทศบาล และจังหวัด
- นิตยสารข้อมูลสาธารณะ และเว็บไซต์ของเทศบาลและจังหวัด
- องค์กรระหว่างประเทศที่ไม่แสวงหาผลกำไร
- ทีวี หนังสือพิมพ์ นิตยสาร หรือ อินเทอร์เน็ต
- สื่อสังคมออนไลน์ (Twitter, Facebook, Instagram และอื่น ๆ)
- ฉันไม่รู้วิธีหาข้อมูลเลย
- ไม่ต้องการตอบ
- อื่น ๆ (โปรดระบุ)

22. คุณต้องการข้อมูลประเภทใดบ้างในภาษาของคุณ? (เลือกได้มากกว่าหนึ่งอย่าง)

- จะทำอย่างไรในกรณีที่สงสัยว่ามีอาการของ COVID-19
- จะทำอย่างไรหากเจ็บป่วย หรือได้รับบาดเจ็บนอกเหนือจาก COVID-19 ในช่วง COVID-19 นี้
- จะทำอย่างไรหากเกิดภัยพิบัติ
- จะทำอย่างไรเมื่อไปโรงพยาบาล / คลินิก
- จะทำอย่างไรในกรณีที่ฉันมีปัญหากับเพื่อนบ้าน เกิดความรุนแรงในครอบครัว หรือเกิดการล่วงละเมิดทางเพศ
- การคลอดบุตร
- จะทำอย่างไรในกรณีที่ลูกของฉันมีปัญหา
- การศึกษาหรือโรงเรียนสำหรับเด็ก
- สถานการณ์ควบคุมการติดเชื้อของ COVID-19
- จุดฮอตสปอต COVID-19 อยู่ที่ไหน
- มาตรการป้องกัน COVID-19
- ความช่วยเหลือทางการเงินที่เกี่ยวข้องกับ COVID-19
- จะกลับประเทศบ้านเกิดได้อย่างไร
- วิธีการต่อวีซ่า
- วิธีโทรหาครอบครัวจากญี่ปุ่น
- การขนส่งสาธารณะ
- วิธีการได้ใบขับขี่
- ระบบภาษีและเงินบำนาญ
- กิจกรรมของชุมชน
- ประสบการณ์หาบ้านเพื่ออยู่อาศัย
- ข้อมูลงาน และ ประสบการณ์หางาน
- วิธีซื้อประกันสุขภาพระดับชาติ และ / หรือ ประกันของเอกชน
- ไม่ต้องการตอบ
- อื่น ๆ (โปรดระบุ)

ส่วนที่ 3: คำถามสั้น ๆ

23. คุณเพิ่ง?

- มีสมาชิกในครอบครัวที่ได้รับการยืนยันป็น COVID-19 หรือไม่?
  - ○ ใช่
  - ○ ไม่
  - ○ ไม่ต้องการตอบ

- มีสมาชิกในครอบครัวที่สงสัยว่าติดเชื้อ COVID-19 แต่ไม่สามารถรับการตรวจได้หรือไม่?
  - ○ ใช่
  - ○ ไม่
  - ○ ไม่ต้องการตอบ

- มีปัญหากับการเรียน หรือ การทำงาน?
  - ○ ใช่
  - ○ ไม่
  - ○ ไม่ต้องการตอบ

- รู้สึกถูกแบ่งแยกเพราะไม่ใช่คนญี่ปุ่น?
  - ○ ใช่
  - ○ ไม่
  - ○ ไม่ต้องการตอบ

- ตกงาน?
  - ○ ใช่
  - ○ ไม่
  - ○ ไม่ต้องการตอบ

- มีความรุนแรงในครอบครัวในบ้านของคุณหรือไม่?
  - ○ ใช่
  - ○ ไม่
  - ○ ไม่ต้องการตอบ

- ถูกคุกคามทางเพศ?

- ใช่
- ไม่
- ไม่ต้องการตอบ

- ทำกิจกรรมทางวิญญาณ / ทางศาสนามากขึ้น?
  - ใช่
  - ไม่
  - ไม่ต้องการตอบ

ส่วนที่ 4: การเปลี่ยนแปลงในชีวิตส่วนบุคคล

24. อธิบายความรู้สึก / อารมณ์ล่าสุด (หรือปัจจุบัน) ของคุณ?

- ฉันรู้สึกสงบ
  - ไม่ใช่เลย
  - ค่อนข้าง
  - ปานกลาง
  - เป็นอย่างมาก

- ฉันเครียด
  - ไม่ใช่เลย
  - ค่อนข้าง
  - ปานกลาง
  - เป็นอย่างมาก

- รู้สึกอารมณ์เสีย
  - ไม่ใช่เลย
  - ค่อนข้าง
  - ปานกลาง
  - เป็นอย่างมาก

- ฉันรู้สึกผ่อนคลาย
  - ไม่ใช่เลย
  - ค่อนข้าง

- ปานกลาง
- เป็นอย่างมาก

- ฉันรู้สึกพอใจ
  - ไม่ใช่เลย
  - ค่อนข้าง
  - ปานกลาง
  - เป็นอย่างมาก

- ฉันกังวล
  - ไม่ใช่เลย
  - ค่อนข้าง
  - ปานกลาง
  - เป็นอย่างมาก

- ฉันรู้สึกเหงา
  - ไม่ใช่เลย
  - ค่อนข้าง
  - ปานกลาง
  - เป็นอย่างมาก

- ฉันรู้สึกโดดเดี่ยว
  - ไม่ใช่เลย
  - ค่อนข้าง
  - ปานกลาง
  - เป็นอย่างมาก

- ฉันกลัวการติดเชื้อ COVID-19
  - ไม่ใช่เลย
  - ค่อนข้าง
  - ปานกลาง
  - เป็นอย่างมาก

25. ระดับของปัญหาของคุณ

- ระยะเวลาการนอนหลับ
  - น้อยกว่าเมื่อก่อน
  - เหมือนเมื่อก่อน
  - มากกว่าเมื่อก่อน
  - ไม่สามารถตอบได้
  - ไม่ต้องการตอบ

- น้ำหนักตัว
  - น้อยกว่าเมื่อก่อน
  - เหมือนเมื่อก่อน
  - มากกว่าเมื่อก่อน
  - ไม่สามารถตอบได้
  - ไม่ต้องการตอบ

- บริโภคเครื่องดื่มแอลกอฮอล์
  - น้อยกว่าเมื่อก่อน
  - เหมือนเมื่อก่อน
  - มากกว่าเมื่อก่อน
  - ไม่สามารถตอบได้
  - ไม่ต้องการตอบ

- สูบบุหรี่
  - น้อยกว่าเมื่อก่อน
  - เหมือนเมื่อก่อน
  - มากกว่าเมื่อก่อน
  - ไม่สามารถตอบได้
  - ไม่ต้องการตอบ

- เล่นเกม / ใช้เวลาบนสมาร์ทโฟน / ทีวี
  - น้อยกว่าเมื่อก่อน
  - เหมือนเมื่อก่อน
  - มากกว่าเมื่อก่อน
  - ไม่สามารถตอบได้

- ไม่ต้องการตอบ

- กิจกรรมทางกายภาพ / การออกกำลังกาย
  - น้อยกว่าเมื่อก่อน
  - เหมือนเมื่อก่อน
  - มากกว่าเมื่อก่อน
  - ไม่สามารถตอบได้
  - ไม่ต้องการตอบ

- ระยะเวลาการเลี้ยงดู หรือ การดูแลเด็ก
  - น้อยกว่าเมื่อก่อน
  - เหมือนเมื่อก่อน
  - มากกว่าเมื่อก่อน
  - ไม่สามารถตอบได้
  - ไม่ต้องการตอบ

- ความขัดแย้ง / ทะเลาะกันในบ้านของคุณ
  - น้อยกว่าเมื่อก่อน
  - เหมือนเมื่อก่อน
  - มากกว่าเมื่อก่อน
  - ไม่สามารถตอบได้
  - ไม่ต้องการตอบ

- การสื่อสารกับเพื่อนบ้าน หรือ เพื่อน
  - น้อยกว่าเมื่อก่อน
  - เหมือนเมื่อก่อน
  - มากกว่าเมื่อก่อน
  - ไม่สามารถตอบได้
  - ไม่ต้องการตอบ

- สุขภาพจิตโดยรวมของคุณ
  - น้อยกว่าเมื่อก่อน
  - เหมือนเมื่อก่อน
  - มากกว่าเมื่อก่อน

- ไม่สามารถตอบได้
- ไม่ต้องการตอบ

- สุขภาพร่างกายโดยรวมของคุณ
  - น้อยกว่าเมื่อก่อน
  - เหมือนเมื่อก่อน
  - มากกว่าเมื่อก่อน
  - ไม่สามารถตอบได้
  - ไม่ต้องการตอบ

- สุขภาพทางอารมณ์โดยรวมของคุณ (ความสุข)
  - น้อยกว่าเมื่อก่อน
  - เหมือนเมื่อก่อน
  - มากกว่าเมื่อก่อน
  - ไม่สามารถตอบได้
  - ไม่ต้องการตอบ

- ความพึงพอใจทางเพศโดยรวมของคุณ
  - น้อยกว่าเมื่อก่อน
  - เหมือนเมื่อก่อน
  - มากกว่าเมื่อก่อน
  - ไม่สามารถตอบได้
  - ไม่ต้องการตอบ

- รายได้ส่วนบุคคล
  - น้อยกว่าเมื่อก่อน
  - เหมือนเมื่อก่อน
  - มากกว่าเมื่อก่อน
  - ไม่สามารถตอบได้
  - ไม่ต้องการตอบ

- ความมั่นคงทางการเงินโดยรวมของครัวเรือนของคุณ
  - น้อยกว่าเมื่อก่อน
  - เหมือนเมื่อก่อน

- มากกว่าเมื่อก่อน
- ไม่สามารถตอบได้
- ไม่ต้องการตอบ

26. จำนวนเด็กอายุต่ำกว่า 18 ปีที่อาศัยอยู่กับคุณ

▢ 0

▢ 1

▢ 2

▢ 3

▢ 4

▢ 5 คนขึ้นไป

27. ระดับของปัญหาเด็กของคุณ

- ระยะเวลาการนอนหลับของเด็ก
    - ต่ำกว่า / น้อยกว่าเดิม
    - เหมือนเมื่อก่อน
    - สูงขึ้น / มากกว่าเดิม
    - ไม่สามารถตอบได้
    - ไม่ต้องการตอบ

- เด็กเล่นเกม / ใช้เวลากับสมาร์ทโฟน / ทีวี
    - ต่ำกว่า / น้อยกว่าเดิม
    - เหมือนเมื่อก่อน
    - สูงขึ้น / มากกว่าเดิม
    - ไม่สามารถตอบได้
    - ไม่ต้องการตอบ

- กิจกรรมทางกาย / การออกกำลังกายของเด็ก ๆ
    - ต่ำกว่า / น้อยกว่าเดิม
    - เหมือนเมื่อก่อน
    - สูงขึ้น / มากกว่าเดิม
    - ไม่สามารถตอบได้
    - ไม่ต้องการตอบ

- ความรุนแรง / ความโกรธ ของเด็ก
  - ต่ำกว่า / น้อยกว่าเดิม
  - เหมือนเมื่อก่อน
  - สูงขึ้น / มากกว่าเดิม
  - ไม่สามารถตอบได้
  - ไม่ต้องการตอบ

- การสื่อสารของเด็กกับเพื่อน ๆ
  - ต่ำกว่า / น้อยกว่าเดิม
  - เหมือนเมื่อก่อน
  - สูงขึ้น / มากกว่าเดิม
  - ไม่สามารถตอบได้
  - ไม่ต้องการตอบ

- การสื่อสารของเด็ก ๆ กับสมาชิกในครอบครัว
  - ต่ำกว่า / น้อยกว่าเดิม
  - เหมือนเมื่อก่อน
  - สูงขึ้น / มากกว่าเดิม
  - ไม่สามารถตอบได้
  - ไม่ต้องการตอบ

- ชีวิตในโรงเรียนของเด็ก ๆ
  - ต่ำกว่า / น้อยกว่าเดิม
  - เหมือนเมื่อก่อน
  - สูงขึ้น / มากกว่าเดิม
  - ไม่สามารถตอบได้
  - ไม่ต้องการตอบ

- สุขภาพจิตโดยรวมของเด็ก
  - ต่ำกว่า / น้อยกว่าเดิม
  - เหมือนเมื่อก่อน
  - สูงขึ้น / มากกว่าเดิม
  - ไม่สามารถตอบได้

– ไม่ต้องการตอบ

- สุขภาพร่างกายโดยรวมของเด็ก
  – ต่ำกว่า / น้อยกว่าเดิม
  – เหมือนเมื่อก่อน
  – สูงขึ้น / มากกว่าเดิม
  – ไม่สามารถตอบได้
  – ไม่ต้องการตอบ

- ความสุขทางด้านความรู้สึกโดยรวมของเด็ก ๆ
  – ต่ำกว่า / น้อยกว่าเดิม
  – เหมือนเมื่อก่อน
  – สูงขึ้น / มากกว่าเดิม
  – ไม่สามารถตอบได้
  – ไม่ต้องการตอบ

28. ปัญหาอื่น ๆ (โปรดเขียนที่นี่)

โปรดติดต่อเราหากคุณต้องการปรึกษาโดยตรงกับสมาชิก NPO ของเรา

(Email: info@universalaid.jp; Tel: 090-2900-3934)

เราต้องการให้การสนับสนุนที่จำเป็นแก่ผู้ที่ไม่ใช่คนญี่ปุ่นที่อาศัยอยู่ในญี่ปุ่น เพื่อให้มีชีวิตที่สะดวกสบายแม้ว่าจะอยู่ภายใต้ช่วงเวลา COVID-19 นี้ก็ตาม

ขอขอบคุณล่วงหน้าสำหรับความร่วมมือที่ดี

Các vấn đề và mối lo ngại của người dân ngoại quốc đang cư trú và sinh sống tại Nhật trong đại dịch COVID-19: nghiên cứu cắt ngang

Chúng tôi, UNIVERSALAID.JP và trường Đại học Nagasaki, sẽ thực hiện cuộc khảo sát này để nhận định các vấn đề và mối lo ngại của người dân ngoại quốc đang cư trú và sinh sống tại Nhật (đặc biệt là thành phố Nagasaki) trong đại dịch COVID-19, với nỗ lực mang đến gợi ý và tư vấn để giải quyết các khó khăn và lo lắng này.

Khảo sát gồm 28 câu hỏi và có thể mất khoảng 7-10 phút để hoàn thành. Anh chị có quyền từ chối tham gia hoặc rút khỏi nghiên cứu này bất cứ lúc nào. Tất cả các câu trả lời thu thập được trong khảo sát sẽ được giữ bí mật và không được tiết lộ dưới mọi điều kiện. Khảo sát sẽ hoàn toàn được ghi lại ẩn danh và không có rủi ro liên quan.

Vui lòng tham gia khảo sát qua URL hoặc mã QR dưới đây: https://universalaid.jp/survey2020/

Đối với bất kỳ thắc mắc nào khác, anh chị có thể liên hệ với nhóm của chúng tôi bằng cách gửi email cho Bà Yoshimi Matsuo, UNIVERSALAID.JP (Email: info@universalaid.jp; SĐT: 090-2900-3934) hoặc Tiến sĩ Nguyễn Tiến Huy, Đại học Nagasaki (Email: tienhuy@nagasaki-u.ac.jp)

Bằng cách hoàn thành và gửi khảo sát này, anh chị đã đồng ý tham gia vào nghiên cứu.

1. Anh chị có trên 18 tuổi và đồng ý tham gia vào nghiên cứu này không?
   - ☐ Có
   - ☐ Không

Phần 1: THÔNG TIN CHUNG

2. Anh chị có nguyên quán tại nước nào?

☐ ______________________________

☐ Không muốn trả lời

3. Anh chị đang sống ở đâu?

☐ Thành phố Nagasaki
☐ Thành phố khác ở tỉnh Nagasaki
☐ Các tỉnh khác
☐ Không muốn trả lời

4. Tuổi của anh chị trong khoảng:

☐ 18-24 tuổi
☐ 25-34 tuổi
☐ 35-44 tuổi
☐ 45-54 tuổi
☐ 55-64 tuổi
☐ 65 tuổi trở lên
☐ Không muốn trả lời

5. Giới tính của anh chị:

☐ Nam
☐ Nữ
☐ Không muốn trả lời
☐ Khác (vui lòng cụ thể)

6. Tình trạng hôn nhân của anh chị

☐ Độc thân, chưa từng kết hôn
☐ Đã kết hôn hoặc sống chung như vợ chồng
☐ Goá (chồng, vợ)
☐ Đã ly hôn
☐ Đang ly thân
☐ Không muốn trả lời

7. Quốc tịch của chồng/vợ/người yêu của anh chị?

☐ Nhật
☐ Cùng quốc tịch với tôi
☐ Quốc tịch khác
☐ Tôi không có chồng/vợ/người yêu
☐ Không muốn trả lời

8. Nghề nghiệp của anh chị (chọn một hoặc nhiều hơn)

☐ Nhân viên toàn thời gian
☐ Nhân viên bán thời gian/ công việc thời vụ
☐ Tự làm chủ
☐ Nội trợ
☐ Thất nghiệp

- [ ] Sinh viên
- [ ] Nghỉ hưu
- [ ] Không muốn trả lời
- [ ] Khác (vui lòng cụ thể)

____

9. Học vấn của anh chị
   - [ ] Sau đại học hoặc cao hơn
   - [ ] Đại học/ Cao đẳng
   - [ ] Trung học phổ thông
   - [ ] Trung học cơ sở
   - [ ] Tiểu học
   - [ ] Không có
   - [ ] Không muốn trả lời

10. Tình trạng nhập cư của anh chị
    - [ ] Đã nhập tịch
    - [ ] Thường trú nhân
    - [ ] Visa dạng lao động
    - [ ] Visa dạng du học
    - [ ] Visa dạng bảo lãnh gia đình
    - [ ] Visa tạm thời (du lịch, kinh doanh)
    - [ ] Không muốn trả lời
    - [ ] Khác (vui lòng cụ thể)

____

11. Anh chị có bảo hiểm y tế loại gì? (chọn một hoặc nhiều hơn)
    - [ ] Bảo hiểm y tế nhà nước
    - [ ] Bảo hiểm y tế dạng nhân viên
    - [ ] Bảo hiểm y tế tư nhân
    - [ ] Bảo hiểm y tế du lịch
    - [ ] Không có
    - [ ] Không muốn trả lời

12. Anh chị đã sinh sống tại Nhật Bản trong bao lâu?
    - [ ] Dưới 1 năm
    - [ ] 1-2 năm
    - [ ] 3-5 năm
    - [ ] 6-10 năm
    - [ ] Hơn 10 năm
    - [ ] Không muốn trả lời

13. Trình độ tiếng Nhật của anh chị:

- ☐ Tôi có thể nói trình độ như người Nhật
- ☐ Tôi có thể nói đủ tốt cho mục đích làm việc và học tập
- ☐ Tôi có thể nói đủ tốt mà không gặp vấn đề trong cuộc sống hằng ngày
- ☐ Tôi không thể nói tiếng Nhật tốt
- ☐ Tôi hoàn toàn không thể nói tiếng Nhật
- ☐ Không muốn trả lời

14. Số lượng người sống cùng nhà/phòng với bạn (bao gồm cả bản thân bạn)?

- ☐ 0
- ☐ 1
- ☐ 2
- ☐ 3
- ☐ 4
- ☐ 5 hoặc nhiều hơn

15. Số lượng người 65 tuổi hoặc trên 65 tuổi đang sống với anh chị:

- ☐ 0
- ☐ 1
- ☐ 2
- ☐ 3
- ☐ 4
- ☐ 5 hoặc nhiều hơn

16. Các thành viên trong gia đình anh chị (cha mẹ, anh chị em) hiện đang cư trú tại đâu? (chọn một hoặc nhiều hơn)

- ☐ Tôi không có người thân nào
- ☐ Với tôi tại Nhật
- ☐ Tại nơi khác ở Nhật
- ☐ Quê hương
- ☐ Nước khác
- ☐ Tôi không biết
- ☐ Không muốn trả lời

17. Lần cuối anh chị gặp các thành viên trong gia đình (cha mẹ, anh chị em) là khi nào?

- ☐ Dưới 1 năm
- ☐ 1-2 năm
- ☐ 3-5 năm
- ☐ 6-10 năm
- ☐ Hơn 10 năm
- ☐ Không muốn trả lời

18. Tần suất anh chị gọi điện thoại với thành viên trong gia đình (cha mẹ, anh chị em)

☐ Hằng ngày
☐ Hằng tuần
☐ Hằng tháng
☐ Vài lần một năm
☐ Không thể nhớ
☐ Không bao giờ
☐ Không muốn trả lời

Phần 2: MẠNG LƯỚI THÔNG TIN

19. Anh chị có ai để chia sẻ các vấn đề của mình không?

☐ Có
☐ Không
☐ Không muốn trả lời
☐ Khác (vui lòng cụ thể)

____

20. Anh chị có thể chia sẻ khó khăn được với những ai? (chọn một hoặc nhiều hơn)

☐ Mẹ

☐ Ba

☐ Chị/em gái

☐ Anh/em trai

☐ Anh chị em họ

☐ Những người thân thích khác

☐ Giáo viên

☐ Đồng nghiệp

☐ Bạn người Nhật

☐ Bạn bè ở nơi quê nhà

☐ Người đứng đầu tôn giáo của anh chị

☐ Chuyên gia tư vấn của chính phủ

☐ Không muốn trả lời

☐ Khác (vui lòng cụ thể)

____

21. Anh chị lấy thông tin về COVID-19 ở đâu? (chọn một hoặc nhiều hơn)

☐ Gia đình ở quê nhà

☐ Gia đình ở Nhật

☐ Bạn bè người Nhật

☐ Đồng hương

☐ Tổ chức, cơ sở, hay cửa hàng đồng hương hay tụ tập

☐ Văn phòng tỉnh và thành phố

☐ Tạp chí thông tin công cộng và trang web của các thành phố và tỉnh

☐ Tổ chức trao đổi quốc tế phi lợi nhuận (NPO)

☐ Tivi, báo, tạp chí, hay Internet

☐ SNS (Twitter, Facebook, Instagram, …)

☐ Tôi không biết lấy thông tin như thế nào

☐ Không muốn trả lời

☐ Khác (vui lòng cụ thể)

____

22. Anh chị mong muốn thông tin nào được công bố bằng ngôn ngữ của bản thân? (chọn một hoặc nhiều hơn)

☐ Phải làm gì trong trường hợp tôi có một triệu chứng nghi ngờ của COVID-19

☐ Phải làm gì trong trường hợp bị ốm hoặc bị thương ngoài COVID-19 trong giai đoạn COVID-19 này

☐ Làm gì trong trường hợp thiên tai

☐ Làm gì khi đến bệnh viện / phòng khám

☐ Phải làm gì trong trường hợp tôi gặp rắc rối với hàng xóm, bạo lực gia đình hoặc quấy rối tình dục

☐ Sinh con

☐ Phải làm gì trong trường hợp con tôi gặp sự cố

☐ Giáo dục, trường học cho trẻ em

☐ Tình hình kiểm soát nhiễm COVID-19

☐ Vị trí các điểm nóng COVID-19

☐ Các biện pháp phòng ngừa chống lại COVID-19

☐ Hỗ trợ tài chính liên quan đến COVID-19

☐ Làm thế nào để trở về quê hương

☐ Cách gia hạn visa

☐ Cách gọi cho gia đình từ Nhật Bản

☐ Giao thông công cộng

☐ Làm thế nào để có được giấy phép lái xe

☐ Hệ thống thuế và lương hưu

☐ Sự kiện cộng đồng

☐ Kinh nghiệm tìm nhà để ở

☐ Thông tin việc làm và kinh nghiệm tìm việc

☐ Cách mua bảo hiểm y tế quốc gia và / hoặc tư nhân

☐ Không muốn trả lời

☐ Khác (vui lòng cụ thể)

___

Phần 3: CÂU HỎI NGẮN – Gần đây anh chị đã từng?

23. Gần đây anh chị đã từng:

	Có	Không	Tôi không muốn trả lời
Có một thành viên trong gia đình bị nhiễm COVID-19	☐	☐	☐
Có một thành viên trong gia đình nghi bị nhiễm COVID-19 nhưng không thể xét	☐	☐	☐

nghiệm kiểm tra được			
Gặp rắc rối/khó khăn trong việc học tập hoặc làm việc	☐	☐	☐
Cảm thấy bị phân biệt đối xử khi không phải là người Nhật	☐	☐	☐
Mất việc	☐	☐	☐
Xảy ra bạo lực gia đình ở nhà	☐	☐	☐
Bị xâm hại tình dục	☐	☐	☐
Cảm thấy có tinh thần hơn/ hoạt động tôn giáo nhiều hơn	☐	☐	☐

Phần 4: THAY ĐỔI TRONG CUỘC SỐNG CÁ NHÂN

24. Mô tả các cảm giác/ tâm trạng của anh chị gần đây (hoặc hiện tại)?

	Hoàn toàn không	Một chút	Vừa phải	Rất nhiều
Tôi bình tĩnh	☐	☐	☐	☐
Tôi căng thẳng	☐	☐	☐	☐
Tôi thấy buồn	☐	☐	☐	☐
Tôi cảm thấy thư giãn	☐	☐	☐	☐
Tôi cảm thấy hài lòng	☐	☐	☐	☐
Tôi cảm thấy lo lắng	☐	☐	☐	☐

Tôi cảm thấy cô đơn	☐	☐	☐	☐
Tôi cảm thấy bị cô lập	☐	☐	☐	☐
Tôi sợ bị nhiễm COVID-19	☐	☐	☐	☐

25. Mức độ các vấn đề của anh chị

	Ít hơn lúc trước	Như lúc trước	Nhiều hơn trước	Không áp dụng	Tôi không muốn trả lời
Thời gian ngủ	☐	☐	☐	☐	☐
Cân nặng cơ thể	☐	☐	☐	☐	☐
Sử dụng rượu	☐	☐	☐	☐	☐
Hút thuốc	☐	☐	☐	☐	☐
Thời gian chơi game/sử dụng điện thoại/TV	☐	☐	☐	☐	☐
Hoạt động thể chất/tập thể dục	☐	☐	☐	☐	☐
Thời gian nuôi dạy hoặc chăm sóc con cái	☐	☐	☐	☐	☐
Xung đột/cãi vã ở gia đình của anh chị	☐	☐	☐	☐	☐

Giao tiếp với hàng xóm hay bạn bè	☐	☐	☐	☐	☐
Sức khoẻ tinh thần chung của anh chị	☐	☐	☐	☐	☐
Sức khoẻ thể chất chung của anh chị	☐	☐	☐	☐	☐
Sức khỏe cảm xúc chung (sự hạnh phúc) của anh chị	☐	☐	☐	☐	☐
Sự thoả mãn tình dục của anh chị	☐	☐	☐	☐	☐
Thu nhập cá nhân	☐	☐	☐	☐	☐
Sự ổn định tài chính chung của gia đình anh chị	☐	☐	☐	☐	☐

26. Có bao nhiêu trẻ dưới 18 tuổi sống chung với anh chị

☐0
☐1
☐2
☐3
☐4
☐ Nhiều hơn 4

Phần 5: THAY ĐỔI TRONG HÀNH VI CON CÁI CỦA ANH CHỊ

27. Mức độ các vấn đề của con anh chị

	Thấp hơn/ít hơn lúc trước	Giống như lúc trước	Cao hơn/nhiều hơn trước	Không áp dụng	Tôi không muốn trả lời
Thời gian ngủ của con cái	☐	☐	☐	☐	☐
Thời gian con cái chơi game/sử dụng điện thoại/TV	☐	☐	☐	☐	☐
Hoạt động thể chất /tập thể dục của con cái	☐	☐	☐	☐	☐
Sự bộc phát/ giận dữ của con cái	☐	☐	☐	☐	☐
Giao tiếp với bạn bè	☐	☐	☐	☐	☐
Giao tiếp với các thành viên gia đình	☐	☐	☐	☐	☐
Thời gian học ở trường	☐	☐	☐	☐	☐
Sức khoẻ tinh thần chung của con cái	☐	☐	☐	☐	☐
Sức khoẻ thể chất chung của con cái	☐	☐	☐	☐	☐
Sức khỏe cảm xúc chung (sự	☐	☐	☐	☐	☐

hạnh phúc)<br>của con cái	

28. Khó khăn khác (vui lòng viết ở đây)

____

Vui lòng liên hệ với chúng tôi nếu anh chị mong muốn trực tiếp tham vấn với các thành viên NPO của chúng tôi (Email: info@universalaid.jp; Điện thoại: 090-2900-3934). Chúng tôi mong muốn cung cấp sự hỗ trợ cần thiết cho các người dân ngoại quốc tại Nhật để mang đến một cuộc sống thoải mái, ngay cả trong thời kỳ COVID-19.

Xin cảm ơn sự cộng tác của anh chị.
